# On a Countable Sequence of Homoclinic Orbits Arising Near a Saddle–Center Point

**DOI:** 10.1007/s00220-025-05381-8

**Published:** 2025-08-01

**Authors:** Inmaculada Baldomá, Marcel Guardia, Dmitry E. Pelinovsky

**Affiliations:** 1 Departament de Matemàtiques and IMTECH, Universitat Politècnica de Catalunya, Diagonal 647, 08028 Barcelona, Spain; 2 Centre de Recerca Matemàtica, Campus de Bellaterra, Edifici C, 08193 Barcelona, Spain; 3 Departament de Matemàtiques i Informàtica, Universitat de Barcelona, Gran Via, 585, 08007 Barcelona, Spain; 4 Department of Mathematics and Statistics, McMaster University, L8S 4K1 Hamilton, ON Canada

## Abstract

Exponential small splitting of separatrices in the singular perturbation theory leads generally to nonvanishing oscillations near a saddle–center point and to nonexistence of a true homoclinic orbit. It was conjectured long ago that the oscillations may vanish at a countable set of small parameter values if there exist a quadruplet of singularities in the complex analytic extension of the limiting homoclinic orbit. The present paper gives a rigorous proof of this conjecture for a particular fourth-order equation relevant to the traveling wave reduction of the modified Korteweg–de Vries equation with the fifth-order dispersion term.

## Introduction

Homoclinic orbits arise in dynamical systems at the intersections of stable and unstable manifolds (also known as the separatrices) associated to a saddle equilibrium point. They represent spatial profiles of traveling solitary waves in nonlinear dispersive wave equations from which spatial dynamical systems are obtained in the traveling reference frame. Existence of a homoclinic orbit connected at a saddle point is a generic phenomena in a planar Hamiltonian system if there exists a center point near the saddle point.

The phase space of many spatial dynamical systems has the dimension higher than two, in which case the equilibrium point may admit a center manifold in addition to the stable and unstable manifolds. For such a saddle-center point, intersection of the separatrices is not generic and homoclinic orbits do not generally exist. The corresponding traveling solitary waves are not fully decaying since their spatial profiles approach the oscillatory tails spanned by orbits along the center manifold.

It is rather common in analysis of solitary waves to consider an asymptotic limit when a higher-dimensional spatial dynamical system with a saddle-center point formally reduces to the planar Hamiltonian dynamical system with a homoclinic orbit. This leads to the main question of the singular perturbation theory if the homoclinic orbit persists under the perturbation. The standard answer to this question is negative because the exponentially small splitting of the separatrices generally occurs due to the singular perturbations.

First examples of the exponentially small (beyond-all-order) phenomena and the relevant asymptotic analysis can be found in [13, 16, 26, 32, 36, 48]. Rigorous mathematical analysis and the proof of the existence of oscillatory tails near the saddle-center point in four-dimensional spatial dynamical systems was later developed in [40, 51]. The oscillatory tails are present if a certain constant (called the Stokes constant) is nonzero, the proof of which usually relies on numerical computations. The numerical data in [52] for a particular model of the fifth-order Korteweg–de Vries (KdV) equation suggest that the Stokes constant is generally nonzero but may vanish along bifurcations of co-dimension one if another parameter is present in the spatial dynamical system.

Compared to the standard setting of the non-vanishing oscillatory tails in the beyond-all-order expansions, a rather novel mechanism of obtaining a countable number of true homoclinic orbits was proposed in [3]. The mechanism is related to the location of singularities of the truncated homoclinic orbit in a complex plane. If there is only one symmetric pair of singularities in the complex plane nearest to the real line, then the Stokes constant is generally nonzero and no true homoclinic orbit persists in the singular perturbation theory. However, if there exist a quadruplet with two symmetric pairs of singularities at the same distance from the real line, then the singular perturbation theory exhibits a countable set of true homoclinic orbits as the small parameter goes to zero.

The theory from [3] was illustrated on a number of other mathematical models involving nonlocal integral equations [2], lattice advance-delay equations [1, 45], and differential advance-delay equations for traveling waves in lattices [19, 20, 41, 42]. The spatial profiles of solitary waves in such models must generally exhibit oscillatory tails (in which case, they are usually called generalized solitary waves or nanoptera), see analysis in [21, 23] and numerical results in [22, 42, 54]. However, the tails miraculously vanish along a countable set of bifurcation points if the singular limit admits a real analytic solution with a quadruplet of complex singularities nearest to the real line. A similar idea for homoclinic orbits in symplectic discrete maps has been discussed in [27] some time before [3], see also analysis of splitting of separatrices in the presence of several singularities in [38] and in [29].

Despite a number of examples supporting the conjecture from [3], no mathematically rigorous proof was developed in the literature. The purpose of this paper is to give a proof of this conjecture for the simplest four-dimensional dynamical system with a saddle-center equilibrium point.

### Main model

Let $\gamma , \varepsilon \in {\mathbb {R}}$ be parameters and consider the fourth-order equation for some $u \in C^{\infty }(\mathbb {R},\mathbb {R})$,

1.1 $$\begin{aligned} \varepsilon ^2 u''''+(1-\varepsilon ^2)u''-u+u^2+2\gamma u^3=0. \end{aligned}$$

If $\varepsilon$ is a small parameter, then the formal limit $\varepsilon \rightarrow 0$ yields the second-order equation

1.2 $$\begin{aligned} u''-u+u^2+2\gamma u^3=0 \end{aligned}$$

with (0, 0) being a saddle point of the planar Hamiltonian system

1.3 $$\begin{aligned} \left\{ \begin{array}{l} u' = w, \\ w' = u-u^2-2\gamma u^3. \end{array} \right. \end{aligned}$$

The second-order equation (1.2) appears in the traveling wave reduction of the modified Korteweg–de Vries (KdV) equation

1.4 $$\begin{aligned} \frac{\partial \eta }{\partial t} + 2 \eta \frac{\partial \eta }{\partial x} + 6 \beta \eta ^2 \frac{\partial \eta }{\partial x} + \frac{\partial ^3 \eta }{\partial x^3} = 0, \end{aligned}$$

where $\eta = \eta (x,t)$ is real and $\beta$ is a parameter. Traveling waves of the modified KdV equation (1.4) correspond to the form $\eta (x,t) = \eta _c(x-ct)$ with the wave speed *c* and the wave profile $\eta _c$ found from the third-order equation

1.5 $$\begin{aligned} \eta _c'''(x) - c \eta _c'(x) + 2 \eta _c \eta _c'(x) + 6 \beta \eta _c^2 \eta _c'(x) = 0. \end{aligned}$$

If $c > 0$, the scaling transformation $\eta _c(x) = c u(\sqrt{c} x)$ and integration of (1.5) with zero integration constant for solitary wave solutions yields equation (1.2) with $\gamma := \beta c$.

**Fig. 1:**
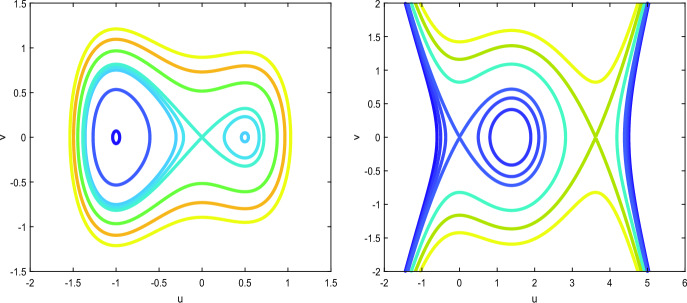
Phase portraits of (1.3) for $\gamma = 1$ (left) and $\gamma = -0.1$ (right)

If $\gamma > 0$, there exist two families of periodic solutions and two solitary wave solutions of equation (1.2), see, e.g., [14, 39]. If $\gamma < 0$, there exists only one family of periodic solutions and only one solitary wave solution of equation (1.2), see, e.g., [46]. This also follows from the phase portraits for the dynamical system (1.3) on the phase plane (*u*, *w*) shown in Fig. 1 for $\gamma = 1$ (left) and $\gamma = -0.1$ (right).

The fourth-order equation (1.1) is the traveling wave reduction of the modified KdV equation with the fifth-order dispersion term, also known as the Kawahara equation [35],

1.6 $$\begin{aligned} \frac{\partial \eta }{\partial t} + 2 \eta \frac{\partial \eta }{\partial x} + 6 \beta \eta ^2 \frac{\partial \eta }{\partial x} + \frac{\partial ^3 \eta }{\partial x^3} + \alpha \frac{\partial ^5 \eta }{\partial x^5} = 0, \end{aligned}$$

where $\alpha$ is another parameter. Traveling waves of the form $\eta (x,t) = \eta _c(x-ct)$ satisfy the fifth-order equation, which can be integrated once with the zero integration constant. The scaling transformation $\eta _c(x) = c u(\sqrt{c(1-\varepsilon ^2)} x)$ yields (1.1) with $\gamma = \beta c$ and $\varepsilon ^2$ found from the equation

$$ \frac{\varepsilon ^2}{(1-\varepsilon ^2)^2} = \alpha c. $$

This is always possible for small $\varepsilon$ if $\alpha c$ is small.

For $\beta = 0$, the Kawahara equation (1.6) has been one of the main toy model of the shallow water wave theory to study periodic oscillations arising at the exponential tails of the solitary wave profiles, see recent works [15, 34, 50]. Since the true homoclinic orbits are known not to exist for $\beta = 0$ [32, 48], the main motivation for our study is to show the existence of a sequence of true homoclinic orbits in the modified Kawahara equation for $\beta \ne 0$.

The homoclinic orbit of the second-order system (1.3) with $\gamma = 0$ is known in the exact analytical form:

$$\begin{aligned} u_0(x) = \frac{3}{2} \textrm{sech}^2\left( \frac{x}{2}\right) . \end{aligned}$$

The profile of $u_0$ has double poles on the imaginary axis with the nearest singularities at $x = \pm i \pi$. If $\gamma \ne 0$, the double poles split into pairs of simple poles and the splitting is different for $\gamma > 0$ and $\gamma < 0$. The homoclinic orbit for $\gamma = 0$ is continued in the exact analytical form for every $1 + 9 \gamma > 0$ as

1.7 $$\begin{aligned} u_0(x)=\frac{3}{\sqrt{1+ 9\gamma }\cosh (x)+1}. \end{aligned}$$

For $\gamma > 0$, the poles of $u_0$ nearest to the real axis split along the imaginary axis as simple poles at

$$\begin{aligned} x = \pm i \pi \pm i \arccos \left( \frac{1}{\sqrt{1+9\gamma }}\right) , \end{aligned}$$

with four independent choices of signs. For $\gamma \in (-\frac{1}{9},0)$ the poles of $u_0$ split off the imaginary axis as simple poles at

1.8 $$\begin{aligned} x = \pm i \pi \pm \cosh ^{-1}\frac{1}{\sqrt{1+9\gamma }}, \end{aligned}$$

again with four independent choices of signs. This is precisely the case which fits the theory from [3] and coincides with Example 1 in [3]. The numerical data on Figure 1 in [3] already provide a convining evidence of the existence of a countable sequence $\{ \varepsilon _n(\gamma ) \}_{n \in \mathbb {N}}$ for every $\gamma \in (-\frac{1}{9},0)$ such that $\varepsilon _n(\gamma ) \rightarrow 0$ as $n \rightarrow \infty$ with the homoclinic orbits persisting in the full equation (1.1) for $\varepsilon = \varepsilon _n(\gamma )$ and with *u*(*x*) being close to $u_0(x)$ in (1.7).

Hence, in what follows we are only interested in the case $\gamma \in (-\frac{1}{9},0)$, when the only homoclinic orbit with the profile $u_0$ is available in the form (1.7). For completeness, we mention that another homoclinic orbit exists for $\gamma > 0$, see the left panel of Fig. 1, and its (negative) profile is given by

$$\begin{aligned} {{\tilde{u}}}_0(x)=-\frac{3}{\sqrt{1+ 9\gamma }\cosh (x)-1}. \end{aligned}$$

The simple poles of ${\tilde{u}}_0$ are located at the imaginary axis at

$$\begin{aligned} x = \pm i \arccos \left( \frac{1}{\sqrt{1+9\gamma }}\right) + 2\pi i n, \quad n \in \mathbb {Z}. \end{aligned}$$

For $\gamma \le 0$, ${\tilde{u}}_0$ is singular on real line and hence is neglected.

### Main result and the method of proof

The main result of this paper is the following.

#### Theorem 1.1

For any $\gamma \in \left( -\frac{1}{9},0 \right)$, there exists $N_0\in \mathbb {N}$ large enough and a sequence $\{\varepsilon _n\}_{n\ge N_0}$ of the form

1.9 $$\begin{aligned} \varepsilon _n = \frac{\alpha }{n\pi } \left[ 1 + \frac{1}{n} \mathcal {O}\left( \frac{1}{\log n}\right) \right] ,\qquad \text {where}\quad \alpha =\cosh ^{-1}\frac{1}{\sqrt{1+9\gamma }}, \end{aligned}$$

such that equation (1.1) with $\varepsilon =\varepsilon _n$ has a homoclinic orbit to the origin in ${\mathbb {R}}^4$.

We prove this result by analyzing the stable and unstable invariant manifolds of the origin in ${\mathbb {R}}^4$ and measuring their distance at a suitable cross-section of ${\mathbb {R}}^4$. To this end, we rewrite the fourth-order equation (1.1) as two second-order equations. By introducing

1.10 $$\begin{aligned} f(u):= u^2 + 2 \gamma u^3\quad \text { and }\quad v:= u''-u+f(u), \end{aligned}$$

equation (1.1) becomes the system

1.11 $$\begin{aligned} \left\{ \begin{array}{l} u''=u+v-f(u)\\ v''=-\frac{1}{\varepsilon ^2}v+f'(u) (u+v-f(u))+f''(u)(u')^2. \end{array} \right. \end{aligned}$$

The phase space of system (1.11) is written in the variables $(u,u',v,v')\in {\mathbb {R}}^4$. Moreover, this system has the first integral

1.12 $$\begin{aligned} \begin{aligned} G(u,u',v,v')=&(1-\varepsilon ^2)\frac{(u')^2}{2}-\frac{u^2}{2}+F(u)\\&+\varepsilon ^2\left[ u'(v'+u'-f'(u)u')-\frac{(u+v-f(u))^2}{2}\right] , \end{aligned} \end{aligned}$$

with

$$\begin{aligned} F(u)=\int _{0}^u f(v)dv =\frac{u^3}{3}+ \frac{\gamma u^4}{2}. \end{aligned}$$

We notice that the origin in ${\mathbb {R}}^4$ is a saddle-center equilibrium point of the second-order system (1.11) with associated eigenvalues $\big \{ -1, 1, i \varepsilon ^{-2}, -i \varepsilon ^{-2}\big \}$ which are of different scales. Therefore, the stable and unstable manifold associated to the origin have dimension one and, thus, they are just trajectories in $\mathbb {R}^4$.

Since system (1.11) is autonomous, in order to find homoclinic connections, it is necessary that there exists a time parameterization of the stable and unstable invariant manifolds, denoted by

$$\begin{aligned} \big (u^{\star }(x), (u^{\star })'(x), v^{\star }(x), (v^{\star })'(x) \big ), \quad \star =\textrm{u},\textrm{s}\end{aligned}$$

(which also depend on the parameters $\varepsilon$ and $\gamma$), such that

$$\begin{aligned} \big (u^{\textrm{u}}(0), (u^{\textrm{u}})'(0), v^{\textrm{u}}(0), (v^{\textrm{u}})'(0) \big )= \big (u^{\textrm{s}}(0), (u^{\textrm{s}})'(0), v^{\textrm{s}}(0), (v^{\textrm{s}})'(0) \big ). \end{aligned}$$

In a general setting two curves do not intersect in a four dimensional space, however system (1.11) is reversible with respect to the involution

1.13 $$\begin{aligned} \Psi :(u,u',v,v')\rightarrow (u,-u',v,-v') \end{aligned}$$

whose symmetry plane is

1.14 $$\begin{aligned} \Pi =\{ (u,u',v,v') \in \mathbb {R}^4: \quad u'=0, \; v'=0\}. \end{aligned}$$

In other words, if $(u(x),u'(x),v(x),v'(x))$ is a solution of system (1.11), then the function defined by $\Psi (u(-x),u'(-x),v(-x),v'(-x))$ is also a solution. In particular

$$\begin{aligned} u^{\textrm{s}}(x)=u^\textrm{u}(-x), \qquad v^{\textrm{s}}(x) = v^{\textrm{u}}(-x) \end{aligned}$$

and therefore $u^{\textrm{s}}(0)= u^{\textrm{u}}(0)$ and $v^{\textrm{s}}(0) = v^{\textrm{u}}(0)$.

As a consequence, a homoclinic orbit exists if the unstable curve to (0, 0, 0, 0) as $x \rightarrow -\infty$ intersects the symmetry plane $\Pi$. Indeed, if such intersection occurs, then the unstable curve to (0, 0, 0, 0) as $x \rightarrow -\infty$ is reflected by the involution to the stable curve to (0, 0, 0, 0) as $x \rightarrow +\infty$.

It can be seen that the perturbed invariant manifolds can be approximated by the homoclinic orbit for the unperturbed problem (1.2),

$$ (u(x),u'(x),v(x),v'(x))=(u_0(x),u'_0(x), 0,0) $$

with $u_0$ given in (1.7). Then, we define the section

1.15 $$\begin{aligned} \Sigma =\{(u,u',v,v')\in {\mathbb {R}}^4: \quad u'=0\}. \end{aligned}$$

We observe that the homoclinic orbit $(u(x),u'(x))=(u_0(x),0)$ of the second-order system (1.2) with $u_0$ computed in (1.7), satisfies $u_0'(0)=0$ and it intersects transversally the section $\Sigma$ with $(v,v') = (0,0)$.

Next theorem gives an asymptotic formula for the distance between the stable and unstable manifolds of the origin in ${\mathbb {R}}^4$ at $\Sigma$.

#### Theorem 1.2

There exist two unique solutions $(u^\textrm{u}, v^\textrm{u})$ and $(u^\textrm{s}, v^\textrm{s})$ of system (1.11) such that $(u^\textrm{u})'(0)=(u^\textrm{s})'(0)=0$ and

$$\begin{aligned} \lim _{x\rightarrow -\infty }(u^\textrm{u}(x), v^\textrm{u}(x))=0,\qquad \lim _{x\rightarrow +\infty }(u^\textrm{s}(x), v^\textrm{s}(x))=0. \end{aligned}$$

Moreover, there exists a constant $\Theta \in {\mathbb {R}}$, $\Theta \ne 0$, such that

$$\begin{aligned} \begin{aligned} u^\textrm{u}(0)-u^\textrm{s}(0)&= 0\\ v^\textrm{u}(0)-v^\textrm{s}(0)&= 0\\ (v^\textrm{u})'(0)-(v^\textrm{s})'(0)&=-\frac{4\Theta }{\sqrt{|\gamma |}\varepsilon ^3}e^{-\frac{\pi }{\varepsilon }} \left( \sin \left( \frac{\alpha }{\varepsilon }\right) +{\mathcal {O}}\left( \frac{1}{|\log \varepsilon |}\right) \right) . \end{aligned} \end{aligned}$$

Theorem 1.1 is a direct consequence of Theorem 1.2.

#### Proof of Theorem 1.1

Since the system (1.11) is reversible it is enough to obtain a point in the unstable manifold which intersects the symmetry plane $\Pi$ in (1.14). Since

$$\begin{aligned}(u^\textrm{u}(0),(u^\textrm{u})'(0),v^\textrm{u}(0),(v^\textrm{u})'(0))\in \Sigma \end{aligned}$$

it is enough to look for values of $\varepsilon$ such that $(v^\textrm{u})'(0)=0$.

By reversibility,

$$\begin{aligned} (u^\textrm{u}(0),(u^\textrm{u})'(0),v^\textrm{u}(0),(v^\textrm{u})'(0))=(u^\textrm{s}(0),-(u^\textrm{s})'(0),v^\textrm{s}(0),-(v^\textrm{s})'(0)). \end{aligned}$$

and therefore

$$\begin{aligned} 2 (v^\textrm{u})'(0)=(v^\textrm{u})'(0)-(v^\textrm{s})'(0)=-\frac{4\Theta }{\sqrt{|\gamma |}\varepsilon ^3}e^{-\frac{\pi }{\varepsilon }} \left( \sin \left( \frac{\alpha }{\varepsilon }\right) +{\mathcal {O}}\left( \frac{1}{|\log \varepsilon |}\right) \right) . \end{aligned}$$

Since $\Theta \ne 0$, the values of $\varepsilon _n$ are found from roots of

$$\begin{aligned} \sin \left( \frac{\alpha }{\varepsilon }\right) +{\mathcal {O}}\left( \frac{1}{|\log \varepsilon |}\right) = 0, \end{aligned}$$

which yields (1.9).$$\square $$

The main steps in the proof of Theorem 1.2 are explained in Sect. 2. The proof of each step is deferred to Sects. 3–7 and Appendices A–C.

### Exponentially small splitting of separatrices

Theorem 1.2 fits into what is usually called exponentially small splitting of separatrices. This phenomenon occurs in dynamical systems which have a hyperbolic behavior whose invariant manifolds are exponentially close with respect to a small parameter of the system. Here we review the literature on the topic and explain the main tools to deal with the exponentially small phenomenon.

The exponentially small splitting of separatrices was first pointed out by Poincaré (see [47]) and nowadays it is well known that appear in many analytic models with multiple time scales and a conservative structure (Hamiltonian, volume preserving) or reversibility. The first rigorous analysis of this phenomenon was not achieved until the 1980’s in the seminal work by Lazutkin on the standard map [37], who proposed a scheme to prove the exponentially small transversality of the invariant manifolds of the saddle equilibrium point this map possesses. A full proof of this fact was obtained in 1999 by Gelfreich [24].

The approach proposed by Lazutkin (detailed below in this section) has been implemented in multiple settings in the past decades such as area preserving maps [17, 43, 44] and integrable Hamiltonian systems with a fast periodic or quasiperiodic forcing [8, 18, 25, 49]. Note that the approach is extremely sensitive on the analyticity properties of the model and therefore “implementing” it in different settings is, by no means, straightforward. Strongly related to the present paper are those dealing with volume preserving or Hamiltonian Hopf-zero bifurcations. This was first addressed in [5–7, 11, 12] and in [28], and has later been applied to the breakdown of breathers in the Klein-Gordon equation (which can be seen as an infinite dimensional Hopf-zero bifurcation) [30] and in the invariant manifolds of $L_3$ in the restricted planar 3 body problem [9, 10]. Note that the exponentially small splitting of separatrices phenomena can be analyzed by other methods such as the so-called continuous averaging method [53].

Let us explain the main steps of the approach proposed by Lazutkin applied to Hopf-zero bifurcations. Note first that the unperturbed separatrix is analytic in a complex strip centered at the real line. Then, in all the mentioned works and in the approach explained below, one makes the strong assumption that, at each of the boundary lines of the strip, the separatrix has only one singularity. Then, an asymptotic formula for the distance between the perturbed invariant manifolds can be obtained following these steps.

1. Choose coordinates which capture the slow-fast dynamics of the model so that it becomes a (fast) oscillator weakly coupled to an integrable system with a saddle point and a separatrix associated to it.
2. Prove the existence of the analytic continuation of suitable parametrizations of the perturbed invariant manifolds in appropriate complex domains. These domains contain a segment of the real line and intersect a neighborhood sufficiently close to the singularities of the separatrix.
3. Derive the inner equation, which gives the first order of the original system close to the singularities of the separatrix. This equation is independent of the perturbation parameter.
4. Study two special solutions of the inner equation which are approximations of the perturbed invariant manifolds near the singularities and provide an asymptotic formula for the difference between these two solutions of the inner equation.
5. By using complex matching techniques, compare the solutions of the inner equation with the parametrizations of the perturbed invariant manifolds.
6. Finally, prove that the dominant term of the difference between manifolds is given by the term obtained from the difference of the solutions of the inner equation.

This approach and all the aforementioned references rely on several hypotheses one has to assume on the model. In particular, as already said, one must assume that, at each of the boundary lines of its analyticity strip, the time-parameterization of the unperturbed separatrix has only one singularity. This assumption is rather strong and it is known to be non-generic (see [3, 27]). In particular, the model (1.1) with $\gamma \in \left( -\frac{1}{9},0\right)$ we consider in this paper does not satisfy this hypothesis since two singularities exist at each of these lines.

As far as the authors know, no proof of exponentially small splitting of separatrices for separatrices with multiple singularities with the same imaginary part existed until now. The reason is that to analytically extend the invariant manifolds to complex domains one needs to estimate quite sharply certain oscillatory integrals and this is not so straightforward when one has several singularities with the same imaginary part. In the present paper we propose a new approach which relies on considering “auxiliary orbits” of the model. The approach is rather flexible and we expect to be applicable to a wide set of models admitting *any number* of singularities with the same imaginary part (see Sect. 1.4 below).

Let us explain the main steps in the proof of Theorem 1.2, comparing them with the classical Lazutkin’s approach explained above. The singularities of the unperturbed separatrix closest to the real axis are those given in (1.8).

1. Choose coordinates which capture the slow-fast dynamics of the model. In the present paper the coordinates in (1.11) suffice. Note that this system possesses a first integral (see (1.12)).
2. Prove the existence of the analytic continuation of the time-parametrization of the perturbed unstable invariant manifolds in an appropriate complex domain (see (2.7)). This domain contains a segment of the real line and intersects a neighborhood sufficiently close to the singularities of the separatrix with negative real part (see (1.8)). Analogously, extend the perturbed stable invariant manifold up to the singularities with positive real part. This is done in Theorem 2.2.
3. Consider an auxiliary solution of (1.11) which belongs to the same level of the first integral and that can be defined in a lozenge shaped complex domain which contains a segment of the real line and domains $\varepsilon$-close to all four singularities of the unperturbed separatrix (see (2.10)). This is done in Theorem 2.3. Note that this solution does not belong to neither the stable nor the unstable invariant manifold. Instead of measuring the distance between the stable and unstable invariant manifolds at a given section, we will measure the distance between the unstable manifold and the auxiliary solution and between the auxiliary solution and the stable manifold.
4. Derive the inner equation (see (2.25)), which gives the first order of the original system close to the singularities of the separatrix. Note that the same inner equation appears close to all four singularities in (1.8).
5. Study two special solutions of the inner equation and provide an asymptotic formula for the difference between these two solutions of the inner equation. This is done in Theorem 2.8.
6. Close to the singularities with negative real part, by using complex matching techniques, compare the solutions of the inner equation with the parametrization of the perturbed unstable invariant manifold and the auxiliary solution (analogously close to the singularities with positive real part and the auxiliary solution and the parameterization of the stable invariant manifold). This is done in Theorem 2.10.
7. Prove that the dominant term of the difference between the unstable manifold and the auxiliary solution is given by the term obtained from the difference of the solutions of the inner equation close to the singularities with negative real part (analogously for the stable manifold and the auxiliary solution close to the rightmost singularities). This is done in Propositions 2.7 and 2.11. Joining the two asymptotic formulas provides the difference between the stable and unstable invariant manifolds.

### Further directions and applications

Although we have addressed a very particular model, the fourth-order equation (1.1), which is relevant for traveling waves of the modified Kawahara equation (1.6), the statement and proof of Theorem 1.2 can be extended to other dynamical systems with the saddle-center points.

One example where a sequence of homoclinic orbits appears in the singular perturbation theory was considered in [1]. The limiting second-order equation is given by

1.16 $$\begin{aligned} u'' - u + \frac{u^3}{1 + \gamma u^2} = 0, \end{aligned}$$

with a parameter $\gamma > 0$ and it appears as the standing wave reduction of the focusing nonlinear Schrödinger (NLS) equation with a saturation term. If $\gamma = 0$, the homoclinic orbit is given by $u_0(x) = \sqrt{2} \textrm{sech}(x)$ with the simple pole singularities along the imaginary axis at

$$ x = \frac{i \pi (2n + 1)}{2}, \quad n \in \mathbb {Z}. $$

However, for every $\gamma > 0$ it was proven in [1, Theorem 2.2] that the nearest singularities to the real line appear as a quadruplet in the complex plane. Hence, the numerical approximations in [1, Section 3] showed the existence of a countable sequence of true homoclinic orbits in the dynamical system in $\mathbb {R}^4$, where the limiting second-order equation (1.16) is perturbed by the fourth-order derivative term.

This example is rather striking since the term $u^3/(1+\gamma u^2)$ with $\gamma > 0$ does not change the number and types of the critical points in the dynamical system on the real line, but only change the number and types of singularities in the complex plane.

Another example appears in the cubic–quintic NLS equation

1.17 $$\begin{aligned} u'' - u + u^3 (1 + 3 \gamma u^2) = 0 \end{aligned}$$

with another parameter $\gamma \in \mathbb {R}$. The homoclinic orbit is given by

$$\begin{aligned} u_0(x) = \frac{2}{\sqrt{1 + \sqrt{1 + 16 \gamma } \cosh (2x)}}. \end{aligned}$$

The simple pole singularity for $\gamma = 0$ at $x = \frac{i\pi }{2}$ splits vertically along the imaginary axis for $\gamma > 0$ and horizontally for $\gamma < 0$ with a pair of the square root branch point singularities. In the latter case, we have a quadruplet of square root singularities in the complex plane which lead to a sequence of homoclinic orbit in the dynamical system in $\mathbb {R}^4$, where the second-order equation (1.17) is perturbed by the fourth-order derivative term.

For both models (1.16) and (1.17), the singularities in the complex plane are more complicated than poles and involve branching points, see [1].

The analytical proof of Theorem 1.2 can be extended from fourth-order dynamical systems to other finite-dimensional dynamical systems. It is nevertheless an open direction to extend the analysis to the infinite-dimensional dynamical systems such as the differential advance-delay equations. Such situations with the saddle-center points and the quadruplets of singularities in the complex plane are well-known in the context of traveling solitary waves in diatomic Fermi–Pasta–Ulam (FPU) systems [19, 41]. If the center manifold is still two-dimensional and the stable and unstable manifolds are infinite-dimensional, we conjecture that a similar sequence of true homoclinic orbits exist in the singular limit of the diatomic FPU system, in agreement with the numerical results in [22, 42, 54]. However, the proof of this conjecture is left for further studies.

## Details of the Proof

We devote this section to prove Theorem 1.2. First in Sect. 2.1 we provide analytic properties of the unperturbed solution (1.7). Then, in Sect. 2.2 we study the analytic continuation of the perturbed solutions in suitable complex domains and we also analyze the auxiliary solution. In Sect. 2.3 we give exponential upper bounds for the difference between two solutions for the stable and unstable invariant manifolds at a given transverse cross-section. To provide an asymptotic formula for this difference we analyze the first order of the perturbed solutions close to the singularities of the unperturbed solution. This is done in Sect. 2.4 by means of an inner equation and complex matching techniques. Finally, in Sect. 2.5 we obtain the asymptotic formula for the difference between two solutions for the stable and unstable invariant manifolds.

We will use the notation $'$ and $\partial _x$ to indicate the derivative with respect to *x*. In addition, when defining functional operators, we usually omit the dependence of some known functions such as $u_0$ on *x*.

### Properties of the unperturbed solution

The first step in the proof of Theorem 1.2 is to analyze the analytic properties of the unperturbed solution $u_0$ introduced in (1.7). This is contained in the following lemma, the proof of which can be found in Appendix A.

#### Lemma 2.1

For $\gamma \in (-\frac{1}{9},0)$, the function $u_0$ in (1.7) has the following properties:

- At the line $\Im x= \pi$ $u_0$ has exactly two singularities at
  2.1 $$\begin{aligned} x_\pm =\pm \alpha +\pi i, \quad \alpha =\cosh ^{-1}\frac{1}{\sqrt{1+9\gamma }} \end{aligned}$$
  and at $\Im x= -\pi$ $u_0$ has singularities at the conjugate points $\overline{x_\pm }$
- $u_0$ is real analytic in $\mathbb {C} \backslash \{x_\pm +i2k\pi , \overline{x_\pm }-i2k\pi \}_{k\in \mathbb {N}}$.
- In a neighborhood of $x_\pm$, $u_0$ satisfies
  $$\begin{aligned} u_0(x) = \frac{c_{\pm 1}}{x-x_\pm } + \mathcal {O}(1) \quad \text{ as } \;\; x \rightarrow x_\pm , \end{aligned}$$
  with
  2.2 $$\begin{aligned} c_{\pm 1}= \mp \frac{1}{\sqrt{|\gamma |}}. \end{aligned}$$
- The second derivative of $u_0$ has exactly eight zeros, $x_j^{\pm }$, $j=1,2,3,4$ with $|\Im x_j^{\pm }| \le \pi$ of the form
  $$ x_1^{\pm } = \pm i b, \qquad x_2^{\pm }= \pm a, \qquad x_3^{\pm }= \pm \textbf{a}+ i \pi , \qquad x_4^{\pm } = \pm \textbf{a}- i \pi $$
  with
  $$ b\in \left( \frac{\pi }{2}, \pi \right) , \qquad a > \alpha , \qquad \textbf{a} \in (0,\alpha ). $$

### The outer scale

The second step in the proof of Theorem 1.2 is to look for parameterizations of the one-dimensional stable and unstable invariant manifolds in the system (1.11). We parameterize them as solutions of equation (1.11) by fixing the initial condition at $\Sigma$ defined in (1.15).

We analyze the invariant manifolds by a perturbative approach close to $(u_0,0)$ where $u_0$ is the solution of (1.2) introduced in (1.7) that satisfies $u_0'(0) = 0$. To this end, we write

$$\begin{aligned} u=u_0+\xi ,\quad v=\eta , \end{aligned}$$

which yields the following system

2.3 $$\begin{aligned} \left\{ \begin{array}{l} {\mathcal {L}}_1\xi ={\mathcal {F}}_1 [\xi ,\eta ], \\ {\mathcal {L}}_2\eta ={\mathcal {F}}_2 [\xi ,\eta ], \end{array} \right. \end{aligned}$$

where the linear operators are defined by

2.4 $$\begin{aligned} \left\{ \begin{array}{l} {\mathcal {L}}_1 =-\partial _x^2+1-2u_0(x)-6\gamma u_0^2(x),\\ {\mathcal {L}}_2 =\partial _x^2+\frac{1}{\varepsilon ^2}, \end{array} \right. \end{aligned}$$

and

2.5 $$\begin{aligned} \left\{ \begin{array}{l} {\mathcal {F}}_1[\xi ,\eta ] =-\eta +(1+6\gamma u_0) \xi ^2 + 2 \gamma \xi ^3,\\ {\mathcal {F}}_2[\xi ,\eta ] =f'(u_0+\xi )\left( u_0+\xi +\eta -f(u_0+\xi )\right) +f''(u_0+\xi )(u_0'+\xi ')^2, \end{array} \right. \end{aligned}$$

with *f* defined in (1.10). Now, since

$$\begin{aligned} \eta '=u'''-u'+f'(u)u', \end{aligned}$$

the first integral (1.12) becomes

2.6 $$\begin{aligned} \widetilde{G}(\xi ,\xi ',\eta ,\eta ',x) =&\frac{1}{2} (1-\varepsilon ^2)\left[ (u_0')^2 + 2 u_0'\xi ' + (\xi ')^2 \right] - \frac{1}{2} \left[ u_0^2 - 2 u_0 \xi - \xi ^2 \right] + F(u_0+\xi )\nonumber \\&+\varepsilon ^2\Big [\left( u_0'+\xi '\right) \left( \eta '+u_0'+\xi '-f'(u_0+\xi )(u_0'+\xi ')\right) \nonumber \\&-\frac{1}{2} (\eta +u_0+\xi -f(u_0+\xi ))^2 \Big ], \end{aligned}$$

which is constant along solutions of (2.3).

The following theorem, whose proof is given in Sect. 3, provides two solutions of (2.3) which decay exponentially as $\Re x\rightarrow + \infty$ and $\Re x\rightarrow - \infty$ respectively. They correspond to the parameterizations of the invariant manifolds. Moreover, we prove that they can be analytically extended to the so-called outer domains defined as

**Fig. 2:**
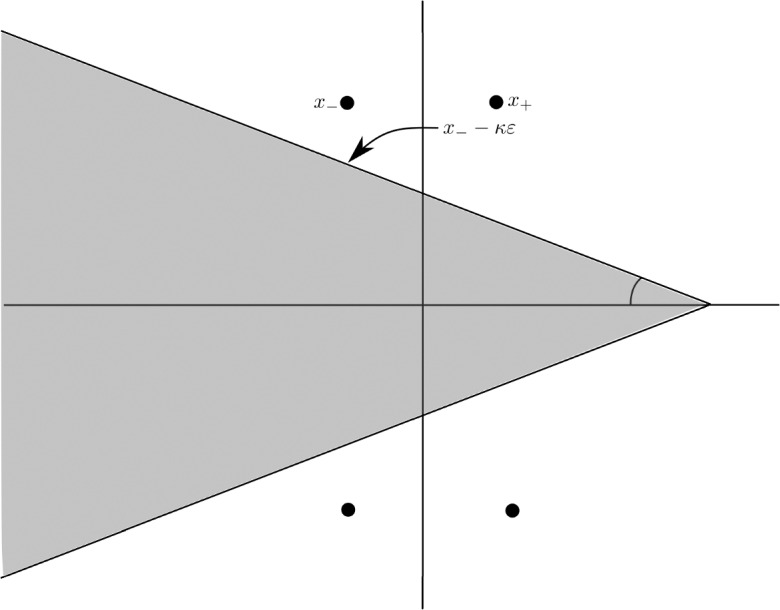
The outer domain $D^{\textrm{out},\textrm{u}}_{\kappa }$ introduced in (2.7)

2.7 $$\begin{aligned} \begin{aligned} D^{\textrm{out},\textrm{u}}_{\kappa }=&\,\left\{ x\in {\mathbb {C}}: \quad \left| {{\,\textrm{Im}\,}}(x)\right| {<} -\tan \theta {{\,\textrm{Re}\,}}(x-x_-)+ {{\,\textrm{Im}\,}}x_--\kappa \varepsilon \right\} ,\\ D^{\textrm{out},\textrm{s}}_{\kappa } =&\,\left\{ x\in {\mathbb {C}}: \quad \left| {{\,\textrm{Im}\,}}(x)\right| {<}\tan \theta {{\,\textrm{Re}\,}}(x-x_+) + {{\,\textrm{Im}\,}}x_+-\kappa \varepsilon \right\} , \end{aligned} \end{aligned}$$

where $0<\theta <\textrm{atan} \left( \frac{\pi }{3\alpha }\right)$, with $\alpha$ defined in (2.1), is a fixed angle independent of $\varepsilon$ and $\kappa \ge 1$ (see Fig. 2). Observe that $D^{\textrm{out},\star }_{\kappa }$, $\star =\textrm{u},\textrm{s}$, reach domains at a $\kappa \varepsilon$–distance of the singularities $x=x_-$ and $x=x_+$ of $u_0$ respectively.

#### Theorem 2.2

Fix $0<\theta <\textrm{atan} \left( \frac{\pi }{3\alpha }\right)$. There exists $\kappa _0, \varepsilon _0>0$, such that, if $\varepsilon \in (0, \varepsilon _0)$ and $\kappa > \kappa _0$, then there exist real-analytic functions $(\xi ^\star ,\eta ^\star )$, $\star =\textrm{u},\textrm{s}$, defined in the domain $D^{\textrm{out},\star }_{\kappa }$ which are solutions of (2.3) satisfying

$$\begin{aligned} \begin{aligned} \lim _{\Re x\rightarrow -\infty }(\xi ^{\textrm{u}},\eta ^{\textrm{u}})=(0,0),\qquad \lim _{\Re x\rightarrow \infty }(\xi ^{\textrm{s}},\eta ^{\textrm{s}})=(0,0) \end{aligned} \end{aligned}$$

and

$$\begin{aligned} \partial _x \xi ^\star (0)=0,\qquad \widetilde{G}(\xi ^\star ,\partial _x \xi ^\star ,\eta ^\star ,\partial _x \eta ^\star ,x)=0, \end{aligned}$$

where $\widetilde{G}$ is the first integral introduced in (2.6).

Moreover, there exists $M_1>0$, depending only on $\theta ,\kappa _0,\varepsilon _0$, such that $\xi ^\star$ and $\eta ^\star$, $\star =\textrm{u},\textrm{s}$, satisfy the following estimates.

- For $$x\in D^{\textrm{out},\star }_{\kappa }\cap \{|{{\,\textrm{Re}\,}}(y)|\ge 2\alpha \}$$,
  $$\begin{aligned} |\xi ^\star (x)|\le \, M_1\varepsilon ^2 e^{-|\Re x|},\qquad |\eta ^\star (x)|\le \, M_1\varepsilon ^2 e^{-|\Re x|} \end{aligned}$$
  and
  $$\begin{aligned} |\partial _x\xi ^\star (x)|\le \, M_1\varepsilon ^2 e^{-|\Re x|},\qquad |\partial _x\eta ^\star (x)|\le \, M_1\varepsilon ^2 e^{-|\Re x|}. \end{aligned}$$
- For $$x\in D^{\textrm{out},\star }_{\kappa }\cap \{|{{\,\textrm{Re}\,}}(y)|\le 2\alpha \}$$,
  $$\begin{aligned} \begin{aligned} |\xi ^\star (x)|&\le \, \frac{M_1\varepsilon ^2}{|x-x_-|^3|x-\overline{x}_-|^3|x-x_+|^3|x-\overline{x}_+|^3},\\ |\eta ^\star (x)|&\le \, \frac{M_1\varepsilon ^2}{|x-x_-|^5|x-\overline{x}_-|^5|x-x_+|^5|x-\overline{x}_+|^5},\\ |\partial _x\xi ^\star (x)|&\le \, \frac{M_1\varepsilon ^2}{|x-x_-|^4|x-\overline{x}_-|^4|x-x_+|^4|x-\overline{x}_+|^4},\\ |\partial _x\eta ^\star (x)|&\le \, \frac{M_1\varepsilon }{|x-x_-|^5|x-\overline{x}_-|^5|x-x_+|^5|x-\overline{x}_+|^5}. \end{aligned} \end{aligned}$$

Finally,

$$\begin{aligned} \xi ^\textrm{s}(x)=\xi ^\textrm{u}(-x), \qquad \eta ^\textrm{s}(x) = \eta ^\textrm{u}(-x) \end{aligned}$$

or, in other words, the unstable curve is reflected by the involution $\Psi$ in (1.13) to the stable one.

To prove Theorem 1.2, we analyze the difference

2.8 $$\begin{aligned} \Delta =(\Delta \xi ,\Delta \eta )=(\xi ^{\textrm{u}}-\xi ^{\textrm{s}}, \eta ^{\textrm{u}}-\eta ^{\textrm{s}}). \end{aligned}$$

However, since its difference is exponentially small, to obtain an asymptotic formula, we would need to analyze this difference in $\varepsilon$-neighborhoods of the singularities $x=x_\pm$. Note that Theorem 2.2 does not provide the analytic continuation of $(\xi ^{\textrm{s}}, \eta ^{\textrm{s}})$ to points $\kappa \varepsilon$-close to $x_-$ (and same happens for $(\xi ^{\textrm{u}}, \eta ^{\textrm{u}})$ and $x_+$).

Instead of performing the analytic extension of the invariant manifolds in the $\kappa \varepsilon$-neighborhood of the points $x_{\pm }$, we rely on auxiliary functions $(\xi ^\textrm{aux}, \eta ^\textrm{aux})$. These functions will be solutions of the same equation (2.3) and will also belong to the same energy level with respect to $\widetilde{G}$ as $(\xi ^{\textrm{u},\textrm{s}}, \partial _x \xi ^{\textrm{u},\textrm{s}}, \eta ^{\textrm{u},\textrm{s}}, \partial _x \eta ^{\textrm{u},\textrm{s}})$. Then, the analysis of the difference (2.8) will be deduced by the differences

2.9 $$\begin{aligned} \begin{aligned} \Delta ^{\textrm{u}}&=(\Delta \xi ^{\textrm{u}},\Delta \eta ^{\textrm{u}})=(\xi ^{\textrm{u}}-\xi ^\textrm{aux}, \eta ^{\textrm{u}}-\eta ^\textrm{aux}),\\ \Delta ^{\textrm{s}}&=(\Delta \xi ^{\textrm{s}},\Delta \eta ^{\textrm{s}})=(\xi ^\textrm{aux}-\xi ^{\textrm{s}}, \eta ^\textrm{aux}-\eta ^{\textrm{s}}). \end{aligned} \end{aligned}$$

The following theorem, whose proof is given in Sect. 4, provides the existence of the functions $(\xi ^\textrm{aux}, \eta ^\textrm{aux})$ in the domain

**Fig. 3:**
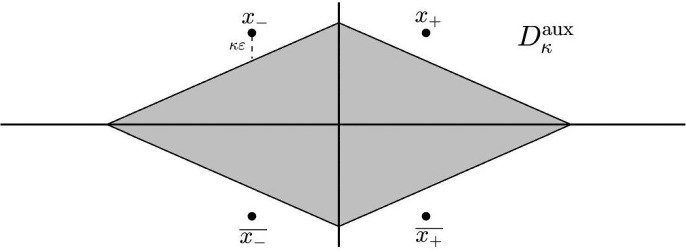
The auxiliary domain $D^{\textrm{aux}}_{\kappa }$ introduced in (2.10)

2.10 $$\begin{aligned} \begin{aligned} D^\textrm{aux}_\kappa =&\left\{ x\in {\mathbb {C}}: \quad \left| {{\,\textrm{Im}\,}}(x)\right| {<} \tan \theta {{\,\textrm{Re}\,}}(x-x_-)+\pi -\kappa \varepsilon \right\} \\&\cap \left\{ x\in {\mathbb {C}}: \quad \left| {{\,\textrm{Im}\,}}(x)\right| {<} -\tan \theta {{\,\textrm{Re}\,}}(x-x_+)+\pi -\kappa \varepsilon \right\} \end{aligned} \end{aligned}$$

with $\kappa ,\theta >0$. The domain is shown in Fig. 3.

#### Theorem 2.3

Let $0<\theta <\textrm{arctan}\left( \frac{\pi }{\alpha } \right)$. There exists $\kappa _0, \varepsilon _0>0$, such that, if $\varepsilon \in (0, \varepsilon _0)$ and $\kappa > \kappa _0$, then there exist real-analytic functions $(\xi ^\textrm{aux},\eta ^\textrm{aux})$ defined in the domain $D^\textrm{aux}_\kappa$ which are a solution of (2.3) and satisfy

$$\begin{aligned} \partial _x \xi ^\textrm{aux}(0)=0\qquad \text {and}\qquad \widetilde{G}(\xi ^\textrm{aux},\partial \xi ^\textrm{aux},\eta ^\textrm{aux},\partial _x \eta ^\textrm{aux},x)=0 \end{aligned}$$

where $\widetilde{G}$ is the first integral introduced in (2.6).

Moreover, there exists $M_2$, depending on $\theta ,\kappa _0,\varepsilon _0$ such that, for $x\in D^\textrm{aux}_\kappa$,

$$\begin{aligned} \begin{aligned} |\xi ^\textrm{aux}(x)|&\le \, \frac{M_2\varepsilon ^2}{|x-x_-|^3|x-\overline{x}_-|^3|x-x_+|^3|x-\overline{x}_+|^3}\\ |\eta ^\textrm{aux}(x)|&\le \, \frac{M_2\varepsilon ^2}{|x-x_-|^5|x-\overline{x}_-|^5|x-x_+|^5|x-\overline{x}_+|^5}\\ |\partial _x\xi ^\textrm{aux}(x)|&\le \, \frac{M_2\varepsilon ^2}{|x-x_-|^4|x-\overline{x}_-|^4|x-x_+|^4|x-\overline{x}_+|^4}\\ |\partial _x\eta ^\textrm{aux}(x)|&\le \, \frac{M_2\varepsilon }{|x-x_-|^5|x-\overline{x}_-|^5|x-x_+|^5|x-\overline{x}_+|^5} \end{aligned} \end{aligned}$$

In addition $$(\xi ^\textrm{aux}(x),\eta ^\textrm{aux}(x))= (\xi ^\textrm{aux}(-x),\eta ^\textrm{aux}(-x)$$.

### Exponentially small estimates

The next step in the proof of Theorem 1.2 is to analyze the differences $\Delta ^{\textrm{u}}$, $\Delta ^{\textrm{s}}$ defined in (2.9). Since $(\xi ^\star ,\eta ^\star )$, $\star =\textrm{u},\textrm{s},\textrm{aux}$ are all solutions of (2.3), we can conclude in the following lemma that the differences $\Delta ^\star$ are solutions of a linear system in the following domains

**Fig. 4:**
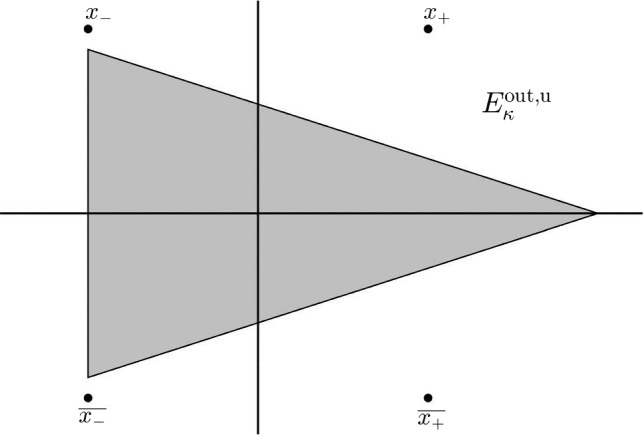
The intersection domain $E^{\textrm{out},\textrm{u}}_{\kappa }$ introduced in (2.11)

2.11 $$\begin{aligned} \begin{aligned} E^{\textrm{out},\textrm{u}}_{\kappa }=&\,\left\{ x\in {\mathbb {C}}: \quad \left| {{\,\textrm{Im}\,}}(x)\right| {<} -\tan \theta {{\,\textrm{Re}\,}}(x-x_-)+ {{\,\textrm{Im}\,}}x_--\kappa \varepsilon , \Re x{>}\Re x_- \right\} ,\qquad \\ E^{\textrm{out},\textrm{s}}_{\kappa } =&\,\left\{ x\in {\mathbb {C}}: \quad \left| {{\,\textrm{Im}\,}}(x)\right| {<} \tan \theta {{\,\textrm{Re}\,}}(x-x_+) + {{\,\textrm{Im}\,}}x_+-\kappa \varepsilon , \Re x{<} \Re x_- \right\} ,\qquad \end{aligned} \end{aligned}$$

(see Fig. 4). Note that these domains, with $\theta$ such that $0<\theta < \textrm{atan} \left( \frac{\pi }{3\alpha }\right)$, satisfy $E^{\textrm{out},\star }_{\kappa }\subset D^{\textrm{out},\star }_{\kappa }\cap D^{\textrm{aux}}_{\kappa }$, $\star =\textrm{u},\textrm{s}$.

#### Lemma 2.4

The functions $\Delta ^\star =(\Delta \xi ^\star ,\Delta \eta ^\star )$, $\star =\textrm{u},\textrm{s}$, in (2.9) are defined in the domains $E^{\textrm{out},\star }_{\kappa }$ in (2.11) and are solutions of the linear system

2.12 $$\begin{aligned} \left\{ \begin{array}{l} {\mathcal {L}}_1\Delta \xi ={\mathcal {N}}_1[\Delta \xi ,\Delta \eta ], \\ {\mathcal {L}}_2\Delta \eta ={\mathcal {N}}_2[\Delta \xi ,\Delta \xi ',\Delta \eta ], \end{array} \right. \end{aligned}$$

where

2.13 $$\begin{aligned} \left\{ \begin{array}{l} {\mathcal {N}}_1[\Delta \xi ,\Delta \eta ](x) =-\Delta \eta (x)+a(x)\Delta \xi (x),\\ {\mathcal {N}}_2[\Delta \xi ,\Delta \xi ',\Delta \eta ](x) = b(x)\Delta \xi (x)+c(x)\Delta \xi '+d(x)\Delta \eta (x), \end{array} \right. \end{aligned}$$

for some functions *a*, *b*, *c* and *d*, which satisfy that, for $x\in E^{\textrm{out},\star }_{\kappa }$,

$$\begin{aligned} \begin{aligned} |a(x)|&\le \, \frac{M_3\varepsilon ^2}{|x-x_-|^4|x-\overline{x}_-|^4|x-x_+|^4|x-\overline{x}_+|^4},\\ |b(x)|&\le \, \frac{M_3}{|x-x_-|^4|x-\overline{x}_-|^4|x-x_+|^4|x-\overline{x}_+|^4},\\ |c(x)|&\le \, \frac{M_3}{|x-x_-|^3|x-\overline{x}_-|^3|x-x_+|^3|x-\overline{x}_+|^3},\\ |d(x)|&\le \, \frac{M_3}{|x-x_-|^2|x-\overline{x}_-|^2|x-x_+|^2|x-\overline{x}_+|^2}, \end{aligned} \end{aligned}$$

for some constant $M_3$ independent of $\varepsilon$ and $\kappa$.

To obtain the exponentially small estimates for the differences $\Delta ^{\star }$ ($\star = \textrm{u},\textrm{s}$), we use the existence of the first integral $\widetilde{G}(\xi ,\xi ',\eta ,\eta ',x)$. The first integral gives us an extra relation for the components of the difference $\Delta ^{\star }$, which allows us to get rid of analyzing $\Delta \xi ^{\star }$.

The following lemma is straightforward taking into account Lemma 2.1 and Theorems 2.2 and 2.3.

#### Lemma 2.5

The functions $\Delta ^\star =(\Delta \xi ^\star ,\Delta \eta ^\star )$, $\star = \textrm{u},\textrm{s}$, defined in (2.9) satisfy

$$\begin{aligned} \left( -u''_0(x)+m(x)\right) \Delta \xi +\left( u'_0(x)+n(x)\right) \Delta \xi '+p(x)\Delta \eta +q(x)\Delta \eta '=0 \end{aligned}$$

for some functions *m*, *n*, *p* and *q*, which satisfy that, for $x\in E^{\textrm{out},\star }_{\kappa }$,

$$\begin{aligned} \begin{aligned} |m(x)|&\le \, \frac{M_4\varepsilon ^2}{|x-x_-|^5|x-\overline{x}_-|^5|x-x_+|^5|x-\overline{x}_+|^5},\\ |n(x)|&\le \, \frac{M_4\varepsilon ^2}{|x-x_-|^4|x-\overline{x}_-|^4|x-x_+|^4|x-\overline{x}_+|^4},\\ |p(x)|&\le \, \frac{M_4\varepsilon ^2}{|x-x_-|^3|x-\overline{x}_-|^3|x-x_+|^3|x-\overline{x}_+|^3},\\ |q(x)|&\le \, \frac{M_4\varepsilon ^2}{|x-x_-|^2|x-\overline{x}_-|^2|x-x_+|^2|x-\overline{x}_+|^2}, \end{aligned} \end{aligned}$$

with $M_4>0$ a constant independent of $\varepsilon$ and $\kappa$.

By using Lemma 2.5, we reduce the system of two second-order equations (2.12) to a third-order system imposed on $\Delta \zeta =\Delta \xi '$, $\Delta \eta$ and $\Delta \eta '$. The following lemma is obtained directly from Lemmas 2.4 and 2.5.

#### Lemma 2.6

The functions $\Delta \zeta ^\star =\partial _x \Delta {\xi ^\star }$, $\Delta \eta ^\star$, $\star =\textrm{u},\textrm{s}$, are defined in $E^{\textrm{out},\star }$ in (2.11) and are solutions of the linear equation

2.14 $$\begin{aligned} \left\{ \begin{array}{l} \widehat{\mathcal {L}}_1\Delta \zeta =\widehat{\mathcal {N}}_1[\Delta \zeta ,\Delta \eta , \Delta \eta '],\\ {\mathcal {L}}_2\Delta \eta =\widehat{\mathcal {N}}_2[\Delta \zeta ,\Delta \eta ,\Delta \eta '], \end{array} \right. \end{aligned}$$

where

2.15 $$\begin{aligned} \widehat{\mathcal {L}}_1=-\partial _x+\frac{u_0'''}{u_0''}, \end{aligned}$$

and

$$\begin{aligned} \left\{ \begin{array}{l} \widehat{\mathcal {N}}_1[\Delta \zeta ,\Delta \eta ,\Delta \eta '] =-\Delta \eta +\widehat{r}(x)\Delta \zeta +\widehat{s}(x)\Delta \eta +\widehat{t}(x)\Delta \eta ', \vspace{0.2cm}\\ \widehat{\mathcal {N}}_2[\Delta \zeta ,\Delta \eta ,\Delta \eta '] =\widehat{c}(x)\Delta \zeta +\widehat{d}(x)\Delta \eta +\widehat{e}(x)\Delta \eta ', \end{array} \right. \end{aligned}$$

for some functions $\widehat{r}$, $\widehat{s}$, $\widehat{t}$, $\widehat{c}$, $\widehat{d}$ and $\widehat{e}$, which satisfy that, for $x\in E^{\textrm{out},\star }_{\kappa }$,

$$\begin{aligned} \begin{aligned} |\widehat{r}(x)|&\le \, \frac{M_5\varepsilon ^2}{|x-x_-|^3|x-\overline{x}_-|^3|x-x_+|^3|x-\overline{x}_+|^3},\\ |\widehat{s}(x)|&\le \, \frac{M_5\varepsilon ^2}{|x-x_-|^2|x-\overline{x}_-|^2|x-x_+|^2|x-\overline{x}_+|^2},\\ |\widehat{t}(x)|&\le \, \frac{M_5\varepsilon ^2}{|x-x_-||x-\overline{x}_-||x-x_+||x-\overline{x}_+|},\\ |\widehat{c}(x)|&\le \, \frac{M_5}{|x-x_-|^3|x-\overline{x}_-|^3|x-x_+|^3|x-\overline{x}_+|^3},\\ |\widehat{d}(x)|&\le \, \frac{M_5}{|x-x_-|^2|x-\overline{x}_-|^2|x-x_+|^2|x-\overline{x}_+|^2},\\ |\widehat{e}(x)|&\le \, \frac{M_5\varepsilon ^2}{|x-x_-|^3|x-\overline{x}_-|^3|x-x_+|^3|x-\overline{x}_+|^3}, \end{aligned} \end{aligned}$$

with $M_5$ a constant independent of $\varepsilon$ and $\kappa$.

By using Lemma 2.6, we provide an asymptotic formula for $\Delta ^\star$ at $x=0$. Note that, by Theorem 2.2 and 2.3, $\Delta \zeta ^\star (0)= \partial _x \Delta {\xi }^\star (0)=0$ (and that $\Delta \xi (0)$ can be obtained by Lemma 2.5 once the other components are known). Therefore, in order to prove Theorem 1.2, it is sufficient to look for an asymptotic formula for $\Delta \eta ^\star (0)$ and $\partial _x \Delta {\eta ^\star }(0)$.

Assume for a moment that $\Delta \eta ^\star$ satisfy

$$\begin{aligned} {\mathcal {L}}_2\Delta \eta =0 \end{aligned}$$

(that is, assume that $\widehat{c}= \widehat{d}=\widehat{e}=0)$. Then, $\Delta \eta ^\star$ would be of the form

2.16 $$\begin{aligned} \Delta \eta ^\star (x)=C_1^\star e^{\frac{ix}{\varepsilon }}+C_2^\star e^{-\frac{ix}{\varepsilon }}. \end{aligned}$$

We introduce

2.17 $$\begin{aligned} \rho _{-}= x_--i\kappa \varepsilon \quad \text { and }\quad {\rho _{+}}=x_+-i\kappa \varepsilon \end{aligned}$$

with $x_{\pm }= \pm \alpha + \pi i$ and $\alpha$ defined in Lemma 2.1. We observe that, by Theorems 2.2 and Theorem 2.3, $\Delta \eta ^\textrm{u}$ is defined at $\rho _{-}, \overline{\rho _{-}}$ and $\Delta \eta ^\textrm{s}$ is defined at $\rho _{+}$, $\overline{\rho _{+}}$. Evaluating $\Delta \eta ^\textrm{u}$ in (2.16) at $x=\rho _{-}$ and $x=\overline{\rho _{-}}$, using that $e^{\frac{i\rho _{-}}{\varepsilon }}$ and $e^{-\frac{i\overline{\rho _{-}}}{\varepsilon }}$ are of size $e^{-\frac{\pi }{\varepsilon }}$, one obtains that $C_1^{\textrm{u}}$ and $C_2^{\textrm{u}}$ must satisfy

2.18 $$\begin{aligned} C_1^{\textrm{u}}=\Delta \eta ^{\textrm{u}}(\overline{\rho _{-}})e^{-\frac{i\overline{\rho _{-}}}{\varepsilon }}+\text {h.o.t.} \qquad \text {and}\qquad C_2^{\textrm{u}}=\Delta \eta ^{\textrm{u}}(\rho _{-})e^{\frac{i{\rho _{-}}}{\varepsilon }}+\text {h.o.t.}. \end{aligned}$$

An analogous formula follows for $C_{1,2}^{\textrm{s}}$ changing $\rho _{-}$ by $\rho _{+}$.

Now, the equation for $\Delta \eta ^\star$, $\star =\textrm{u},\textrm{s}$, in (2.12) has a right hand side (2.13) with nonzero $\widehat{c}$, $\widehat{d}$, $\widehat{e}$ and therefore one has to proceed more carefully than in the arguments above. The following proposition gives the needed result.

#### Proposition 2.7

The functions $\Delta \eta ^\star$, $\star =\textrm{u},\textrm{s}$, introduced in (2.9) are defined in $E^{\textrm{out},\star }$ given by (2.11) and are of the form

2.19 $$\begin{aligned} \Delta \eta ^\star (x)=C_1^\star e^{\frac{ix}{\varepsilon }}+C_2^\star e^{-\frac{ix}{\varepsilon }}+{\mathcal {R}}^\star (x) \end{aligned}$$

where

- The constants $C_1^\star$ and $C_2^\star$ satisfy
  2.20 $$\begin{aligned} \begin{aligned} \Delta \eta ^{\textrm{u}}(\rho _{-})&=C_1^{\textrm{u}} e^{\frac{i\rho _{-}}{\varepsilon }}+C_2^{\textrm{u}} e^{-\frac{i\rho _{-}}{\varepsilon }}\\ \Delta \eta ^{\textrm{u}}(\overline{\rho _{-}})&=C_1^{\textrm{u}} e^{\frac{i\overline{\rho _{-}}}{\varepsilon }}+C_2^{\textrm{u}} e^{-\frac{i\overline{\rho _{-}}}{\varepsilon }}\\ \Delta \eta ^{\textrm{s}}(\rho _{+})&=C_1^{\textrm{s}} e^{\frac{i\rho _{+}}{\varepsilon }}+C_2^{\textrm{s}} e^{-\frac{i\rho _{+}}{\varepsilon }}\\ \Delta \eta ^{\textrm{s}}(\overline{\rho _{+}})&=C_1^{\textrm{s}} e^{\frac{i\overline{\rho _{+}}}{\varepsilon }}+C_2^{\textrm{s}} e^{-\frac{i\overline{\rho _{+}}}{\varepsilon }}. \end{aligned} \end{aligned}$$
- The functions ${\mathcal {R}}^\star$ satisfy that
  2.21 $$\begin{aligned} {\mathcal {R}}^{\textrm{u}}(\rho _{-})=0, \qquad {\mathcal {R}}^{\textrm{u}}(\overline{\rho _{-}})=0, \qquad {\mathcal {R}}^{\textrm{s}}(\rho _{+})=0,\qquad {\mathcal {R}}^{\textrm{s}}(\overline{\rho _{+}})=0, \end{aligned}$$
  and that, for $x\in E^{\textrm{out},\star }_{\kappa }$,
  2.22 $$\begin{aligned} \begin{aligned} \left| {\mathcal {R}}^\star (x)\right|&\le \frac{M_6}{\kappa }e^{\frac{1}{\varepsilon }|\Im x|}\left( |C_1^{\textrm{u}}|+|C_2^{\textrm{u}}|\right) \\ \left| \partial _x{\mathcal {R}}^\star (x)\right|&\le \frac{M_6}{\varepsilon \kappa }e^{\frac{1}{\varepsilon }|\Im x|}\left( |C_1^{\textrm{u}}|+|C_2^{\textrm{u}}|\right) , \end{aligned} \end{aligned}$$
  for some constant independent $M_6>0$ independent of $\varepsilon$ and $\kappa$.

Note that the properties of $C_j^\star$ are a direct consequence of evaluating (2.19) at $x=\rho ^\pm$ and $x=\overline{\rho ^\pm }$ and the properties of ${\mathcal {R}}^\star$. That is, to prove Proposition 2.7 boils down to prove the properties stated for the functions ${\mathcal {R}}^\star$. This is done in Sect. 7.

By Proposition 2.7, proceeding as for (2.16), we have that indeed, $C_{1,2}^{\textrm{u}}$ is of the form in (2.18) and analogous formula are also true for $C_{1,2}^{\textrm{s}}$. As a consequence, of this analysis and using also that, by Theorems 2.2 and 2.3

$$ |\Delta \eta ^{*} (\rho _\pm )| ,\, |\Delta \eta ^* (\overline{\rho _{\pm }}) |\le M \frac{1}{\kappa ^5\varepsilon ^3} , $$

we have that

$$ |C_{1,2}^\star |\le M \frac{1}{\varepsilon ^{3}} e^{-\frac{\pi }{\varepsilon }}. $$

However, in order to prove the asymptotic formula in Theorem 1.2, we need to perform a more accurate analysis of the functions $\eta ^\star$ (and $\xi ^\star$) around the points $\rho _\pm$ and $\overline{\rho _\pm }$. This is done in the following subsections by means of the inner equation (Theorem 2.8) and complex matching techniques (Theorem 2.10).

### The inner scale

We perform the change of coordinates to the inner variables. We consider the new variables

2.23 $$\begin{aligned} z=\varepsilon ^{-1}(x-x_{\pm }) \end{aligned}$$

and, recalling the definition of $c_{\pm 1}$ in (2.2), we define the functions

2.24 $$\begin{aligned} \phi (z)=\frac{\varepsilon }{c_{\pm 1}}\xi (x_\pm +\varepsilon z),\qquad \psi (z)=\frac{\varepsilon ^3}{c_{\pm 1}}\eta (x_\pm +\varepsilon z). \end{aligned}$$

Recall that $\gamma <0$ and therefore $c_{\pm 1}^2\gamma =-1$. Applying the change of coordinates to equation (2.3) and letting $\varepsilon \rightarrow 0$ we obtain the limiting inner equation,

2.25 $$\begin{aligned} \left\{ \begin{array}{l} {\mathcal {L}}_1^\textrm{in}\phi =\mathcal {J}^{\textrm{in}}_1[\phi ,\psi ], \\ {\mathcal {L}}_2^\textrm{in}\psi =\mathcal {J}^{\textrm{in}}_2[\phi ,\psi ], \end{array} \right. \end{aligned}$$

with

2.26 $$\begin{aligned} \left\{ \begin{array}{l} {\mathcal {L}}_1^\textrm{in}=-\partial _z^2+\frac{6}{z^2},\\ {\mathcal {L}}_2^\textrm{in}=\partial _z^2+1, \end{array} \right. \end{aligned}$$

and

2.27 $$\begin{aligned} \left\{ \begin{array}{l} \mathcal {J}^{\textrm{in}}_1[\phi ,\psi ] =-\psi -\frac{6}{z}\phi ^2-2\phi ^3,\\ \mathcal {J}^{\textrm{in}}_2[\phi ,\psi ] =-6\left( \frac{1}{z}+\phi \right) ^2\left( \psi +2\left( \frac{1}{z}+\phi \right) ^3\right) -12\left( \frac{1}{z}+\phi \right) \left( -\frac{1}{z^2}+\partial _z\phi \right) ^2. \end{array} \right. \nonumber \\ \end{aligned}$$

This equation is reversible with respect to the symmetry

2.28 $$\begin{aligned} (\phi ,\psi ) \rightarrow (-\phi ,-\psi ),\quad z\rightarrow -z. \end{aligned}$$

**Fig. 5:**
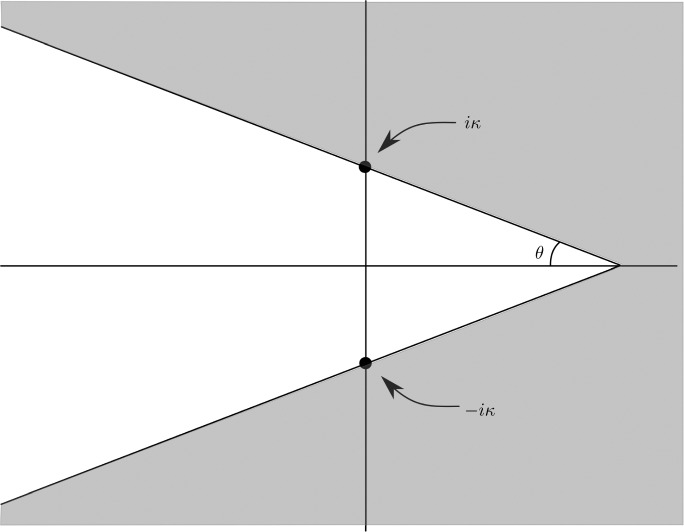
The inner domain $D^{\textrm{u},\textrm{in}}_{\theta ,\kappa }$ introduced in (2.29)

We analyze this equation in the *inner domains* (see Fig. 5)

2.29 $$\begin{aligned} \begin{array}{l} {\mathcal {D}}^{\textrm{u},\textrm{in}}_{\theta ,\kappa }=\{z\in {\mathbb {C}}: \quad |\Im (z)|> \tan \theta \Re (z)+\kappa \},\vspace{0.2cm}\\ {\mathcal {D}}^{\textrm{s},\textrm{in}}_{\theta ,\kappa }=\{z\in {\mathbb {C}}: \quad -z\in {\mathcal {D}}^{\textrm{u},\textrm{in}}_{\theta ,\kappa } \}, \end{array} \end{aligned}$$

for $$0<\theta <\pi /2$$ and $$\kappa >0$$.

The following theorem, which is proved in Sect. 5, provides an asymptotic formula for the difference between the two solutions of the inner equation.

#### Theorem 2.8

Let $0<\theta <\frac{\pi }{2}$ be fixed. There exists $\kappa _0\ge 1$ big enough such that, for each $\kappa \ge \kappa _0$,

1. Equation (2.25) has two real-analytic solutions $(\phi ^{0,\star }, \psi ^{0,\star }): {\mathcal {D}}^{\star ,\textrm{in}}_{\theta ,\kappa }\rightarrow {\mathbb {C}}^2$, $\star =\textrm{u},\textrm{s}$, which, for every $z\in {\mathcal {D}}^{\star ,\textrm{in}}_{\theta ,\kappa }$, satisfy
  $$\begin{aligned} \left| \phi ^{0,\star }(z) \right| \le \dfrac{M_7}{|z|^3}, \quad \left| \psi ^{0,\star } (z)\right| \le \dfrac{M_7}{|z|^5}, \end{aligned}$$
  for some $M_7>0$ independent of $\kappa$. Moreover, they satisfy that, for $z\in {\mathcal {D}}^{\textrm{u},\textrm{in}}_{\theta ,\kappa }$,
  2.30 $$\begin{aligned} (\phi ^{0,\textrm{u}}(z),\psi ^{0,\textrm{u}}(z))=(-\phi ^{0,\textrm{s}}(-z),-\psi ^{0,\textrm{s}}(-z)). \end{aligned}$$
2. The differences $\Delta \phi ^0(z)= \phi ^{0,\textrm{u}}(z)-\phi ^{0,\textrm{s}}(z)$, $\Delta \psi ^0(z)= \psi ^{0,\textrm{u}}(z)-\psi ^{0,\textrm{s}}(z)$ are given by
  2.31 $$\begin{aligned} \begin{aligned} \Delta \phi ^0(z)&=\Theta e^{-i z}\left( -1+\chi _1(z)\right) \\ \Delta \psi ^0(z)&=\Theta e^{-i z}\left( 1+ \chi _2(z)\right) \\ \partial _z\Delta \phi ^0(z)&=-i\Theta e^{-i z}\left( -1+\widehat{\chi }_1(z)\right) \\ \partial _z\Delta \psi ^0(z)&=-i\Theta e^{-i z}\left( 1+ \widehat{\chi }_2(z)\right) \end{aligned} \end{aligned}$$
  for $z\in \mathcal {R}^{\textrm{in}}_{\theta ,\kappa }= {\mathcal {D}}^{\textrm{u},\textrm{in}}_{\theta ,\kappa }\cap {\mathcal {D}}^{\textrm{s},\textrm{in}}_{\theta ,\kappa }\cap \{z: i{\mathbb {R}}, \Im z<0\}$, where $\Theta \in {\mathbb {R}}$ is a constant, and $\chi _1$, $\chi _2$, $\widehat{\chi }_1$, $\widehat{\chi }_2$ are analytic in *z* and satisfy that, for $z\in \mathcal {R}^{\textrm{in}}_{\theta ,\kappa }$,
  $$\begin{aligned} |\chi _1(z)|\le \dfrac{M_8}{|z|}, \quad |\chi _2(z)|\le \dfrac{M_8}{|z|},\quad |\widehat{\chi }_1(z)|\le \dfrac{M_8}{|z|} \quad |\widehat{\chi }_2(z)|\le \dfrac{M_8}{|z|},\end{aligned}$$
  for some $M_8>0$ independent of $\kappa$.
3. The constant $\Theta$ satisfies $\Theta \ne 0$ if and only if there exists $z_0\in \mathcal {R}^{\textrm{in}}_{\theta ,\kappa }$ such that $\Delta \phi ^0(z_0)\ne 0$.

Theorem 2.8 does not ensure that the first-order constant $\Theta$ is non-zero. This is stated in the next proposition, whose proof is deferred to Appendix B.

#### Proposition 2.9

The constant $\Theta \in {\mathbb {R}}$ introduced in Theorem 2.8 satisfies $\Theta \ne 0$.

Once we have obtained the solutions of the inner equation and analyzed their difference, the next step is to “measure” how well they approximate the functions obtained in Theorems 2.2 and 2.3. This is done through what is usually called *complex matching* techniques.

We first define the *matching domains* where these differences are analyzed. Let $0<\nu <1$ and $0<\theta _2< \theta<\theta _1<\frac{\pi }{2}$, where $\theta$ is the angle introduced in (2.7). We denote

$$ \rho _{-}= -i\kappa \varepsilon + x_-, \qquad x_1^-=-i\kappa \varepsilon - \varepsilon ^{\nu } e^{i \theta _1}+ x_-, \qquad x_2^- = -i\kappa \varepsilon + \varepsilon ^{\nu } e^{i\theta _2} +x_-. $$

and

$$ \rho _{+}=-i\kappa \varepsilon + x_+. \qquad x_1^+=-i\kappa \varepsilon + \varepsilon ^{\nu } e^{-i \theta _1}+ x_+, \qquad x_2^+ = -i\kappa \varepsilon - \varepsilon ^{\nu } e^{-i\theta _2} +x_+. $$

Notice that $\rho _{+}=-\overline{\rho _{-}}$, $x_1^+=-\overline{x_1^-}$, $x_2^+ = -\overline{x_2^-}$, where we have denoted by $\overline{z}$ the complex conjugate of *z*. We define the matching domains as

**Fig. 6:**
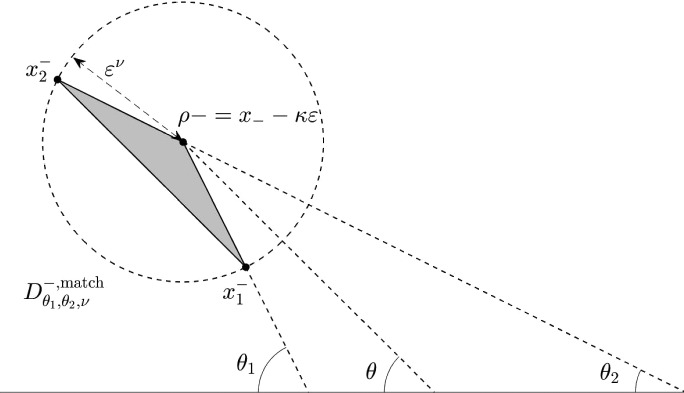
The matching domain $D^{-,\textrm{match}}_{\theta _1,\theta _2,\nu }$ introduced in (2.32)

2.32 $$\begin{aligned} D_{\theta _1,\theta _2, \nu }^{-, \textrm{match}} = \widehat{\rho _{-},\, x_1^-\,,x_2^-}, \qquad -D_{\theta _1,\theta _2,\nu }^{+, \textrm{match}} = \widehat{\rho _{+},\, x_1^+\,,x_2^+} \end{aligned}$$

that is, $D_{\theta _1,\theta _2,\nu }^{-,\textrm{match}}$ as the triangle with vertexs $\rho _{-}, x_1^-,x_2^-$ while $D_{\theta _1,\theta _2,\nu }^{-,\textrm{match}}$ is the triangle with vertexs $-\rho _{+}, x_1^+, x_2^+$ (see Fig. 6).

We also introduce

2.33 $$\begin{aligned} \begin{aligned}&\xi ^{0,\textrm{u}}_-(x) = \frac{c_{-1}}{\varepsilon }\phi ^{0,\textrm{u}}\big (\varepsilon ^{-1} (x-x_-) \big ),&\eta ^{0,\textrm{u}}_-(x)=\frac{c_{-1}}{\varepsilon ^3} \psi ^{0,\textrm{u}}\big (\varepsilon ^{-1} (x-x_-)\big ), \\&\xi ^{0,\textrm{s}}_+(x) = \frac{c_{1}}{\varepsilon }\phi ^{0,\textrm{s}}\big (\varepsilon ^{-1} (x-x_+ )\big ),&\eta ^{0,\textrm{s}}_+ (x)=\frac{c_{1}}{\varepsilon ^3 }\psi ^{0,\textrm{s}}\big (\varepsilon ^{-1} (x-x_+ )\big ) \end{aligned} \end{aligned}$$

and

2.34 $$\begin{aligned} \begin{aligned}&\xi ^{0,\textrm{aux}}_-(x) = \frac{c_{-1}}{\varepsilon } \phi ^{0,\textrm{s}}\big (\varepsilon ^{-1} (x-x_-) \big ),&\eta ^{0,\textrm{aux}}_-(x)=\frac{c_{-1}}{\varepsilon ^3 } \psi ^{0,\textrm{s}}\big (\varepsilon ^{-1} (x-x_- )\big ), \\&\xi ^{0,\textrm{aux}}_+(x) = \frac{c_{1}}{\varepsilon }\phi ^{0,\textrm{u}}\big (\varepsilon ^{-1} (x-x_+ ) \big ),&\eta ^{0,\textrm{aux}}_+ (x)=\frac{c_{1}}{\varepsilon ^3 } \psi ^{0,\textrm{u}}\big (\varepsilon ^{-1} (x-x_+ )\big ). \end{aligned}\nonumber \\ \end{aligned}$$

The following theorem, which is proved in Sect. 6, provides estimates between $(\xi ^{0,*}_\pm ,\eta ^{0,*}_\pm )$ and $(\xi ^*,\eta ^*)$ with $\star =\textrm{u},\textrm{s},\textrm{aux}$ in the corresponding matching domains.

#### Theorem 2.10

Let $\theta >0, \kappa _0$ be fixed as in Theorems 2.8, 2.3 and $\theta$ as in Theorem  2.2. Take $0<\theta _2<\theta<\theta _1< \textrm{atan}\left( \frac{\pi }{3\alpha }\right)$ and $\nu \in (0,1)$.

We introduce the functions

$$\begin{aligned}&\big (\delta \xi ^{\textrm{u}}_-, \delta \eta ^{\textrm{u}}_- \big )= \big (\xi ^{\textrm{u}}- \xi ^{0,\textrm{u}}_-, \eta ^{\textrm{u}} - \eta ^{0,\textrm{u}}_-\big ),\\&\big (\delta \xi ^{\textrm{s}}_+ , \delta \eta ^{\textrm{s}}_+ \big )= \big (\xi ^{\textrm{s}}- \xi ^{0,\textrm{s}}_+, \eta ^{\textrm{s}} - \eta ^{0,\textrm{s}}_+\big ), \\&\big (\delta \xi _ \pm ^{\textrm{aux}}, \delta \eta _\pm ^{\textrm{aux}} \big )= \big (\xi ^\textrm{aux}- \xi ^{0,\textrm{aux}}_\pm , \eta ^\textrm{aux}- \eta ^{0,\textrm{aux}}_\pm \big ). \end{aligned}$$

Then there exist $\kappa _1\ge \kappa _0$ and a constant $M_9>0$ such that for all $\kappa \ge \kappa _1$ and $x \in D_{\theta _1,\theta _2, \nu }^{\pm , \textrm{match}}$

$$\begin{aligned}&\big |\delta \xi ^{\textrm{u}}_- (x)\big | ,\, \big | \delta \xi ^{\textrm{s}}_+ (x)\big |, \big |\delta \xi ^\textrm{aux}_ \pm (x) \big | \le M_9 |\log \varepsilon |\frac{\varepsilon ^{2-\nu } }{|x-x_\pm |^2}, \\&\big | \partial _ x \delta \xi ^{\textrm{u}}_- (x)\big | ,\, \big | \partial _ x \delta \xi ^{\textrm{s}}_+(x) \big |,\, \big | \partial _ x\delta \xi ^\textrm{aux}_\pm (x) \big | \le M_9 |\log \varepsilon |\frac{\varepsilon ^{2-\nu } }{|x-x_\pm |^3} \\&\big |\delta \eta ^{\textrm{u}}_- (x)\big | ,\, \big |\delta \eta ^{\textrm{s}}_+(x)(x) \big |,\, \big |\delta \eta ^\textrm{aux}_\pm (x) \big | \le M_9 |\log \varepsilon |\frac{\varepsilon ^{2-\nu }}{|x-x_\pm |^4}, \\&\big | \partial _ x \delta \eta ^{\textrm{u}}_- (x)\big | ,\, \big |\partial _x \delta \eta ^{\textrm{s}}_+(x) \big |,\, \big | \partial _ x\delta \eta ^\textrm{aux}_\pm (x) \big | \le M_9 |\log \varepsilon |\frac{\varepsilon ^{1-\nu }}{|x-x_\pm |^4}. \end{aligned}$$

### The asymptotic formula

Now, to prove Theorem 1.2 it only remains to provide an asymptotic formula for the constants $C_1^\star$ and $C_2^\star$. This is done in the following proposition, which is proved in Sect. 2.6. From now on we take

2.35 $$\begin{aligned} \kappa =c|\log \varepsilon | \end{aligned}$$

for some suitable constant $c>0$ to be chosen later.

#### Proposition 2.11

The constants $C_1^\star$ and $C_2^\star$ introduced in Proposition 2.7 satisfy

$$\begin{aligned} \begin{aligned} C_1^{\textrm{u}}&= \frac{1}{\sqrt{|\gamma |}\varepsilon ^3}e^{-\frac{i\overline{x_-}}{\varepsilon }}\left( \Theta +{\mathcal {O}}\left( \frac{1}{|\log \varepsilon |}\right) \right) \\ C_2^{\textrm{u}}&=\frac{1}{\sqrt{|\gamma |}\varepsilon ^3}e^{\frac{ i {x_-}}{\varepsilon }}\left( \Theta +{\mathcal {O}}\left( \frac{1}{|\log \varepsilon |}\right) \right) \\ C_1^{\textrm{s}}&= -\frac{1}{\sqrt{|\gamma |}\varepsilon ^3}e^{-\frac{i\overline{x_+}}{\varepsilon }}\left( \Theta +{\mathcal {O}}\left( \frac{1}{|\log \varepsilon |}\right) \right) \\ C_2^{\textrm{s}}&=-\frac{1}{\sqrt{|\gamma |}\varepsilon ^3}e^{\frac{i{x_+}}{\varepsilon }}\left( \Theta +{\mathcal {O}}\left( \frac{1}{|\log \varepsilon |}\right) \right) . \end{aligned} \end{aligned}$$

Evaluating at $x=0$ the formula for $\Delta ^\star$ in (2.19) together with Propositions 2.7 and 2.11 lead to the asymptotic formulas

$$\begin{aligned} \begin{aligned} \Delta \eta ^{\textrm{u}}(0)&=\frac{1}{\sqrt{|\gamma |}\varepsilon ^3}e^{-\frac{\pi }{\varepsilon }}\left( 2\Theta \cos \left( \frac{\alpha }{\varepsilon }\right) +{\mathcal {O}}\left( \frac{1}{|\log \varepsilon |}\right) \right) \\ \partial _x\Delta \eta ^{\textrm{u}}(0)&=\frac{1}{\sqrt{|\gamma |}\varepsilon ^4}e^{-\frac{\pi }{\varepsilon }}\left( -2\Theta \sin \left( \frac{\alpha }{\varepsilon }\right) +{\mathcal {O}}\left( \frac{1}{|\log \varepsilon |}\right) \right) \\ \Delta \eta ^{\textrm{s}}(0)&=-\frac{1}{\sqrt{|\gamma |}\varepsilon ^3}e^{-\frac{\pi }{\varepsilon }} \left( 2\Theta \cos \left( \frac{\alpha }{\varepsilon }\right) +{\mathcal {O}}\left( \frac{1}{|\log \varepsilon |}\right) \right) \\ \partial _x\Delta \eta ^{\textrm{s}}(0)&= -\frac{1}{\sqrt{|\gamma |}\varepsilon ^4}e^{-\frac{\pi }{\varepsilon }} \left( 2\Theta \sin \left( \frac{\alpha }{\varepsilon }\right) +{\mathcal {O}}\left( \frac{1}{|\log \varepsilon |}\right) \right) , \end{aligned} \end{aligned}$$

where $\alpha$ is the constant introduced in (2.1).

To complete the proof of Theorem 1.2 we recall that $\Delta \eta =\Delta \eta ^{\textrm{u}}+\Delta \eta ^{\textrm{s}}$ and that by the symmetry properties in Theorem 2.2 and 2.3 of $\eta ^{\textrm{u}},\eta ^\textrm{s}, \eta ^\textrm{aux}$ one has that, for $x\in D^\textrm{aux}_\kappa \cap \mathbb {R}$

$$\begin{aligned} \Delta \eta ^{\textrm{u}}(x) = \eta ^\textrm{u}(x) - \eta ^\textrm{aux}(x)= \eta ^{\textrm{s}}(-x)- \eta ^\textrm{aux}(-x) = -\Delta \eta ^\textrm{s}(-x) \end{aligned}$$

and therefore $\Delta \eta ^\textrm{u}(0)= - \Delta \eta ^\textrm{s}(0)$. This completes the proof of Theorem 1.2.

#### Remark 2.12

Notice that we could argue by symmetry that $\Delta \eta ^\textrm{s}(x) = - \Delta \eta ^\textrm{u}(-x)$ and skip the constants $C_{1,2}^\textrm{s}$ of our analysis. However we have preferred to keep all constants in order to emphasize that the method does not depend on the symmetries of the system.

### Proof of Proposition 2.11

To prove Proposition 2.11, the first step is to provide an asymptotic formula for $\Delta \eta ^{\textrm{u}}(\rho _{-}), \Delta \eta ^{\textrm{u}}(\overline{\rho _{-}})$ and $\Delta \eta ^{\textrm{s}}(\rho _{+}), \Delta \eta ^{\textrm{s}}(\overline{\rho _{+}})$.

#### Lemma 2.13

Let $\nu \in (0,1)$ and consider the points $x=\rho _{-}$ and $x=\overline{\rho _{-}}$ introduced in (2.17) with $\kappa$ as in (2.35) and $c\in (0, 1-\nu )$ .

Then, the functions $\Delta \eta ^{\textrm{u}}, \Delta \eta ^\textrm{s}$ in (2.9) satisfy

$$\begin{aligned} \begin{aligned} \Delta \eta ^{\textrm{u}}(\rho _{-})&= \frac{c_{-1}}{\varepsilon ^3} {e^{-\kappa }} \left( \Theta +{\mathcal {O}}\left( \frac{1}{|\log \varepsilon |}\right) \right) \\ \Delta \eta ^{\textrm{u}}(\overline{\rho _{-}})&=\frac{{c_{-1}}}{\varepsilon ^3} {e^{-\kappa }} \left( { \Theta } +{\mathcal {O}}\left( \frac{1}{|\log \varepsilon |}\right) \right) , \end{aligned} \end{aligned}$$

and

$$\begin{aligned} \begin{aligned} \Delta \eta ^{\textrm{s}}(\rho _{+})&= \frac{c_{+1}}{\varepsilon ^3} {e^{-\kappa }} \left( \Theta +{\mathcal {O}}\left( \frac{1}{|\log \varepsilon |}\right) \right) \\ \Delta \eta ^{\textrm{s}}(\overline{\rho _{+}})&=\frac{{c_{+1}}}{\varepsilon ^3} {e^{-\kappa }} \left( {\Theta } +{\mathcal {O}}\left( \frac{1}{|\log \varepsilon |}\right) \right) , \end{aligned} \end{aligned}$$

where $c_{\pm 1}$ and $\Theta$ are the constants introduced in (2.2) and Theorem 2.8 respectively.

#### Proof

We provide the proof for $\Delta \eta ^{\textrm{u}}(\rho _{-})$. The other formula can be proven analogously. Note that $\Delta \eta ^{\textrm{u}}$ can be written as

$$\begin{aligned} \Delta \eta ^{\textrm{u}}(x)&= \eta ^\textrm{u}(x)- \eta ^{0,\textrm{u}}_-(x) + \eta ^{0,\textrm{u}}_-(x) -\eta ^{0,\textrm{aux}}_- (x) + \eta ^{0,\textrm{aux}}_-(x)-\eta ^\textrm{aux}(x)\\&= \frac{c_{-1}}{\varepsilon ^3}\Delta \psi ^0\left( \frac{x-x_-}{\varepsilon }\right) +\delta \eta _-^{\textrm{u}}(x)-\delta \eta _-^\textrm{aux}(x) \end{aligned}$$

where $\eta ^{0,\star }_-$, $\star =\textrm{u},\textrm{aux}$ are defined in (2.33), (2.34)$, \Delta \psi ^0$ is the function analyzed in Theorem 2.8 (recall the inner change of variables (2.23)) and $\delta \eta _-^{\textrm{u}}$, $\delta \eta _-^\textrm{aux}$ are the functions introduced in Theorem 2.10. Then, it is enough to use the asymptotic formula (2.31) and the estimates in Theorem 2.10. Indeed, using that $\rho _{-}- x_- = -i \kappa \varepsilon$, we obtain

$$\begin{aligned} \Delta \eta ^\textrm{u}(\rho _{-})&=\frac{c_{-1}}{\varepsilon ^3 } \left( \Theta e^{-\kappa } \big (1 + \chi (-i\kappa )\big ) + \mathcal {O}\left( \frac{\varepsilon ^{1-\nu }}{|\log \varepsilon |^3}\right) \right) \\&= \frac{c_{-1}}{\varepsilon ^3 } e^{-\kappa } \left( \Theta + \mathcal {O}\left( \frac{1}{|\log \varepsilon |} \right) + e^{\kappa }\mathcal {O}\left( \frac{\varepsilon ^{1-\nu } }{|\log \varepsilon |^3}\right) \right) \end{aligned}$$

and therefore, from $e^{\kappa } = \varepsilon ^{-c} \le \varepsilon ^{\nu -1}$, we obtain the result. Notice that

$$\begin{aligned} \Delta \eta ^{\textrm{s}}(x)&= \eta ^{\textrm{aux}}(x)- \eta ^{0,\textrm{aux}}_+(x) + \eta ^{\textrm{aux},0}_+(x) -\eta ^{0,\textrm{s}}_+ (x) + \eta ^{0,\textrm{s}}_+(x)-\eta ^\textrm{s}(x)\\&= \frac{c_{+1}}{\varepsilon ^3}\Delta \psi ^0\left( \frac{x-x_+}{\varepsilon }\right) +\delta \eta _+^{\textrm{aux}}(x)-\delta \eta _+^\textrm{s}(x) \end{aligned}$$

so the result for $\Delta \eta ^\textrm{s}$ follows analogously as the one for $\Delta \eta ^\textrm{u}$. $\square$

To complete the proof of Proposition 2.11, it suffices to solve the linear system (2.20). Indeed, we have that, the linear system for $C_{1,2}^\textrm{u}$ can be rewritten as

$$\begin{aligned} \begin{pmatrix} 1 &  e^{-\frac{2i \overline{\rho _{-}}}{\varepsilon }} \\ e^{\frac{2i \rho _{-}}{\varepsilon }} &  1 \end{pmatrix} \begin{pmatrix} C_1^\textrm{u}\\ C_2^\textrm{u}\end{pmatrix} = \begin{pmatrix} e^{-\frac{i \overline{\rho _{-}}}{\varepsilon }} \Delta \eta ^\textrm{u}(\overline{\rho _{-}}) \\ e^{\frac{i\rho _{-}}{\varepsilon }} \Delta \eta ^\textrm{u}(\rho _{-}) \end{pmatrix}. \end{aligned}$$

Thus, using that $\varepsilon ^{-1} i (\rho _{-}- \overline{\rho _{-}})=-\varepsilon ^{-1} 2\pi$, that $\rho _{-}=x_- - i\kappa \varepsilon$ and Lemma 2.13

$$\begin{aligned} C_1^\textrm{u}&= \frac{c_{-1}}{\varepsilon ^3} e^{-\frac{i\overline{x_-}}{\varepsilon }} \left( \Theta + \mathcal {O}\left( \frac{1}{|\log \varepsilon | }\right) \right) \\ C_2^\textrm{u}&= \frac{c_{-1}}{\varepsilon ^3} e^{\frac{i{x_-}}{\varepsilon }} \left( \Theta + \mathcal {O}\left( \frac{1}{|\log \varepsilon | }\right) \right) . \end{aligned}$$

Proceeding analogously for $C_{1,2}^\textrm{s}$ we obtain

$$\begin{aligned} C_1^\textrm{s}&= \frac{c_{+1}}{\varepsilon ^3} e^{-\frac{i\overline{x_+}}{\varepsilon }} \left( \Theta + \mathcal {O}\left( \frac{1}{|\log \varepsilon | }\right) \right) \\ C_2^\textrm{s}&= \frac{c_{+1}}{\varepsilon ^3} e^{\frac{i{x_+}}{\varepsilon }} \left( \Theta + \mathcal {O}\left( \frac{1}{|\log \varepsilon | }\right) \right) . \end{aligned}$$

Since $c_{\mp 1} = \pm (\sqrt{|\gamma |})^{-1}$ is given in  (2.2), this completes the proof of Proposition 2.11.

### Notation and preliminaries

The rest of the paper is devoted to prove the intermediate results in the previous sections. In order to do so, here, we set some standard notations used in our work and to provide (and prove) a general result improving the classical fixed point theorem. We will use the following notation and conventions:

- For $g,h: \Omega \subset \mathbb {C}\rightarrow \mathbb {C}$, a function defined in a complex set $\Omega$, we will say that $|g(x)|\lesssim |h(x)|$ if there exists a constant *M* such that for all $x\in \Omega$, $|g(x)|\le M |h(x)|$.
- Let *X* be a Banach space endowed with the norm $\Vert \cdot \Vert _X$. We will use the notation $B(\varrho ) \subset X$ for the closed ball of radius $\varrho$ centered at the origin of *X*, namely
  $$\begin{aligned} B(\varrho )=\{ \textbf{x} \in X: \Vert \textbf{x}\Vert _X \le \varrho \}. \end{aligned}$$
- From now on, $\kappa _0,\varepsilon >0$ will be fixed; $\kappa _0$ is as large and we need and $\varepsilon _0>0$ is as small as necessary. All the constants appearing in the results are uniform with respect to $\varepsilon \in (0,\varepsilon _0]$ and $\kappa \ge \kappa _0$. Moreover, when we say in the statement of a result, that $\varepsilon$ is small enough (resp. $\kappa$ is big enough) we mean that we are choosing $\varepsilon _0>0$ small enough (resp. $\kappa _0$ big enough) such that the statement hold for $\varepsilon \in (0,\varepsilon _0]$ (resp. $\kappa \ge \kappa _0$).
- We will denote by $\overline{D}$ the closure of a set *D*.

We present now a result which is a consequence of the Banach fixed point theorem. We will use it several times along the work.

#### Theorem 2.14

Let $(X \Vert \cdot \Vert _X ), (Y,\Vert \cdot \Vert _Y)$ be Banach spaces and take any $(\textbf{x}_0,\textbf{y}_0)\in X\times Y$. Consider $\textbf{F}: X \times Y \rightarrow X\times Y$ an operator, $\textbf{F}= (\textbf{F}_X, \textbf{F}_Y)$, satisfying that, there exist positive constants $\textbf{c}$,

$$\begin{aligned} \varrho \ge 3(\textbf{c}+1) \textrm{max}\{ \Vert {\textbf{F}}_X [\textbf{x}_0, \textbf{y}_0]-\textbf{x}_0\Vert _X, \Vert {\textbf{F}}_Y [\textbf{x}_0, \textbf{y}_0]-\textbf{y}_0)\Vert _Y \}, \end{aligned}$$

$L_1,L_2$ and $L_3$ such that

2.36 $$\begin{aligned} \begin{aligned} \Vert \textbf{F}_X[\textbf{x}, \textbf{y}]- \textbf{F}_X[\widetilde{\textbf{x}},\widetilde{\textbf{y}}] \Vert _X \le \textbf{c}\Vert \textbf{y}-\widetilde{\textbf{y}}\Vert _Y + L_1 \Vert \textbf{x} - \widetilde{\textbf{x}}\Vert _X \\ \Vert \textbf{F}_Y[\textbf{x},\textbf{y}]- \textbf{F}_Y[\widetilde{\textbf{x}},\widetilde{\textbf{y}}] \Vert _Y \le L_2 \Vert \textbf{x}-\widetilde{\textbf{x}}\Vert _X+L_3\Vert \textbf{y}-\widetilde{\textbf{y}}\Vert _Y \end{aligned} \end{aligned}$$

for any $(\textbf{x}-\textbf{x}_0,\textbf{y}- \textbf{y}_0), (\widetilde{\textbf{x}}-\textbf{x}_0, \widetilde{\textbf{y}}- {\textbf{y}}_0) \in B(\varrho ) \times B(\varrho )\subset X\times Y$. Then, if

2.37 $$\begin{aligned} L_1 + \textbf{c}(L_2+L_3),L_2+L_3\le \frac{1}{3}, \end{aligned}$$

the fixed point equation $(\textbf{x},\textbf{y}) = \textbf{F}[\textbf{x},\textbf{y}]$ restricted to $B(\varrho ) \times B(\varrho )$ has a unique solution.

#### Proof

We endow $X\times Y$ with the norm $\Vert (\textbf{x}, \textbf{y})\Vert _\times = \textrm{max}\{\Vert \textbf{x}\Vert _X, \Vert \textbf{y}\Vert _Y\}$. We notice that $B(\varrho ) \times B(\varrho ) \subset X\times Y$ is indeed the ball of radius $\varrho$ centered at the origin.

We first claim that, if $(\textbf{x}-\textbf{x}_0, \textbf{y}-\textbf{y}_0) \in B(\varrho ) \subset X\times Y$, then

$$\begin{aligned}(\textbf{x}-\textbf{x}_0,\textbf{F}_Y[\textbf{x},\textbf{y}]- \textbf{y}_0) \in B(\varrho )\subset X\times Y.\end{aligned}$$

Indeed, it is clear that

$$\begin{aligned} \Vert \textbf{F}_Y[\textbf{x},\textbf{y}]-\textbf{y}_0\Vert _Y&\le \Vert \textbf{F}_Y[\textbf{x},\textbf{y}]-\textbf{F}_Y[\textbf{x}_0,\textbf{y}_0]\Vert _Y + \Vert \textbf{F}_Y[\textbf{x}_0,\textbf{y}_0]-\textbf{y}_0\Vert _Y \\&\le \varrho \left( L_2 + L_3 + \frac{1}{3(\textbf{c}+1)} \right) \le \varrho \end{aligned}$$

where we have used that $L_2+L_3 \le \frac{1}{3}$.

Consider the operator

$$\begin{aligned} \widehat{\textbf{F}}[\textbf{x},\textbf{y}]= \big ( \textbf{F}_X(\textbf{x}, \textbf{F}_Y[\textbf{x}, \textbf{y}] ), \textbf{F}_Y[\textbf{x},\textbf{y}]\big ), \end{aligned}$$

which has the same fixed points that $\textbf{F}$, and we compute the Lipschitz constant of the operator $\widehat{\textbf{F}}$. By hypothesis we have that

$$\begin{aligned} \Vert \widehat{\textbf{F}}_X[\textbf{x}, \textbf{y}]- \widehat{\textbf{F}}_X[\widetilde{\textbf{x}},\widetilde{\textbf{y}}] \Vert _X&\le \textbf{c}\Vert \textbf{F}_Y[\textbf{x},\textbf{y}]- \textbf{F}_Y[\widetilde{\textbf{x}},\widetilde{\textbf{y}}] \Vert _Y + L_1 \Vert \textbf{x} - \widetilde{\textbf{x}}\Vert _X \\&\le \textbf{c}L_3 \Vert \textbf{y}-\widetilde{\textbf{y}}\Vert _Y + (L_1+ \textbf{c}L_2) \Vert \textbf{x} - \widetilde{\textbf{x}}\Vert _X. \end{aligned}$$

Then, denoting $$L=\textrm{max}\{ L_1+\textbf{c}L_2+\textbf{c}L_3, L_2+L_3\} $$

$$\begin{aligned} \Vert \widehat{\textbf{F}} [\textbf{x}, \textbf{y}]- \widehat{\textbf{F}}[\widetilde{\textbf{x}},\widetilde{\textbf{y}}] \Vert _\times \le L \Vert (\textbf{x},\textbf{y})\Vert _\times \end{aligned}$$

and hence, the Lipschitz constant of $\widehat{\textbf{F}}$ is $L\le \frac{1}{3}$ by hypothesis.

In addition, for $(\textbf{x}-\textbf{x}_0,\textbf{y}-\textbf{y}_0) \in B(\varrho ) \subset X\times Y$,

$$\begin{aligned} \Vert \widehat{\textbf{F}} [\textbf{x}, \textbf{y}]-(\textbf{x}_0, \textbf{y}_0)\Vert \times \le&\Vert \widehat{\textbf{F}} [\textbf{x}, \textbf{y}]- \widehat{\textbf{F}} [\textbf{x}_0, \textbf{y}_0]\Vert _\times +\Vert \widehat{\textbf{F}} [\textbf{x}_0, \textbf{y}_0] -(\textbf{x}_0, \textbf{y}_0)\Vert _\times \\ \le&L \Vert (\textbf{x}, \textbf{y})-(\textbf{x}_0, \textbf{y}_0)\Vert _\times + \Vert \widehat{\textbf{F}} [\textbf{x}_0, \textbf{y}_0]- {\textbf{F}} [\textbf{x}_0, \textbf{y}_0] \Vert _\times \\&+ \Vert {\textbf{F}} [\textbf{x}_0, \textbf{y}_0] - (\textbf{x}_0, \textbf{y}_0) \Vert _\times \\ \le&L\varrho + \Vert \textbf{F}_X[\textbf{x}_0, \textbf{F}_Y[\textbf{x}_0, \textbf{y}_0] ]-\textbf{F}_X[\textbf{x}_0,\textbf{y}_0]\Vert _X+ \frac{\varrho }{3(\textbf{c}+1)} \\ \le&\varrho \left( L+ \frac{1}{3(\textbf{c}+1)} +\frac{\textbf{c}}{3(\textbf{c}+1)} \right) \le \varrho . \end{aligned}$$

Therefore, the map $\widehat{\textbf{F}}$ is a contraction from $B(\varrho )\subset X\times Y$ to itself and the fixed point theorem implies the existence of an unique fixed point belonging to $B(\varrho ) \subset X\times Y$. $\square$

## The Invariant Manifolds in the Outer Domain

Here we prove Theorem 2.2 with a fixed point argument. Then the first step of the proof, done in Sect. 3.1, is to reformulate Theorem 2.2 as a fixed point problem. In Sect. 3.2 we prove that the fixed point operator is a contraction in a suitable closed ball of a Banach space.

We prove Theorem 2.2 for the unstable manifold and we obtain the corresponding result for the stable manifold taking advantage of the symmetries of the system. Indeed, by definition (2.7) of $D^{\textrm{out},\star }_{\kappa }$, $x\in D^{\textrm{out},\textrm{s}}_\kappa$ if and only if $-x \in D^{\textrm{out},\textrm{u}}_\kappa$ and using that the system is reversible with respect to the involution $\Psi$ in (1.13), we deduce that, if $(\xi ^\textrm{u}, \eta ^\textrm{u})$ satisfy the conditions in Theorem 2.2, then

$$\begin{aligned} \xi ^\textrm{s}(x):=\xi ^\textrm{u}(-x), \qquad \eta ^\textrm{s}(x): = \eta ^\textrm{u}(-x) \end{aligned}$$

satisfy the corresponding properties.

### The fixed point approach

For given $\kappa > 0$ and $\theta \in \left( 0, \arctan \left( \frac{\pi }{3 \alpha }\right) \right)$ we recall definition (2.7) (see also Fig. 2) of the complex domains $D^{\textrm{out},\textrm{u}}_{\kappa }$. From now on we fix $\theta$ and we do not write explicitly the dependence of the domains on $\theta$. The role of $\kappa$, as we will see, is completely different.

We introduce, for a real-analytic function $h:D^{\textrm{u},\textrm{out}}_{\kappa }\rightarrow {\mathbb {C}}$, which extends continuously to the boundary, the norm

$$\begin{aligned} \Vert h\Vert _{m,\ell }&= \displaystyle \sup _{x\in \overline{D^{\textrm{u},\textrm{out}}_{\kappa }}\cap \{ \Re (x)\le -2 \alpha \}}|\cosh x|^m |h(x)| \\&\quad + \displaystyle \sup _{x\in \overline{D^{\textrm{u},\textrm{out}}_{\kappa }}\cap \{ \Re (x)\ge -2\alpha \}}|x-x_-|^\ell |x-\overline{x}_-|^\ell |h(x)| \end{aligned}$$

with $\ell ,m\in \mathbb {R}$. Then, we define the associated Banach space

$$\begin{aligned} \mathcal {E}_{m,\ell }&=\{h: \overline{D^{\textrm{u},\textrm{out}}_{\kappa }}\rightarrow {\mathbb {C}}\text { continuous and real-analytic on }D^{\textrm{u},\textrm{out}}_{\kappa } \text { with } \Vert h\Vert _{m,\ell }<\infty \},\\ \mathcal{D}\mathcal{E}_{m,\ell }&=\{h:\overline{D^{\textrm{u},\textrm{out}}_{\kappa }}\rightarrow {\mathbb {C}}, \; h\in \mathcal {E}_{m,\ell } \text { with } \Vert h\Vert _{m,\ell }+\Vert h'\Vert _{m,\ell +1}<\infty \}, \end{aligned}$$

and the product Banach space

$$\begin{aligned} {\mathcal {E}}_\times =\mathcal{D}\mathcal{E}_{1,3}\times \mathcal {E}_{1,5}, \end{aligned}$$

with the product norm

$$\begin{aligned} \Vert (h_1,h_2)\Vert _{\times } = \textrm{max}\big \{ \Vert h_1\Vert _{1,3} + \Vert h_1'\Vert _{1,4}, \Vert h_2\Vert _{1,5} \big \}. \end{aligned}$$

We have the following lemma, whose proof is straightforward.

#### Lemma 3.1

There exists $M>0$ depending only on $\theta$, such that, for any $\kappa >0$ and $g,h:D^{\textrm{out},\textrm{u}}_{\kappa } \rightarrow {\mathbb {C}}$, it holds

1. If $$\ell _2\ge \ell _1\ge 0$$, then
  $$\begin{aligned} \Vert h\Vert _{m,\ell _2}\le M\Vert h\Vert _{m,\ell _1}\quad \text {and} \quad \Vert h\Vert _{m,\ell _1}\le \dfrac{M}{(\kappa \varepsilon )^{\ell _2-\ell _1}}\Vert h\Vert _{m,\ell _2}. \end{aligned}$$
2. If $\ell _1,\ell _2\ge 0$ and $\Vert g\Vert _{m_1,\ell _1},\Vert h\Vert _{m_2,\ell _2}<\infty$, then
  $$\Vert gh\Vert _{m_1 + m_2,\ell _1+\ell _2}\le \Vert g\Vert _{m_1,\ell _1}\Vert h\Vert _{ m_2,\ell _2}.$$
3. If $m_2 \ge m_1$, $\ell \ge 0$ and $\Vert g \Vert _{m_2,\ell } < \infty$ then
  $$\begin{aligned} \Vert g\Vert _{m_1,\ell } \le M \Vert g \Vert _{m_2,\ell }. \end{aligned}$$

In this functional setting, Theorem 2.2 (for the unstable solution) is a straightforward consequence of the following result.

#### Proposition 3.2

Consider the system (2.3), that is

3.1 $$\begin{aligned} {\mathcal {L}}_1\xi ={\mathcal {F}}_1 [\xi ,\eta ],\qquad {\mathcal {L}}_2\eta ={\mathcal {F}}_2[\xi ,\eta ] \end{aligned}$$

with ${\mathcal {L}}_1,{\mathcal {L}}_2$ and ${\mathcal {F}}=({\mathcal {F}}_1,{\mathcal {F}}_2)$ defined in (2.4) and (2.5) respectively. There exists $\kappa _0,\varepsilon _0$ and a constant $M_1$ such that for $\varepsilon \in (0,\varepsilon _0)$ and $\kappa >\kappa _0$, system (3.1) has solutions $(\xi ^\textrm{u},\eta ^\textrm{u}) \in \mathcal {E}_\times$ satisfying $\Vert (\xi ^\textrm{u},\eta ^\textrm{u})\Vert _\times \le M_1\varepsilon ^2$ and $\partial _x \xi ^\textrm{u}(0)=0$.

#### Remark 3.3

By definition of the Banach space $\mathcal {E}_\times$, since $(\xi ^\textrm{u},\eta ^\textrm{u}) \in \mathcal {E}_\times$, it satisfies the boundary conditions

3.2 $$\begin{aligned} \lim _{\Re x \rightarrow -\infty } (\xi ^{\textrm{u}}(x),\eta ^{\textrm{u}}(x)) = (0,0). \end{aligned}$$

Therefore, by Cauchy’s theorem, it is also true for *x* on $\mathbb {R}$ that

$$\begin{aligned} \lim _{x \rightarrow -\infty } (\partial _x\xi ^{\textrm{u}}(x), \partial _x \eta ^{\textrm{u}}(x)) = (0,0). \end{aligned}$$

Then,

$$\begin{aligned} \lim _{x\rightarrow -\infty } \widetilde{G}(\xi ^\textrm{u}(x), \partial _x \xi ^\textrm{u}(x), \eta ^\textrm{u}(x), \partial _x \eta ^{\textrm{u}} (x),x) = \widetilde{G}(0,0,0,0)=0, \end{aligned}$$

with $\widetilde{G}$ the first integral defined in (2.6), and therefore, for $x\in D_{\kappa }^{\textrm{out},\textrm{u}}$,

$$\begin{aligned}\widetilde{G}(\xi ^\textrm{u}(x), \partial _x \xi ^\textrm{u}(x), \eta ^\textrm{u}(x), \partial _x \eta ^{\textrm{u}}(x),x)=0.\end{aligned}$$

In addition, for $x\in D^{\textrm{out},\textrm{u}}_\kappa$, we have $|x-x_+|,|x-\overline{x_+}|\ge M$ for some constant $M>0$ and hence the estimates in Theorem 2.2 in the domain $D_\kappa ^{\textrm{out},\textrm{u}} \cap \{\Re x \ge -2 \alpha \}$ hold trivially.

The remaining part of this section is devoted to prove Proposition 3.2. In order to do so, we seek a fixed point formulation of (3.1) in a suitable ball of $\mathcal {E}_\times$. Therefore, the next step in our analysis is to look for suitable right inverses of the operators ${\mathcal {L}}_1$ and ${\mathcal {L}}_2$.

We start with ${\mathcal {L}}_1$. The homogeneous equation ${\mathcal {L}}_1\xi =0$ has two linearly independent solutions $\zeta _1$ and $\zeta _2$, where the odd function $\zeta _1(x) = u_0'(x)$ is a solution due to the translation symmetry and the even function $\zeta _2(x)$ is uniquely defined by the normalization

3.3 $$\begin{aligned} \zeta _1(x) \zeta _2'(x) - \zeta _1'(x) \zeta _2(x) = 1, \quad x \in {\mathbb {R}}, \end{aligned}$$

which follows from the Wronskian identity. The following lemma gives the second solution $\zeta _2$ and it is proved in Appendix C.

#### Lemma 3.4

For a given $\kappa >0$, there exists a unique real analytic even function $\zeta _2:D^{\textrm{out},\textrm{u}}_{\kappa } \rightarrow \mathbb {C}$ satisfying (3.3). In addition, $\zeta _2(0)\ne 0$ and $\Vert \zeta _2 \Vert _{-1,2} + \Vert \zeta _2'\Vert _{-1,3} \le M$ for some constant independent of $\kappa \ge 1$.

#### Remark 3.5

We notice that $\zeta _1 = u'_0 \in D\mathcal {E}_{1,2}$.

The classical theory of second-order differential equations implies that we can construct right inverses of the operator $\mathcal {L}_1$ as

3.4 $$\begin{aligned} \mathcal {L}_1^{-1}[h] (x) = \zeta _1(x) \left[ C_1+ \int _{x_1}^x\zeta _2(s)h(s)ds \right] + \zeta _2(x) \left[ C_2-\int _{x_2}^x\zeta _1(s)h(s) ds \right] \end{aligned}$$

for any given $x_1,x_2,C_1,C_2 \in \mathbb {R}$. However, we are interested in solutions $(\xi ^\textrm{u},\eta ^\textrm{u})$ satisfying the boundary conditions $\partial _x \xi (0)=0$ and the decay behavior  (3.2). Therefore, we impose the same conditions on the solutions of $\mathcal {L}_1 \xi = h$ and we easily obtain that the right inverse is formally given by

3.5 $$\begin{aligned} {\mathcal {G}}_1^\textrm{out}[h] (x) = \zeta _1(x)\int _0^x\zeta _2(s)h(s)ds - \zeta _2(x)\int _{-\infty }^x\zeta _1(s)h(s) ds \end{aligned}$$

where the (complex) integration path is, in the first integral, the segment between 0 and *x* and, in the second integral, corresponds to the path parameterized by $s= x+ t$, with $t\in (-\infty ,0]$.

In addition, it is straightforward to check that a right inverse of the operator $\mathcal {L}_2$ can be formally expressed as

3.6 $$\begin{aligned} {\mathcal {G}}_2^\textrm{out}[h] = -\frac{i\varepsilon }{2} e^{i\varepsilon ^{-1}x}\int _{-\infty }^x e^{-i\varepsilon ^{-1}s} h(s)ds+\frac{i\varepsilon }{2} e^{-i\varepsilon ^{-1}x}\int _{-\infty }^x e^{i\varepsilon ^{-1}s} h(s)ds, \end{aligned}$$

where the integration path is the horizontal line $s= x+ t$, $t\in (-\infty ,0]$.

The following lemma describes how the operators ${\mathcal {G}}_1^\textrm{out}$ and ${\mathcal {G}}_2^\textrm{out}$ act on functions belonging to $D\mathcal {E}_{1,3}$ and $\mathcal {E}_{1,5}$ respectively. Its proof follows the same lines as the ones of Proposition 4.3 in [31] and we sketch the main steps of the proof in Appendix C.

#### Lemma 3.6

The operators ${\mathcal {G}}_1^\textrm{out}$ and ${\mathcal {G}}_2^\textrm{out}$ introduced in (3.5) and (3.6) have the following properties.

1. $$\mathcal {G}_i^\textrm{out}\circ \mathcal {L}_i=\mathcal {L}_i\circ \mathcal {G}_i^\textrm{out}=\textrm{Id}$$.
2. For any $m>1$ and $\ell \ge 5$, there exists a constant $M>0$ independent of $\varepsilon$ and $\kappa$ such that, for every $h \in \mathcal {E}_{m,\ell }$,
  $$\begin{aligned} \left\| \mathcal {G}_1^\textrm{out}[h]\right\| _{1,\ell -2} \le M\Vert h \Vert _{m,\ell }\qquad \text {and}\qquad \left\| \partial _x\mathcal {G}_1^\textrm{out}[h]\right\| _{1,\ell -1} \le M\Vert h \Vert _{m,\ell } \end{aligned}$$
  and
  $$\begin{aligned} \partial _x \mathcal {G}_1^\textrm{out}[h](0)=0. \end{aligned}$$
  In addition, if *h* is real analytic, then ${\mathcal {G}}_1[h]$ is also real analytic.
3. For any $m\ge 1$, $\ell \ge 0$, there exists $M>0$ such that for $h \in \mathcal {E}_{m,\ell }$,
  $$\begin{aligned} \left\| {\mathcal {G}}_2^\textrm{out}[h]\right\| _{m,\ell }\le M\varepsilon ^2\Vert h \Vert _{m,\ell } \end{aligned}$$
  Moreover, when *h* is real analytic, ${\mathcal {G}}_2^\textrm{out}[h]$ is also real analytic.

In order to prove Proposition 3.2, we use Lemma 3.6 and look for solutions of (3.1) belonging to $\mathcal {E}_\times$, satisfying $\partial _x \xi (0)=0$ as fixed points of the operator

3.7 $$\begin{aligned} {\mathcal {F}}^\textrm{out}= \big ({\mathcal {G}}_1^\textrm{out}\circ {\mathcal {F}}_1,{\mathcal {G}}_2^\textrm{out}\circ {\mathcal {F}}_2 \big ) \end{aligned}$$

where ${\mathcal {F}}_i$ are the operators defined in (2.5).

### The contraction mapping

We prove Proposition 3.2 using Theorem 2.14. To do so, we study ${\mathcal {F}}^\textrm{out}[0,0]$ (Lemma 3.7) and the Lipschitz constant of ${\mathcal {F}}^\textrm{out}$ in a suitable ball $B(R\varepsilon ^2) \subset \mathcal {E}_\times$ (Lemma 3.8).

#### Lemma 3.7

There exists a constant $b_1>0$ independent of $\varepsilon$ and $\kappa$ such that

$$\begin{aligned} \Vert {\mathcal {F}}^\textrm{out}[0,0]\Vert _\times \le b_1\varepsilon ^2. \end{aligned}$$

#### Proof

From definition (2.5) of $\mathcal {F}$,

$$\begin{aligned} {\mathcal {F}}[0,0] = (0,f'(u_0) (u_0 - f(u_0)) + f''(u_0) (u'_0)^2). \end{aligned}$$

Since $u_0 \in \mathcal {E}_{1,1}$, see (1.7) and Lemma 2.1, and $f(u)= u^2 + 2 \gamma u^3$, ${\mathcal {F}}_2[0,0] \in \mathcal {E}_{2,5} \subset \mathcal {E}_{1,5}$ with $\Vert {\mathcal {F}}_2[0,0]\Vert _{1,5} \lesssim 1$ and from Lemma 3.6 the result holds true. $\square$

#### Lemma 3.8

There exists $C_1>0$ such that for all $R>0$, if $(\xi ,\eta ),(\widetilde{\xi },\widetilde{\eta })\in B(R\varepsilon ^2)\subset {\mathcal {E}}_\times$, then the operator ${\mathcal {F}}^\textrm{out}$ in (3.7) satisfies

$$\begin{aligned} \begin{aligned} \left\| {\mathcal {F}}_1^\textrm{out}[\xi ,\eta ]-{\mathcal {F}}_1^\textrm{out}(\widetilde{\xi },\widetilde{\eta })\right\| _{1,3}&\le C_1 \Vert \eta -\widetilde{\eta }\Vert _{1,5}+\frac{C}{\kappa ^2}\Vert (\xi ,\eta )-(\widetilde{\xi },\widetilde{\eta })\Vert _\times \\ \left\| \partial _x{\mathcal {F}}_1^\textrm{out}[\xi ,\eta ]-\partial _x{\mathcal {F}}_1^\textrm{out}[\widetilde{\xi },\widetilde{\eta }]\right\| _{1,4}&\le C_1 \Vert \eta -\widetilde{\eta }\Vert _{1,5}+\frac{C}{\kappa ^2}\Vert (\xi ,\eta )-(\widetilde{\xi },\widetilde{\eta })\Vert _\times \\ \left\| {\mathcal {F}}_2^\textrm{out}[\xi ,\eta ]-{\mathcal {F}}_2^\textrm{out}[\widetilde{\xi },\widetilde{\eta }]\right\| _{1,5}&\le \frac{C}{{\kappa ^2}}\Vert (\xi ,\eta )-(\widetilde{\xi },\widetilde{\eta })\Vert _\times . \end{aligned} \end{aligned}$$

for some constant $C=C(R)>0$ independent of $\varepsilon$ and $\kappa$.

#### Proof

Let $(\xi ,\eta ), (\widetilde{\xi }, \widetilde{\eta }) \in B(R \varepsilon ^2)$. We define $\zeta _\lambda = (\xi _\lambda , \eta _\lambda )=(\widetilde{\xi }, \widetilde{\eta }) + \lambda \big (( \xi ,\eta ) - (\widetilde{\xi }, \widetilde{\eta })\big )$. Then, using the mean value theorem

$$\begin{aligned} {\mathcal {F}}_1 [\xi , \eta ] (x) - {\mathcal {F}}_1 [\widetilde{\xi },\widetilde{\eta }] (x) = \int _{0}^1 D {\mathcal {F}}_1 [\zeta _\lambda ](x) \big ( \xi (x)- \widetilde{\xi }(x),\eta (x)- \widetilde{\eta }(x) \big )^\top \,d\lambda \end{aligned}$$

with

$$\begin{aligned} D {\mathcal {F}}_1[\zeta _\lambda ] (x) = \big (\partial _\xi {\mathcal {F}}_1[\zeta _\lambda ](x), \partial _{\eta } {\mathcal {F}}_1 [\zeta _\lambda ](x) \big )=\big (12 \gamma u_0(x) \xi _\lambda (x) + 2 \xi _\lambda (x) + 6 \gamma \xi _\lambda ^2(x), -1 \big ) \end{aligned}$$

and satisfying

$$\begin{aligned} \Vert \partial _\xi {\mathcal {F}}_1[\zeta _\lambda ] \Vert _{1,2} \lesssim \frac{\varepsilon ^2}{(\varepsilon \kappa )^2} + \frac{\varepsilon ^2}{\varepsilon \kappa } + \frac{\varepsilon ^4}{(\varepsilon \kappa )^4} \lesssim \frac{1}{\kappa ^2}, \end{aligned}$$

where we have used Lemma 3.1, that $\kappa$ is big enough and that $\varepsilon$ is small enough. Then by the second item in Lemma 3.6 and recalling that ${\mathcal {F}}_1^\textrm{out}= {\mathcal {G}}_1^\textrm{out}\circ {\mathcal {F}}_1$

$$\begin{aligned} \Vert {\mathcal {F}}_1^\textrm{out}[\xi ,\eta ]- {\mathcal {F}}_1^\textrm{out}[\widetilde{\xi }, \widetilde{\eta }]\Vert _{1,3}&\le M \Vert {\mathcal {F}}_1 [\xi ,\eta ]- {\mathcal {F}}_1[\widetilde{\xi }, \widetilde{\eta }] \Vert _{1,5}\\&\le M \Vert \widetilde{\eta }- \eta \Vert _{1,5} + \frac{C}{\kappa ^2} \Vert \xi - \widetilde{\xi }\Vert _{1,3} \end{aligned}$$

where *M* is the constant provided in item (2) of Lemma 3.6, which is independent on *R*. In addition, using again item (2) in Lemma 3.6

$$\begin{aligned} \Vert \partial _x {\mathcal {F}}_1^\textrm{out}[\xi ,\eta ]- \partial _x {\mathcal {F}}_1^\textrm{out}[\widetilde{\xi }, \widetilde{\eta }]\Vert _{1,4} \le M\Vert \widetilde{\eta }- \eta \Vert _{1,5} + \frac{C}{\kappa ^2} \Vert \xi - \widetilde{\xi }\Vert _{1,3}. \end{aligned}$$

With respect to the second component, we define

$$\begin{aligned} \mathcal {M}[\xi ,\eta ,\xi '] = f'(u_0+\xi )\left( u_0+\xi +\eta -f(u_0+\xi )\right) +f''(u_0+\xi )(u_0'+\xi ')^2 \end{aligned}$$

which satisfies $\mathcal {M}[\xi ,\eta ,\xi '] = {\mathcal {F}}_2[\xi ,\eta ]$. We note that $\Vert u_0 + \xi _\lambda \Vert _{1,1},\Vert u_0'+\xi _\lambda '\Vert _{1,2}\lesssim 1$. Then, computing

$$\begin{aligned} \partial _{\xi '} \mathcal {M}[\xi _\lambda ,\eta _\lambda ,\xi _\lambda '] =2 f''(u_0+\xi _\lambda ) (u_0'+ \xi _{\lambda }'), \end{aligned}$$

we have that

$$\begin{aligned} \Vert \partial _{\xi '} \mathcal {M}[\xi _\lambda ,\eta _\lambda ,\xi _\lambda '] \Vert _{2,1} \lesssim \frac{1}{(\kappa \varepsilon )^{2}}. \end{aligned}$$

In addition

$$\begin{aligned} \Vert \partial _{\eta } \mathcal {M}[\xi _\lambda ,\eta _\lambda ,\xi _\lambda '] \Vert _{2,0} \lesssim \frac{1}{(\kappa \varepsilon )^{2}},\qquad \Vert \partial _{\xi } \mathcal {M}[\xi _\lambda ,\eta _\lambda ,\xi _\lambda '] \Vert _{2,2} \lesssim \frac{1}{(\kappa \varepsilon )^{2}}. \end{aligned}$$

Then, using the mean’s value theorem as Lemma 3.1, we obtain

$$\begin{aligned} \Vert {\mathcal {F}}_2 [\xi ,\eta ] - {\mathcal {F}}_2[\widetilde{\xi },\widetilde{\eta }] \Vert _{1,5} \lesssim \frac{1}{( \kappa \varepsilon )^2} \Vert (\xi , \eta ) - (\widetilde{\xi }, \widetilde{\eta })\Vert _\times , \end{aligned}$$

from which the last bound in Lemma 3.8 follows by recalling that ${\mathcal {F}}^{\textrm{out}}_2 = \mathcal {G}_2^{\textrm{out}} \circ {\mathcal {F}}_2$ and applying the third item of Lemma 3.6. $\square$

#### End of the proof of Proposition 3.2

We apply now Theorem 2.14 to the operator $\mathcal {F}^\textrm{out}$. Indeed, using Lemmas 3.7 and 3.8, we take (with the notation in Theorem 2.14) $(\textbf{x}_0,\textbf{y}_0)=(0,0)$, $\textbf{c}=C_1$,

$$\begin{aligned} \varrho =3(C_1+1) b_1 \varepsilon ^2\ge 3(C_1+1) \Vert \mathcal {F}^\textrm{out}[0,0]\Vert _\times \end{aligned}$$

and $L_1=L_2=L_3= \frac{C}{\kappa ^2}$. Hence the conditions (2.36) and (2.37) in Theorem 2.14 are trivially satisfied taking $\kappa$ big enough. Therefore, $\mathcal {F}^\textrm{out}$ has a unique fixed point which belongs to $B(3(C_1+1) b_1\varepsilon ^2)$. This completes the proof of Proposition 3.2.

## An Auxiliary Solution

Here we prove Theorem 2.3 by constructing a real-analytic solution $(\xi ^\textrm{aux},\eta ^\textrm{aux})$ of equation (2.3) defined in the domain $D^\textrm{aux}_\kappa$, see (2.10) and Fig. 3. As we have done in Sect. 3, we fix $\theta \in \left( 0, \textrm{arctan}\left( \frac{\pi }{\alpha } \right) \right)$ and we omit the dependence on it along the proof. We will run the fixed point argument similar to that of Sect. 3. Note however that we have to modify some arguments in a suitable way so that

- The integrals defining the right inverse of the linear operators $\mathcal {L}_1,\mathcal {L}_2$ have to be over paths within the new domain $D^\textrm{aux}_\kappa$, see (3.5) and (3.6).
- We have to ensure that the solutions belongs to the 0 level curve of the first integral $\widetilde{G}$ given by (2.6).

### The fixed point approach

We first define the Banach space where the fixed point argument is carried out. Given $\kappa > 0$, we define for a real-analytic function $h:D^{\textrm{aux}}_{\kappa }\rightarrow {\mathbb {C}}$ which extends continuously to the boundary, the norm

4.1 $$\begin{aligned} \Vert h\Vert _{\ell } = \displaystyle \sup _{x\in \overline{D^{\textrm{aux}}_{\kappa }}}|(x-x_-)^\ell (x-\overline{x}_-)^\ell (x-x_+)^\ell (x-\overline{x}_+)^\ell h(x)|, \end{aligned}$$

with the associated Banach spaces

4.2 $$\begin{aligned} \begin{aligned} \mathcal {Y}_{\ell }&=\{h:\overline{D^{\textrm{aux}}_{\kappa }}\rightarrow {\mathbb {C}}\text { continuous and real-analytic on } D^{\textrm{aux}}_{\kappa } \text { with } \Vert h\Vert _{\ell }<\infty \},\\ \mathcal{D}\mathcal{Y}^1_{\ell }&=\{h:\overline{D^{\textrm{aux}}_{\kappa }}\rightarrow {\mathbb {C}},\; h\in \mathcal {Y}_{\ell }\; \text {with } \Vert h\Vert _{\ell }+\Vert h'\Vert _{\ell +1}<\infty \},\\ \mathcal{D}\mathcal{Y}^2_{\ell }&=\{h:\overline{D^{\textrm{aux}}_{\kappa }}\rightarrow {\mathbb {C}}, \; h\in \mathcal {Y}_{\ell }\; \text {with } \Vert h\Vert _{\ell }+\varepsilon \Vert h'\Vert _{\ell }<\infty \}. \end{aligned} \end{aligned}$$

Then, we define the product Banach space

$$\begin{aligned} {\mathcal {Y}}_\times =\mathcal{D}\mathcal{Y}^1_{3}\times \mathcal{D}\mathcal{Y}^2_{5} \end{aligned}$$

with the norm

$$\begin{aligned} \Vert (\xi ,\eta )\Vert _\times = \textrm{max}\big \{\Vert \xi \Vert _{3}+\Vert \xi '\Vert _{4}, \Vert \eta \Vert _{5}+\varepsilon \Vert \eta '\Vert _{5}\big \}. \end{aligned}$$

The counterpart of Lemma 3.1 for the Banach spaces $\mathcal {Y}_\ell$ is the following result whose proof is left to the reader.

#### Lemma 4.1

There exists $M>0$, such that, for any $\kappa >0$ and $g,h:D^{\textrm{aux}}_{\kappa } \rightarrow {\mathbb {C}}$, it holds that

1. If $$\ell _2\ge \ell _1\ge 0$$, then
  $$\begin{aligned}\Vert h\Vert _{\ell _2}\le M\Vert h\Vert _{\ell _1}\quad \text {and} \quad \Vert h\Vert _{\ell _1}\le \dfrac{M}{(\kappa \varepsilon )^{\ell _2-\ell _1}}\Vert h\Vert _{\ell _2}.\end{aligned}$$
2. If $\ell _1,\ell _2\ge 0$ and $\Vert g\Vert _{\ell _1},\Vert h\Vert _{\ell _2}<\infty$, then
  $$\Vert gh\Vert _{\ell _1+\ell _2}\le \Vert g\Vert _{\ell _1}\Vert h\Vert _{\ell _2}.$$

We rephrase Theorem 2.3 as the following proposition.

#### Proposition 4.2

There exist $\kappa _0, \varepsilon _0>0$ and $M_2>0$, such that, if $\varepsilon \in (0, \varepsilon _0)$ and $\kappa > \kappa _0$, the system (2.3) has real-analytic solutions $(\xi ^\textrm{aux},\eta ^\textrm{aux}) \in \mathcal {Y}_\times$ satisfying

$$\begin{aligned} \widetilde{G}(\xi ^\textrm{aux},\partial _x \xi ^\textrm{aux},\eta ^\textrm{aux},\partial _x \eta ^\textrm{aux},x)=0, \qquad \partial _x \xi ^\textrm{aux}(0)=0, \end{aligned}$$

where $\widetilde{G}$ is the first integral introduced in (2.6), and $\Vert (\xi ^\textrm{aux},\eta ^\textrm{aux})\Vert _\times \le M_2 \varepsilon ^2$. In addition, $\xi ^\textrm{aux}(x)=\xi ^\textrm{aux}(-x)$ and $\eta ^\textrm{aux}(x)= \eta ^\textrm{aux}(-x)$.

To prove Proposition 4.2, we recall that system (2.3) is

$$\begin{aligned} \mathcal {L}_1 \xi = {\mathcal {F}}_1[\xi ,\eta ], \qquad \mathcal {L}_2 \eta = {\mathcal {F}}_2[\xi ,\eta ] \end{aligned}$$

with $\mathcal {L}_1, \mathcal {L}_2$ and ${\mathcal {F}}=({\mathcal {F}}_1,{\mathcal {F}}_2)$ defined in (2.4) and (2.5) respectively. Therefore, in order to set up the fixed point equation, we first introduce the suitable right inverses of the linear operators $\mathcal {L}_1, \mathcal {L}_2$. We use the fundamental set of solutions $\zeta _1=u_0'$ and the analytic continuation of $\zeta _2$ (see Lemma 3.4). The following lemma specifies another suitable property for $\zeta _2$, and it is proved in Appendix C.

#### Lemma 4.3

The even function $\zeta _2$ in Lemma 3.4 has an even analytic continuation to $D_\kappa ^\textrm{aux}$. In addition, $\zeta _2 \in D\mathcal {Y}^1_2$.

We define now the linear operators

4.3 $$\begin{aligned} \begin{aligned} {{\mathcal {G}}}^\textrm{aux}_1[h](x)&= \zeta _1(x)\int _0^x\zeta _2(s)h(s)ds - \zeta _2(x)\int _{0}^x\zeta _1(s)h(s) ds,\\ {{\mathcal {G}}}_2^\textrm{aux}[h](x)&= -\frac{i\varepsilon }{2} e^{i\varepsilon ^{-1}x}\int _{-i\rho }^x e^{-i\varepsilon ^{-1}s} h(s)ds+\frac{i\varepsilon }{2} e^{-i\varepsilon ^{-1}x}\int _{i\rho }^x e^{i\varepsilon ^{-1}s} h(s)ds, \end{aligned} \end{aligned}$$

where $\rho =\rho (\theta )= \alpha _+ \tan \theta + \pi - \kappa \varepsilon$ with $\alpha _+ = \Re x_+$, the superior vertex of $D_\kappa ^\textrm{aux}$.

The following lemma gives estimates for the linear operators ${{\mathcal {G}}}_1^\textrm{aux}, {{\mathcal {G}}}_2^\textrm{aux}$. Its proof follows the same lines as the one of Lemma 3.6 and it is deferred to Appendix C.

#### Lemma 4.4

The operators ${{\mathcal {G}}}_1^\textrm{aux}$ and ${{\mathcal {G}}}_2^\textrm{aux}$ introduced in (4.3) have the following properties.

1. $$\mathcal {L}_i\circ {{\mathcal {G}}}_i^\textrm{aux}[\xi ]=\xi $$.
2. For any $\ell \ge 5$, there exists a constant $M>0$ independent of $\varepsilon$ and $\kappa$ such that, for every $h \in {\mathcal {Y}}_{\ell }$,
  $$\begin{aligned} \left\| {{\mathcal {G}}}_1^\textrm{aux}[h]\right\| _{\ell -2} \le M\Vert h \Vert _{\ell }\qquad \text {and}\qquad \left\| \partial _x{{\mathcal {G}}}_1^\textrm{aux}[h]\right\| _{\ell -1} \le M\Vert h \Vert _{\ell }. \end{aligned}$$
  In addition, if *h* is real analytic, ${{\mathcal {G}}}_1^\textrm{aux}[h]$ is real analytic.
3. For any $\ell \ge 0$, there exists $M>0$ such that for every $h \in \mathcal {Y}_{\ell }$,
  $$\begin{aligned} \left\| {{\mathcal {G}}}_2^\textrm{aux}[h]\right\| _{\ell }\le M\varepsilon ^2\Vert h \Vert _{\ell }, \quad \left\| \partial _x {{\mathcal {G}}}_2^\textrm{aux}[h]\right\| _{\ell }\le M\varepsilon \Vert h \Vert _{ \ell } \end{aligned}$$
  When *h* is real analytic, ${{\mathcal {G}}}_2^\textrm{aux}[h]$ is also real analytic.

Now, to set up the fixed point argument we proceed in two steps so that we fix the $\widetilde{G}$ level curve. For the $\eta$ component, we just impose that it satisfies

$$\begin{aligned} \eta ={{\mathcal {G}}}_2^\textrm{aux}\circ {\mathcal {F}}_2[\xi ,\eta ]. \end{aligned}$$

Note that, then in particular,

$$\begin{aligned} \eta (0)={{\mathcal {G}}}_2^\textrm{aux}\circ {\mathcal {F}}_2[\xi ,\eta ](0). \end{aligned}$$

We use this equality to fix $\widetilde{G}$ at $x=0$. Indeed, as we claimed in (3.4), ${\mathcal {L}}_1$ in (2.4) has several right inverses

$$\begin{aligned} \mathcal {L}_1^{-1} [h] = \zeta _1(x)\left[ C_1+\int _0^x\zeta _2(s)h(s)ds\right] - \zeta _2(x)\left[ C_2+\int _{0}^x\zeta _1(s)h(s) ds\right] . \end{aligned}$$

The condition $\xi '(0)=0$ implies that one has to impose $C_1=0$ (recall that $\zeta _2$ is an even function, see Lemma 3.4). We choose a suitable $C_2$ so that the solution lies in $\widetilde{G}=0$. Indeed, we have

$$\begin{aligned} \widetilde{G}\left( -\zeta _2(0)C_2, 0,\eta (0),\eta '(0),0\right) =0. \end{aligned}$$

The following lemma ensures that, for a given $\eta$ and $\xi$ in a suitable Banach space, there exists a unique $C_2$ satisfying this equality.

#### Lemma 4.5

Fix $R>0$. There exists $\varepsilon _0$ such that for $\varepsilon \in (0,\varepsilon _0)$, there is a function ${\mathcal {I}}: B(R\varepsilon ^2)\subset {\mathcal {D}}{\mathcal {Y}}^2_5\rightarrow {\mathbb {C}}$ such that, for any $\eta \in B(R\varepsilon ^2)$,

4.4 $$\begin{aligned} \widetilde{G}\left( -\zeta _2(0){\mathcal {I}}[\eta ], 0,\eta (0),\eta '(0), 0\right) =0 \end{aligned}$$

and

$$\begin{aligned} \big |{\mathcal {I}}[\eta ] \big | \lesssim \varepsilon ^2. \end{aligned}$$

Moreover, for any $\eta ,\widetilde{\eta }\in B(R\varepsilon ^2)\subset {\mathcal {D}}{\mathcal {Y}}^2_5$,

$$\begin{aligned} |{\mathcal {I}}[\eta ]-{\mathcal {I}}[\widetilde{\eta }]|\lesssim \varepsilon ^2 \Vert \eta -\widetilde{\eta }\Vert _5. \end{aligned}$$

#### Proof

The proof follows by an implicit function theorem. Take $\eta \in B(R\varepsilon ^2)\subset {\mathcal {Y}}_5$ and denote $\eta _0=\eta (0)$ which satisfies $|\eta _0|\lesssim \varepsilon ^2$. Then, since $u_0'(0)=0$, see (1.7), equation (4.4) is equivalent

$$\begin{aligned} 0=\textbf{G}(\xi _0,\varepsilon ; \eta _0)= -u_0''(0) \xi _0 - \frac{\varepsilon ^2}{2} (\eta _0 +u_0(0) + \xi _0 -f(u_0(0)+ \xi _0) + \widetilde{\textbf{G}} (\xi _0) \end{aligned}$$

with $|\widetilde{\textbf{G}}(\xi _0) |\lesssim |\xi _0|^2$. It is clear that $\textbf{G}(0,0;\eta )=0$, then, recalling that $u_0''(0)\ne$ (see Lemma 2.1), the implicit function theorem assures, for $\varepsilon$ small enough, the existence of $\xi _0=\xi _0(\varepsilon ; \eta _0)$, satisfying $|\xi _0|\lesssim \varepsilon ^2$. In addition, since $|\partial _{\eta _0} \xi _0(\varepsilon ; \eta _0)| \lesssim \varepsilon ^2$, $|\xi _0(\varepsilon ;\eta _0)- \xi _0(\varepsilon ;\widetilde{\eta }_0)| \lesssim \varepsilon ^2 |\eta _0- \widetilde{\eta }_0|$ for any $|\eta _0|, |\widetilde{\eta }_0| \lesssim \varepsilon ^2$. Taking $\mathcal {I}[\eta ] = -\xi _0(\varepsilon ;\eta (0)) (\zeta _2(0))^{-1}$, the result follows provided $|\eta (0)|\lesssim \Vert \eta \Vert _5$. $\square$

Based on the results of Lemmas 4.4 and 4.5, we look for the functions $(\xi ^\textrm{aux},\eta ^\textrm{aux})$ in Proposition 4.2 as fixed points of the operator

4.5 $$\begin{aligned} {\mathcal {F}}^\textrm{aux}[\xi ,\eta ]= \begin{pmatrix} {\mathcal {F}}_1^\textrm{aux}[\xi ,\eta ]\\ {\mathcal {F}}_2^\textrm{aux}[\xi ,\eta ] \end{pmatrix}= \begin{pmatrix} -\zeta _2 \cdot {\mathcal {I}}[\eta ]+{{\mathcal {G}}}_1^\textrm{aux}\circ {\mathcal {F}}_1[\xi ,\eta ]\\ {{\mathcal {G}}}_2^\textrm{aux}\circ {\mathcal {F}}_2 [\xi ,\eta ] \end{pmatrix} \end{aligned}$$

with ${{\mathcal {G}}}_1^\textrm{aux}, {{\mathcal {G}}}_2^\textrm{aux}$ defined in (4.3), $\zeta _2$ defined by Lemma 4.3 and $\mathcal {F}=(\mathcal {F}_1,\mathcal {F}_2)$ is given in (2.5).

### The contraction mapping

The following two lemmas analyze the operator ${\mathcal {F}}^\textrm{aux}$ defined in (4.5).

#### Lemma 4.6

There exists a constant $b_2>0$ independent of $\varepsilon$ and $\kappa$ such that

$$\begin{aligned} \Vert {\mathcal {F}}^\textrm{aux}[0,0]\Vert _\times \le b_2\varepsilon ^2. \end{aligned}$$

#### Lemma 4.7

There exists $C_2$ such that for all $R>0$, if $(\xi ,\eta ),(\widetilde{\xi },\widetilde{\eta })\in B(R\varepsilon ^2)\subset {\mathcal {Y}}_\times$, the operator ${\mathcal {F}}^\textrm{aux}$ in (4.5) satisfies

$$\begin{aligned} \begin{aligned} \left\| {\mathcal {F}}^\textrm{aux}_1[\xi ,\eta ]-{\mathcal {F}}^\textrm{aux}_1[\widetilde{\xi },\widetilde{\eta }]\right\| _{3}&\le C_2 \Vert \eta -\widetilde{\eta }\Vert _{5}+\frac{C}{\kappa ^2}\Vert (\xi ,\eta )-(\widetilde{\xi },\widetilde{\eta })\Vert _\times , \\ \left\| \partial _x{\mathcal {F}}^\textrm{aux}_1 [\xi ,\eta ]-\partial _x{\mathcal {F}}^\textrm{aux}_1[\widetilde{\xi },\widetilde{\eta }]\right\| _{4}&\le C_2 \Vert \eta -\widetilde{\eta }\Vert _{5}+\frac{C}{\kappa ^2}\Vert (\xi ,\eta )-(\widetilde{\xi },\widetilde{\eta })\Vert _\times , \\ \left\| {\mathcal {F}}^\textrm{aux}_2 [\xi ,\eta ]-\widehat{\mathcal {F}}^\textrm{aux}_2[\widetilde{\xi },\widetilde{\eta }]\right\| _{5}&\le \frac{C}{\kappa ^2}\Vert (\xi ,\eta )-(\widetilde{\xi },\widetilde{\eta })\Vert _\times , \\ \left\| \partial _x{\mathcal {F}}^\textrm{aux}_2 [\xi ,\eta ]-\partial _x{\mathcal {F}}^\textrm{aux}_2[\widetilde{\xi },\widetilde{\eta }]\right\| _{5}&\le \frac{C}{\varepsilon \kappa ^2}\Vert (\xi ,\eta )-(\widetilde{\xi },\widetilde{\eta })\Vert _\times , \end{aligned} \end{aligned}$$

for some constant $C=C(R)>0$ independent of $\varepsilon$ and $\kappa$.

The proofs of Lemmas 4.6 and 4.7, using Lemmas 4.4 and 4.5 follow exactly the same lines as Lemma 3.7 and 3.8 and are left to the reader.

As in Sect. 3, the Lipschitz constant for ${\mathcal {F}}^\textrm{aux}$ obtained in Lemma 4.7 is not smaller than one. To overcome this problem we use Theorem 2.14 to establish that ${\mathcal {F}}^\textrm{aux}$ has a unique fixed point $(\xi ^\textrm{aux},\eta ^\textrm{aux})$ belonging to the ball $B(3(C_2+1) b_2 \varepsilon ^2)$.

Let $\widetilde{\xi }^\textrm{aux}, \widetilde{\eta }^\textrm{aux}$ be such that

$$\begin{aligned} \widetilde{\xi }^\textrm{aux}(x)=\xi ^{\textrm{aux}}(-x), \qquad \widetilde{\eta }^\textrm{aux}(x)=\eta ^\textrm{aux}(-x). \end{aligned}$$

It is clear that $(\widetilde{\xi }^\textrm{aux}, \widetilde{\eta }^\textrm{aux}) \in B(3(C_2+1) b_2 \varepsilon ^2)$ provided the auxiliary domain $D^\textrm{aux}_\kappa$ is symmetric with respect to $\{\Re x =0\}$ and $\{\Im x=0\}$. Therefore, by uniqueness of the solution of the fixed point equation $(\xi , \eta )= \mathcal {F}^\textrm{aux}[\xi ,\eta ]$, in the ball $B(3(C_2+1) b_2 \varepsilon ^2)$, in order to finish the proof of Proposition 4.2, we only need to argue that $(\widetilde{\xi }^\textrm{aux}, \widetilde{\eta }^\textrm{aux})$ is also a solution of this fixed point equation. For that we emphasize that

$$\begin{aligned} {\mathcal {F}}^\textrm{aux}_1[\widetilde{\xi }^\textrm{aux},\widetilde{\eta }^\textrm{aux}](x)={\mathcal {F}}^{\textrm{aux}}_1[\xi ^\textrm{aux}, \eta ^\textrm{aux}](-x). \end{aligned}$$

Indeed, from definition (2.5),

$$\begin{aligned} {\mathcal {F}}_1[\widetilde{\xi }^\textrm{aux}, \widetilde{\eta }^\textrm{aux}](x) = {\mathcal {F}}_1[\xi ^\textrm{aux},\eta ^\textrm{aux}](-x), \qquad {\mathcal {F}}_2[\widetilde{\xi }^\textrm{aux}, \widetilde{\eta }^\textrm{aux}](x) = {\mathcal {F}}_2[\xi ^\textrm{aux},\eta ^\textrm{aux}](-x) \end{aligned}$$

and from definition (4.3) of ${\mathcal {G}}_1^\textrm{aux},{\mathcal {G}}_2^\textrm{aux}$ and Lemma 4.3, denoting $\widetilde{h}(x) = h(-x)$, we easily prove that

$$\begin{aligned} {\mathcal {G}}_1^\textrm{aux}[\widetilde{h}](x) = {\mathcal {G}}_1^\textrm{aux}[h](-x), \qquad {\mathcal {G}}_2^\textrm{aux}[\widetilde{h}](x) = {\mathcal {G}}_2^\textrm{aux}[h](-x). \end{aligned}$$

In addition, it follows from definition (2.6) of $\widetilde{G}$ and Lemma 4.5 that $\mathcal {I}[\widetilde{\eta }^\textrm{aux}] = \mathcal {I}[\eta ^{\textrm{aux}}]$ provided

$$\begin{aligned} \widetilde{G} (\xi _0,0,\eta _0, \eta _0',0)=\widetilde{G}(\xi _0,0,\eta _0,-\eta _0',0), \qquad \forall \xi _0,\eta _0,\eta _0' \in \mathbb {R}. \end{aligned}$$

This completes the proof of Proposition 4.2.

## The Inner Equation

Here we prove Theorem 2.8 with item (1) proved in Sect. 5.1 and item (2) proved in Sect. 5.2.

### The solutions of the inner equation

Given $\ell \ge 0$ and an analytic function $f: D^{\textrm{u},\textrm{in}}_{\theta ,\kappa }\rightarrow {\mathbb {C}}$, which extends continuously to the boundary and where $D^{\textrm{u},\textrm{in}}_{\theta ,\kappa }$ is given in (2.29), consider the norm

5.1 $$\begin{aligned} \Vert f\Vert _{\ell }=\displaystyle \sup _{z\in \overline{D^{\textrm{u},\textrm{in}}_{\theta ,\kappa }}}|z^{\ell }f(z)|, \end{aligned}$$

and the Banach spaces

$$\begin{aligned} \begin{aligned} \mathcal {X}_{\ell }&=\{f: \overline{D^{\textrm{u},\textrm{in}}_{\theta ,\kappa }}\rightarrow {\mathbb {C}};\ f \text { is continuous and real-analytic on}\; D^{\textrm{u},\textrm{in}}_{\theta ,\kappa } \; \text {with }\Vert f\Vert _{\ell }<\infty \},\\ \mathcal{D}\mathcal{X}_{\ell }&=\{f:\overline{D^{\textrm{u},\textrm{in}}_{\theta ,\kappa }}\rightarrow {\mathbb {C}};\ f \in \mathcal {X}_{\ell } \; \text {with }\Vert f\Vert _{\ell }+\Vert f'\Vert _{\ell +1}<\infty \}. \end{aligned} \end{aligned}$$

We also define the product space

$$\begin{aligned} \mathcal {X}_\times =\mathcal {D}\mathcal {X}_{3} \times \mathcal {X}_5 \end{aligned}$$

endowed with the norm

$$\begin{aligned} \Vert (\phi ,\psi )\Vert _\times = \textrm{max}\big \{\Vert \phi \Vert _3+ \Vert \phi '\Vert _4, \Vert \psi \Vert _5\big \}. \end{aligned}$$

The proof of the following lemma can be found in [4].

#### Lemma 5.1

Given analytic functions $g,h:D^{\textrm{u},\textrm{in}}_{\theta ,\kappa }\rightarrow {\mathbb {C}}$, the following statements hold for some constant $M > 0$ depending only on $\theta$,

1. If $$\ell _1\ge \ell _2\ge 0$$, then
  $$\Vert h\Vert _{\ell _1-\ell _2}\le \dfrac{M}{\kappa ^{\ell _2}}\Vert h\Vert _{\ell _1}.$$
2. If $\ell _1,\ell _2\ge 0$, and $\Vert g\Vert _{\ell _1},\Vert h\Vert _{\ell _2}<\infty$, then
  $$\Vert g h\Vert _{\ell _1+\ell _2}\le \Vert g\Vert _{\ell _1}\Vert h\Vert _{\ell _2}.$$
3. If $h\in \mathcal {X}_{\ell }$ (with respect to the inner domain $D^{\textrm{u},\textrm{in}}_{\theta ,\kappa }$), then $\partial _zh\in \mathcal {X}_{\ell +1}$ (with respect to the inner domain $D^{\textrm{u},\textrm{in}}_{2\theta ,4\kappa }$), and
  $$\Vert \partial _zh\Vert _{\ell +1}\le M\Vert h\Vert _{\ell }.$$

The first item in Theorem 2.8 is now rewritten as the following proposition.

#### Proposition 5.2

Consider system (2.25), namely

5.2 $$\begin{aligned} \mathcal {L}_1^\textrm{in}[\phi ]=\mathcal {J}^{\textrm{in}}_1[\phi ,\psi ], \qquad \mathcal {L}_2^\textrm{in}[\psi ]=\mathcal {J}^{\textrm{in}}_2[\phi ,\psi ] \end{aligned}$$

with $\mathcal {L}_1^\textrm{in}, \mathcal {L}_2^\textrm{in}$ defined in (2.26) and $\mathcal {J}^{\textrm{in}}_1,\mathcal {J}^{\textrm{in}}_2$ in (2.27). There exists $\kappa _0$ big enough and a constant $M_7 > 0$ such that for $\kappa >\kappa _0$, equations (5.2) have solutions $(\phi ^{0,\textrm{u}},\psi ^{0,\textrm{u}}) \in \mathcal {X}_\times$ with $\Vert (\phi ^{0,\textrm{u}},\psi ^{0,\textrm{u}})\Vert _\times \le M_7$.

As in Sects. 3 and 4, the suitable right inverse of the linear operators $\mathcal {L}_1^\textrm{in},\mathcal {L}_2^\textrm{in}$ are given by the linear operators

5.3 $$\begin{aligned} \begin{aligned} \mathcal {G}_1^\textrm{in}[h](z)=&\,\dfrac{z^3}{5}\displaystyle \int _{-\infty }^z\dfrac{h(s)}{s^2}ds-\dfrac{1}{5z^2}\displaystyle \int _{-\infty }^z s^3h(s)ds\\ \mathcal {G}_2^\textrm{in}[h](z)=&\,\dfrac{1}{2i}\displaystyle \int _{-\infty }^z e^{-i(s-z)}h(s)ds-\dfrac{1}{2i}\displaystyle \int _{-\infty }^z e^{i(s-z)}h(s)ds. \end{aligned} \end{aligned}$$

The following lemma provides bounds for the linear operator $\mathcal {G}_{1,2}^{\textrm{in}}$. Its proof is straightforward from Proposition 5.2 in [31] (see also [4, 9, 12]).

#### Lemma 5.3

Consider $\kappa \ge 1$ big enough. Given $\ell > 2$, the operators $\mathcal {G}_1^\textrm{in}:\mathcal {X}_{\ell +2}\rightarrow \mathcal {X}_{\ell }$ and $\mathcal {G}_2^\textrm{in}:\mathcal {X}_{\ell }\rightarrow \mathcal {X}_{\ell }$ are well defined and the following statements hold.

1. $$\mathcal {G}_i^\textrm{in}\circ {\mathcal {L}}_i^\textrm{in}[h]={\mathcal {L}}_i^\textrm{in}\circ \mathcal {G}_i^\textrm{in}[h]=h$$, $$i=1,2$$.
2. For any $\ell >4$, there exists a constant $M>0$ independent of $\kappa$ such that, for every $h \in \mathcal {X}_{\ell }$,
  $$\begin{aligned} \begin{aligned} \left\| \mathcal {G}_1^\textrm{in}[h]\right\| _{\ell -2}&\le M\Vert h\Vert _{\ell },\\ \left\| \partial _z\mathcal {G}_1^\textrm{in}[h]\right\| _{\ell -1}&\le M\Vert h\Vert _{\ell }. \end{aligned} \end{aligned}$$
3. For any $\ell >1$, there exists a constant $M>0$ independent of $\kappa$ such that, for every $h \in \mathcal {X}_{\ell }$,
  $$\begin{aligned} \left\| \mathcal {G}_2^\textrm{in}[h]\right\| _{\ell }\le M\Vert h\Vert _{\ell }. \end{aligned}$$

We use the integral operators in (5.3) in order to obtain solutions of (5.2) with certain decay as $|z|\rightarrow \infty$ (within $D^{\star ,\textrm{in}}_{\theta ,\kappa }$, $\star =\textrm{u},\textrm{s}$). Indeed, such solutions must be fixed points of the operator

5.4 $$\begin{aligned} {\mathcal {F}}^\textrm{in}= \big ({\mathcal {G}}_1^\textrm{in}\circ \mathcal {J}^{\textrm{in}}_1,{\mathcal {G}}_2^\textrm{in}\circ \mathcal {J}^{\textrm{in}}_2 \big ), \end{aligned}$$

where the operators $\mathcal {J}^{\textrm{in}}_1, \mathcal {J}^{\textrm{in}}_2$ are those introduced in (2.27).

The following two lemmas give properties of the operator ${\mathcal {F}}^\textrm{in}$ when analyzed in the Banach space ${\mathcal {X}}_\times =\mathcal{D}\mathcal{X}_{3}\times \mathcal {X}_{5}$. The proofs of these two lemmas are straightforward using the definition of $\mathcal {J}^{\textrm{in}}_1$ and $\mathcal {J}^{\textrm{in}}_2$ in (2.27), see (5.4), and Lemmas 5.3 and 5.1.

#### Lemma 5.4

There exists a constant $b_3>0$ independent of $\kappa$ such that

$$\begin{aligned} \Vert {\mathcal {F}}^\textrm{in}[0,0]\Vert _\times \le b_3. \end{aligned}$$

#### Lemma 5.5

There exists $C_3>0$ such that for all $R>0$, if $(\phi ,\psi ),(\widetilde{\phi }, \widetilde{\psi })\in B(R)\subset {\mathcal {X}}_\times$, the operator ${\mathcal {F}}^\textrm{in}$ in (5.4) satisfies

$$\begin{aligned} \begin{aligned} \left\| {\mathcal {F}}_1^\textrm{in}[\phi ,\psi ]-{\mathcal {F}}_1^\textrm{in}[\widetilde{\phi },\widetilde{\psi }]\right\| _{3}&\le C_3\Vert \psi -\psi '\Vert _{5}+\frac{C}{\kappa ^2}\Vert (\phi ,\psi )-(\widetilde{\phi }, \widetilde{\psi })\Vert _\textrm{in},\\ \left\| \partial _z{\mathcal {F}}_1^\textrm{in}[\phi ,\psi ]-\partial _z{\mathcal {F}}_1^\textrm{in}[\widetilde{\phi }, \widetilde{\psi }]\right\| _{4}&\le C_3\Vert \psi -\psi '\Vert _{5}+\frac{C}{\kappa ^2}\Vert (\phi ,\psi )-(\widetilde{\phi },\widetilde{\psi })\Vert _\textrm{in},\\ \left\| {\mathcal {F}}_2^\textrm{in}[\phi ,\psi ]-{\mathcal {F}}_2^\textrm{in}[\widetilde{\phi }, \widetilde{\psi }]\right\| _{5}&\le \frac{C}{\kappa ^2}\Vert (\phi ,\psi )-(\widetilde{\phi }, \widetilde{\psi })\Vert _\textrm{in}, \end{aligned} \end{aligned}$$

for some constant $C=C(R)>0$ independent of $\kappa$.

We use again Theorem 2.14 to conclude the existence of a fixed point of $(\phi ,\psi )=\mathcal {F}^\textrm{in}[\phi ,\psi ]$ belonging to $B(3(C_3+1)b_3) \subset \mathcal {X}_\times$. This fixed point is the function given in item (1) of Theorem 2.8. Moreover, by construction it satisfies the stated estimates and they are real analytic functions. The symmetry is a consequence of the reversibility of equation (2.25) with respect to (2.28)

### The difference between the solutions of the inner equation

To complete the proof of Theorem 2.8, we analyze the differences

$$ \Delta \phi ^0(z)= \phi ^{0,\textrm{u}}(z)-\phi ^{0,\textrm{s}}(z),\qquad \Delta \psi ^0(z)= \psi ^{0,\textrm{u}}(z)-\psi ^{0,\textrm{s}}(z), $$

for $$z\in \mathcal {R}^{\textrm{in},+}_{\theta ,\kappa }$$ with

$$ \mathcal {R}^{\textrm{in},+}_{\theta ,\kappa }= D^{\textrm{u},\textrm{in}}_{\theta ,\kappa }\cap D^{\textrm{s}, \textrm{in}}_{\theta ,\kappa }\cap \{ z\in i{\mathbb {R}}\text { and }\Im (z)<0 \}. $$

Given a continuous function $f:\overline{\mathcal {R}^{\textrm{in},+}_{\theta ,\kappa }}\rightarrow {\mathbb {C}}$, we define the norm

$$\begin{aligned} \Vert f\Vert _{\ell ,\textrm{exp}}=\displaystyle \sup _{z\in \overline{\mathcal {R}^{\textrm{in},+}_{\theta ,\kappa }}}|z^{\ell }e^{iz} f(z)| \end{aligned}$$

and the Banach spaces

$$\begin{aligned} \begin{aligned} \mathcal {Z}_{\ell ,\textrm{exp}}&=\left\{ f: \overline{\mathcal {R}^{\textrm{in},+}_{\theta ,\kappa }}\rightarrow {\mathbb {C}};\text { continuous with } \Vert f\Vert _{\ell ,\textrm{exp}}<\infty \right\} ,\\ \mathcal{D}\mathcal{Z}_{\ell ,\textrm{exp}}&=\left\{ f: \overline{\mathcal {R}^{\textrm{in},+}_{\theta ,\kappa }}\rightarrow {\mathbb {C}};\text { continuous with } \Vert f\Vert _{\ell ,\textrm{exp}} +\Vert f'\Vert _{\ell ,\textrm{exp}}<\infty \right\} . \end{aligned} \end{aligned}$$

We will consider the product Banach space

$$\begin{aligned} \mathcal {Z}_{\times ,\textrm{exp}}=\mathcal{D}\mathcal{Z}_{0,\textrm{exp}}\times \mathcal {Z}_{0,\textrm{exp}} \end{aligned}$$

and denote by $\Vert \cdot \Vert _{\times ,\textrm{exp}}$ the associated norm:

$$\begin{aligned} \Vert (\phi ,\psi )\Vert _{\times ,\varepsilon } = \textrm{max}\{\Vert \phi \Vert _{0,\textrm{exp}} + \Vert \phi '\Vert _{0,\textrm{exp}}, \Vert \psi \Vert _{0,\textrm{exp}}\}. \end{aligned}$$

It can be easily seen that, if $f\in {\mathcal {X}}_{\ell _1}$ and $g\in {\mathcal {Z}}_{\ell _2,\textrm{exp}}$, then $fg\in {\mathcal {Z}}_{\ell _1+\ell _2,\textrm{exp}}$ and $\Vert fg\Vert _{\ell _1+\ell _2,\textrm{exp}}\le \Vert f\Vert _{\ell _1} \Vert g\Vert _{\ell _2,\textrm{exp}}$.

The second item in Theorem 2.8 can be rewritten as the following proposition, which will be proved in the rest of this section.

#### Proposition 5.6

There exist $\Theta \in \mathbb {R}$ and $\kappa _0, M_8>0$ such that for $\kappa >\kappa _0$, $\Delta \phi ^0, \Delta \psi ^0 \in \mathcal {D}\mathcal {Z}_{0,\textrm{exp}}$ and they satisfy

$$\begin{aligned} \Vert \Delta \phi ^0 +\Theta e^{-iz}\Vert _{1,\textrm{exp}} + \Vert \partial _z \Delta \phi ^0-i\Theta e^{-iz}\Vert _{1,\textrm{exp}}&\le M_8 |\Theta |, \\ \Vert \Delta \psi ^0 -\Theta e^{-iz}\Vert _{1,\textrm{exp}} + \Vert \partial _z \Delta \psi ^0+i \Theta e^{-iz}\Vert _{1,\textrm{exp}}&\le M_8 |\Theta |. \end{aligned}$$

Since both the stable and unstable solutions satisfy equation (5.2), applying the mean value theorem, one can see that the functions $\Delta \phi ^0$, $\Delta \psi ^0$ satisfy a linear homogeneous equation of the form

5.5 $$\begin{aligned} \left\{ \begin{array}{l} \widetilde{\mathcal {L}}_1^\textrm{in}\Delta \phi ^0 ={\mathcal {P}}_1[\Delta \phi ^0,\Delta \psi ^0], \\ {\mathcal {L}}_2^\textrm{in}\Delta \psi ^0 ={\mathcal {P}}_2[\Delta \phi ^0,\Delta \psi ^0 ], \end{array} \right. \end{aligned}$$

where $\widetilde{\mathcal {L}}_1^\textrm{in}=-\partial ^2_z$, ${\mathcal {L}}_2^\textrm{in}$ is the operator introduced in (2.26) and ${\mathcal {P}}_1$, ${\mathcal {P}}_2$ are defined by

5.6 $$\begin{aligned} \left\{ \begin{array}{l} {\mathcal {P}}_1[\Delta \phi ^0,\Delta \psi ^0](z) =a_{11}(z)\Delta \phi ^0(z)-\Delta \psi ^0(z), \vspace{0.2cm} \\ {\mathcal {P}}_2[\Delta \phi ^0,\Delta \psi ^0](z) = a_{21}(z)\Delta \phi ^0(z)+a_{22}(z)\Delta \psi ^0(z)+a_{23}(z)\partial _z\Delta \phi ^0(z), \end{array} \right. \end{aligned}$$

where, introducing $\Phi ^{0,\star }=(\phi ^{0,\star },\psi ^{0,\star })$, $\star =\textrm{u},\textrm{s}$ and defining *N* as the functional such that the operator $\mathcal {J}^{\textrm{in}}_2 [\phi ,\psi ]$ in (2.27) can be written as

$$\begin{aligned} \mathcal {J}^{\textrm{in}}_2[\phi ,\psi ]=N[\phi ,\psi , \partial _z\phi ], \end{aligned}$$

$a_{i,j}$ is defined as

$$\begin{aligned} \begin{aligned} a_{11}(z)=&-\frac{6}{z^2}+\int _{0}^1D_1 \mathcal {J}^{\textrm{in}}_1[\Phi ^{0,\textrm{s}}(z)+\sigma (\Phi ^{0,\textrm{u}}(z)-\Phi ^{0,\textrm{s}}(z)) ]d\sigma ,\\ a_{2j}(z)=&\int _{0}^1D_j N\big [\Phi ^{0,\textrm{s}}(z) +\sigma (\Phi ^{0,\textrm{u}}(z)-\Phi ^{0,\textrm{s}}(z)),\\  &\qquad \qquad \partial _z\phi ^{0,\textrm{s}}(z)+\sigma (\partial _z\phi ^{0,\textrm{u}}(z)-\partial _z\phi ^{0,\textrm{s}}(z))\big ]d\sigma . \end{aligned} \end{aligned}$$

Using the norm introduced in (5.1), these functions satisfy

5.7 $$\begin{aligned} \Vert a_{11}\Vert _2\lesssim 1,\quad \Vert a_{21}\Vert _4\lesssim 1,\quad \Vert a_{22}\Vert _2\lesssim 1,\quad \Vert a_{23}\Vert _3\lesssim 1. \end{aligned}$$

We now write equation (5.5) as an integral fixed point equation. On the one hand,

$$\begin{aligned} \partial _z \Delta \phi ^0 (z)= C_1 - \int _{z_1}^z \mathcal {P}_1[\Delta \phi ^0,\Delta \psi ^0](s) ds \end{aligned}$$

with $C_1= \partial _z \Delta \phi ^0(z_1)$. Since $\lim _{\Im z \rightarrow -\infty } \partial _z \Delta \phi ^0(z)=0$, we conclude that

$$\begin{aligned} \partial _z \Delta \phi ^0(z) = - \int _{-i\infty }^z \mathcal {P}_1[\Delta \phi ^0,\Delta \psi ^0](s) ds \end{aligned}$$

and as a consequence, reasoning analogously,

5.8 $$\begin{aligned} \Delta \phi ^0(z) = \int _{-i\infty }^{z} \int _{-i\infty }^s \mathcal {P}_1[\Delta \phi ^0,\Delta \psi ^0](\sigma ) d\sigma . \end{aligned}$$

On the other hand, recalling that $\mathcal {L}^\textrm{in}_2 [\Delta \psi ^0]= \partial _z^2 \Delta \psi ^0 + \Delta \psi ^0$, we have

$$\begin{aligned} \Delta \psi ^0(z)&= e^{iz} \left( C_1 + \frac{1}{2i }\int _{z_1}^z e^{-is} h(s) ds \right) + e^{-iz}\left( C_2- \int _{z_2}^z e^{is}h(s) ds\right) \end{aligned}$$

with

$$\begin{aligned} 2i e^{iz_1} C_1 = i \Delta \psi ^0(z_1) + \partial _z \Delta \psi ^0(z_1), \qquad 2i e^{-iz_2} C_2 = i \Delta \psi ^2(z_2) - \partial _z \Delta \psi ^0(z_2), \end{aligned}$$

Using (5.7), taking $z_2 =-i\kappa$ and imposing that $\lim _{\Im z \rightarrow -\infty } \Delta \psi ^0(z) =0$, we obtain

5.9 $$\begin{aligned} \begin{aligned} \Delta \psi ^0(z)=&\int _{-i\infty }^z \frac{e^{-i(s-z)}}{2i} {\mathcal {P}}_2[\Delta \phi ^0, \Delta \psi ^0](s) ds + \Theta _0e^{-iz} \\  &-\int _{-i\kappa }^z \frac{e^{i(s-z)}}{2i}{\mathcal {P}}_2[\Delta \phi ^0, \Delta \psi ^0](s) ds \end{aligned} \end{aligned}$$

with

5.10 $$\begin{aligned} \Theta _0 = \Theta _0(\kappa )=\frac{1}{2i} e^{\kappa } \big ( i \Delta \psi ^0 (-i\kappa )- \partial _z \Delta \psi ^0 (-i\kappa ) \big ). \end{aligned}$$

We emphasize that, from item (1) of Theorem 2.8, $|\Delta \phi ^0 (z)| \lesssim |z|^{-3}$, $|\Delta \psi ^0 (z)|\lesssim |z|^{-5}$ uniformly on the domain $\mathcal {R}_{\theta ,\kappa }^\textrm{in}$ and hence, using also bounds (5.7) of $a_{ij}$, the improper integrals in (5.8) and (5.9) are well defined. Therefore, $(\Delta \phi ^0, \Delta \psi ^0)$ satisfies the fixed point equation

5.11 $$\begin{aligned} \left\{ \begin{array}{l} \Delta \phi ^0(z) =\widetilde{\mathcal {G}}^\textrm{in}_1 \circ {\mathcal {P}}_1[\Delta \phi ^0,\Delta \psi ^0](z),\\ \Delta \psi ^0(z) =\Theta _0 e^{-iz}+ \widetilde{\mathcal {G}}^\textrm{in}_2\circ {\mathcal {P}}_2[\Delta \phi ^0,\Delta \psi ^0](z). \end{array} \right. \end{aligned}$$

where the constant $\Theta _0 = \Theta _0(\kappa )$ is defined in (5.10), ${\mathcal {P}}$ in (5.6) and $\widetilde{\mathcal {G}}^\textrm{in}=(\widetilde{\mathcal {G}}^\textrm{in}_!,\widetilde{\mathcal {G}}^\textrm{in}_2)$ is the integral linear operator defined on functions $h:\mathcal {R}^{\textrm{in},+}_{\theta ,\kappa }\rightarrow {\mathbb {C}}$, as

$$\begin{aligned} \begin{aligned} \widetilde{\mathcal {G}}^\textrm{in}_{1}[h](z)&=-\int _{-i\infty }^z\int _{-i\infty }^s h(\sigma )d\sigma \, d s,\\ \widetilde{\mathcal {G}}^\textrm{in}_{2}[h](z)&=\displaystyle \int _{-i\infty }^z\dfrac{e^{-i(s-z)}h(s)}{2i}ds -\displaystyle \int _{-i\kappa }^z\dfrac{e^{i(s-z)}h(s)}{2i}ds. \end{aligned} \end{aligned}$$

Denoting $\Delta \Phi ^0 = (\Delta \phi ^0, \Delta \psi ^0)$, equation (5.11) can be rewritten as

$$\begin{aligned} \Delta \Phi ^0 = \Delta \Phi ^0_0+ \widetilde{\mathcal {P}}[ \Delta \Phi ^0], \qquad \Delta \Phi ^0_0(z) = \begin{pmatrix} 0 \\ \Theta _0 e^{-iz} \end{pmatrix}, \end{aligned}$$

where $\widetilde{\mathcal {P}}$ is the linear operator defined by

5.12 $$\begin{aligned} \widetilde{\mathcal {P}}=\big (\widetilde{\mathcal {P}}_1, \widetilde{\mathcal {P}}_2\big )=\big (\widetilde{\mathcal {G}}^\textrm{in}_1\circ {\mathcal {P}}_1, \widetilde{\mathcal {G}}^\textrm{in}_2\circ \mathcal {P}_2\big ). \end{aligned}$$

Notice that, if the operator $\textrm{Id} - \widetilde{\mathcal {P}}$ were invertible, then we could write $\Delta \Phi ^0 = \big ( \textrm{Id} - \widetilde{\mathcal {P}}\big )^{-1} [\Delta \Phi ^0_0]$ and study $\Delta \Phi ^0$ through $\widetilde{\mathcal {P}}$ and $\Delta \Phi ^0$.

The following lemma specifies properties of the linear operator $\widetilde{\mathcal {P}}$. Its proof is straightforward using the estimates in (5.7) and the definition of the operators in (5.12), where we also recall that $\mathcal {R}_{\theta ,\kappa }^\textrm{in}$ is a subset of $i\mathbb {R}$.

#### Lemma 5.7

The linear operator $\widetilde{\mathcal {P}}:\mathcal {Z}_{\times ,\textrm{exp}}\rightarrow \mathcal {Z}_{\times ,\textrm{exp}}$ given in (5.12), is well defined. Moreover, there exists a constant *M* such that for each $\kappa \ge 1$,

1. The linear operators $\widetilde{{\mathcal {P}}}_1, \partial _z\widetilde{{\mathcal {P}}}_1:\mathcal {Z}_{\times ,\textrm{exp}}\rightarrow \mathcal {Z}_{0,\textrm{exp}}$ satisfy
  $$\begin{aligned} \begin{aligned} \Vert \widetilde{{\mathcal {P}}}_1[\Delta \phi ^0,\Delta \psi ^0] \Vert _{0,\textrm{exp}}&\le \frac{M}{\kappa ^2}\Vert \Delta \phi ^0\Vert _{0,\textrm{exp}} +M\Vert \Delta \psi ^0\Vert _{0,\textrm{exp}}, \\ \Vert \partial _z \widetilde{{\mathcal {P}}}_1[\Delta \phi ^0,\Delta \psi ^0] \Vert _{0,\textrm{exp}}&\le \frac{M}{\kappa ^2}\Vert \Delta \phi ^0\Vert _{0,\textrm{exp}} +M\Vert \Delta \psi ^0\Vert _{0,\textrm{exp}}. \end{aligned} \end{aligned}$$
2. The linear operator $\widetilde{{\mathcal {P}}}_2:\mathcal {Z}_{\times ,\textrm{exp}}\rightarrow \mathcal {Z}_{0,\textrm{exp}}$ satisfy
  $$\begin{aligned} \Vert \widetilde{{\mathcal {P}}}_2[\Delta \phi ^0,\Delta \psi ^0]\Vert _{0,\textrm{exp}}\le \dfrac{M}{ \kappa }\Vert (\Delta \phi ^0,\Delta \psi ^0) \Vert _{0,\textrm{exp}}. \end{aligned}$$

This result of Lemma 5.7 does not lead to check that $\widetilde{\mathcal {P}}$ has small norm so that $\textrm{Id} - \widetilde{\mathcal {P}}$ is invertible. Hence we proceed in a similar way as in the proof of Theorem 2.14. We emphasize that $\Delta \Phi _0$ is also a solution of

5.13 $$\begin{aligned} \Delta \Phi ^0 = \widehat{\Delta \Phi ^0_0}+ \widehat{\mathcal {P}}[ \Delta \Phi ^0], \qquad \widehat{\Delta \Phi ^0_0}(z) = \Delta \Phi _0^0 (z)+ \begin{pmatrix} \widetilde{\mathcal {P}}_1[\Delta \Phi ^0_0] \\ 0 \end{pmatrix}, \end{aligned}$$

where $\widehat{\mathcal {P}}$ is the linear operator defined by

$$\begin{aligned} \left\{ \begin{array}{l} \widehat{\mathcal {P}}_1[\Delta \phi ^0, \Delta \psi ^0] =\widetilde{\mathcal {P}}_1 \big [\Delta \phi ^0, \widetilde{\mathcal {P}}_2[\Delta \phi ^0, \Delta \psi ^0 ]\big ], \vspace{0.2cm}\\ \widehat{\mathcal {P}}_2[\Delta \phi ^0, \Delta \psi ^0 ] = \widetilde{\mathcal {P}}_2[\Delta \phi ^0 \Delta \psi ^0 ]. \end{array} \right. \end{aligned}$$

Lemma 5.7 implies that $\widehat{\mathcal {P}}$ satisfies

$$\begin{aligned} \left\| \widehat{\mathcal {P}}[\Delta \phi ^0,\Delta \psi ^0]\right\| _{\times ,\textrm{exp}}\lesssim \frac{1}{\kappa }\left\| \Delta \phi ^0,\Delta \psi ^0\right\| _{\times ,\textrm{exp}}. \end{aligned}$$

Then we conclude that, taking $\kappa$ big enough, $\textrm{Id}-\widehat{\mathcal {P}}$ is invertible in ${\mathcal {Z}}_{\times ,\textrm{exp}}$. On the other hand, using that $\Delta _0^0(z) = (0, \Theta _0 e^{-iz})^\top$, formula (5.6) of ${\mathcal {P}}_1$ and that $\widetilde{\mathcal {P}}_1 = \widetilde{\mathcal {G}}^\textrm{in}_1\circ {\mathcal {P}}_1$, we obtain that

5.14 $$\begin{aligned} \widehat{\Delta \Phi _0^0}(z)=\begin{pmatrix} \widetilde{\mathcal {P}}_1[\Delta \Phi _0^0](z) \\ \Theta _0 e^{-iz} \end{pmatrix}= \begin{pmatrix} -\Theta _0 e^{-iz} \\ \Theta _0 e^{-iz} \end{pmatrix} \in {\mathcal {Z}}_{\times ,\textrm{exp}}. \end{aligned}$$

As a consequence, it follows from equation (5.13) that $\big ( \textrm{Id} - \widehat{\mathcal {P}}\big ) \Delta \Phi ^0 = \widehat{\Delta \Phi _0^0} \in {\mathcal {Z}}_{\times ,\textrm{exp}}$ and we conclude

$$\begin{aligned} \Delta \Phi ^0 = \big ( \textrm{Id} - \widehat{\mathcal {P}}\big )^{-1}[ \widehat{\Delta \Phi ^0_0}] \in {\mathcal {Z}}_{\times ,\textrm{exp}}. \end{aligned}$$

In addition, this implies that, for $z\in \mathcal {R}^{\textrm{in},+}_{\theta ,\kappa }$,

$$\begin{aligned} \begin{pmatrix} \Delta \phi ^0(z)\\ \Delta \psi ^0(z)\end{pmatrix} = \Theta _0 e^{-iz}\begin{pmatrix}-1+{\mathcal {O}}\left( \frac{1}{ {\kappa }}\right) \\ 1+{\mathcal {O}}\left( \frac{1}{\kappa }\right) \end{pmatrix}. \end{aligned}$$

Note that this asymptotic formula is not the one given in Proposition 5.6. Indeed, the asymptotics here is given with respect to $\kappa ^{-1}$ whereas the one in Proposition 5.6 is given in terms of $z^{-1}$. To improve the asymptotics, we need to define a new constant $\Theta$ which is $\kappa ^{-1}$ close to $\Theta _0$.

We define the constant

5.15 $$\begin{aligned} \Theta =\Theta _0-\int _{-i\kappa }^{-i\infty }\dfrac{e^{iz}{\mathcal {P}}_2[\Delta \phi ^0,\Delta \psi ^0] (z)}{2i}dz. \end{aligned}$$

Note that the fact that $(\Delta \phi ^0,\Delta \psi ^0)\in {\mathcal {Z}}_{\times ,\textrm{exp}}$ implies that the integral is convergent and the constant $\Theta$ is well-defined.

Proposition 5.6 (and hence the second statement of Theorem 2.8) is a direct consequence of the following lemma.

#### Lemma 5.8

The functions $(\Delta \phi ^0,\Delta \psi ^0)$ satisfy that, for $z\in \mathcal {R}^{\textrm{in},+}_{\theta ,\kappa }$,

$$\begin{aligned} \begin{pmatrix} \Delta \phi ^0(z)\\ \Delta \psi ^0(z)\end{pmatrix} = \Theta e^{-iz}\begin{pmatrix}-1+{\mathcal {O}}\left( \frac{1}{z}\right) \\ 1+{\mathcal {O}}\left( \frac{1}{z}\right) \end{pmatrix}, \end{aligned}$$

for some constant $\Theta \in \mathbb {R}$.

#### Proof

We exploit the fact that we already have proven that $(\Delta \phi ^0, \Delta \psi ^0) \in {\mathcal {Z}}_{\times ,\textrm{exp}}$. We obtain the asymptotic formula for each component. From (5.11) and using definition (5.15) of $\Theta$, we note that, the second component can be written as

$$\begin{aligned} \Delta \psi ^0(z)=\Theta e^{-iz}+\check{{\mathcal {G}}}_2^\textrm{in}\big [{\mathcal {P}}_2[\Delta \phi ^0,\Delta \psi ^0]\big ](z), \end{aligned}$$

with

$$\begin{aligned} \check{{\mathcal {G}}}^{\textrm{in}}_2[h](z)=\displaystyle \int _{-i\infty }^z\dfrac{e^{-i(s-z)}h(s)}{2i}ds -\displaystyle \int _{-i\infty }^z\dfrac{e^{i(s-z)}h(s)}{2i}ds. \end{aligned}$$

Since $(\Delta \phi ^0,\Delta \psi ^0)\in {\mathcal {Z}}_{\times ,\textrm{exp}}$, estimates (5.7) imply that ${\mathcal {P}}_2[\Delta \phi ^0,\Delta \psi ^0] \in {\mathcal {Z}}_{2, \textrm{exp}}$ and

$$\begin{aligned} \left\| {\mathcal {P}}_2[\Delta \phi ^0,\Delta \psi ^0] \right\| _{2,\textrm{exp}}\lesssim 1. \end{aligned}$$

Then, it is a straightforward computation to see that $\Delta \psi ^0-\Theta e^{-iz}\in {\mathcal {Z}}_{1,\textrm{exp}}$ and

$$\begin{aligned} \left\| \Delta \psi ^0-\Theta e^{-iz} \right\| _{1,\textrm{exp}}=\left\| \check{{\mathcal {G}}}_2^\textrm{in}\big [{\mathcal {P}}_2[\Delta \phi ^0,\Delta \psi ^0]\big ]\right\| _{1,\textrm{exp}}\lesssim 1. \end{aligned}$$

This completes the proof of the asymptotic formula for $\Delta \psi ^0$. Analogous computations lead to the asymptotic formula for $\partial _z \Delta \psi ^0$.

Now we prove the asymptotic formula for the first component. To this end, using that we rewrite the identity (see (5.13) and (5.14))

$$\begin{aligned} \Delta \phi ^0(z)=\widetilde{\mathcal {P}}_1[\Delta \Phi _0^0](z) + \widehat{\mathcal {P}}_1[\Delta \phi ^0,\Delta \psi ^0](z)=-\Theta _0e^{-iz}+\widetilde{\mathcal {P}}_1\big [\Delta \phi ^0,\widetilde{\mathcal {P}}_2[\Delta \phi ^0,\Delta \psi ^0]\big ](z) \end{aligned}$$

as

$$\begin{aligned} \Delta \phi ^0(z)=-\Theta e^{-iz}+\widetilde{\mathcal {P}}_1\big [\Delta \phi ^0,\check{{\mathcal {G}}}_2^\textrm{in}\big [{\mathcal {P}}_2[\Delta \phi ^0,\Delta \psi ^0]\big ]\big ](z), \end{aligned}$$

where we have used

$$\begin{aligned} \Delta \psi _0 (z) = \Theta _0 e^{-iz} + \widetilde{\mathcal {P}}_2[\Delta \phi ^0,\Delta \psi ^0 ] (z)= \Theta e^{-iz} + \check{{\mathcal {G}}}_2^\textrm{in}\big [{\mathcal {P}}_2[\Delta \phi ^0,\Delta \psi ^0] \big ] (z). \end{aligned}$$

Then, it can be easily seen that

$$\begin{aligned} \Delta \phi ^0(z)+\Theta e^{-iz}=\widetilde{\mathcal {P}}_1\big [\Delta \phi ^0,\check{{\mathcal {G}}}_2^\textrm{in}\big [{\mathcal {P}}_2[\Delta \phi ^0,\Delta \psi ^0]\big ]\big ]\in {\mathcal {Z}}_{1,\textrm{exp}} \end{aligned}$$

and

$$\begin{aligned} \left\| \Delta \phi ^0+\Theta e^{-iz}\right\| _{1,\textrm{exp}}\lesssim 1. \end{aligned}$$

This completes the asymptotic formula for the first component and analogously we have the one for its derivative.

It only remains to show that the constant $\Theta$ is real. This is a direct consequence of the fact that the solutions $(\phi ^{0,\star }, \psi ^{0,\star })$, $\star =\textrm{u},\textrm{s}$ are real-analytic and satisfy (2.30). Indeed these two properties imply that, for $z\in \mathcal {R}^{\textrm{in},+}_{\theta ,\kappa }$ (recall that $\mathcal {R}^{\textrm{in},+}_{\theta ,\kappa }\subset i{\mathbb {R}}$),

$$\begin{aligned} \Delta \psi ^0(z)\in {\mathbb {R}}. \end{aligned}$$

This implies that $e^{iz}\Delta \psi ^0(z)\in {\mathbb {R}}$ and therefore $\Theta \in {\mathbb {R}}$ since it can be defined as

$$\begin{aligned} \lim _{\Im z\rightarrow -\infty , z\in i{\mathbb {R}}}e^{iz}\Delta \psi ^0(z). \end{aligned}$$

This completes the proof of Lemma 5.8. $\square$

Finally, the fact that $\Theta \ne 0$ if and only if $\Delta \phi ^0$ does not vanish at one point is a direct consequence of the asymptotic formula. This proves the third item of Theorem 2.8.

## Matching Around Singularities

Here we prove Theorem 2.10. We will give the proof only for the − case, being the $+$ case is analogous. Due to this reason, we omit the sign ± in our notation and we provide estimates for $(\xi ^{\textrm{u}},\eta ^{\textrm{u}})$ and $(\xi ^\textrm{aux},\eta ^\textrm{aux})$ around the singularity $x_-$.

It is convenient to work with inner variables, see (2.23) and (2.24), namely,

6.1 $$\begin{aligned} z=\varepsilon ^{-1} (x-x_-), \quad \phi (z)=\frac{\varepsilon }{c_{- 1}} \xi (x_-+\varepsilon z ),\quad \psi (z)=\frac{\varepsilon ^3}{c_{- 1}} \eta (x_-+ \varepsilon z). \end{aligned}$$

We define now the matching domain $D^{-,\textrm{match}}_{\theta _1,\theta _2,\nu }$ by (2.32) in the inner variable. We fix $0<\nu <1$ and $0<\theta _2< \theta<\theta _1<\frac{\pi }{2}$, where $\theta$ is the angle introduced in (2.7), and we define

$$\begin{aligned} \mathcal {D}_{\theta _1,\theta _2,\nu }^{\textrm{match}} = \widehat{-i\kappa , z_1,z_2}, \end{aligned}$$

the open triangle with vertices $-i\kappa , z_1,z_2$, with

$$\begin{aligned} z_1=-i\kappa +\frac{1}{\varepsilon ^{1-\nu }} e^{-i \theta _1}, \qquad z_2 = -i\kappa - \frac{1}{\varepsilon ^{1-\nu }} e^{-i\theta _2}. \end{aligned}$$

In addition, if we define

$$\begin{aligned} \hat{u}_0(z) = u_0(x_-+\varepsilon z), \end{aligned}$$

we notice that, if $z \in \mathcal {D}_{\theta _1,\theta _2}^{\nu , \textrm{match}}$, then $|\varepsilon z| \lesssim \varepsilon ^{\nu }$ and therefore

6.2 $$\begin{aligned} \begin{aligned} \varepsilon c_{-1}^{-1} \hat{u}_0(z)&= \frac{1}{z} + \varepsilon \sum _{k\ge 0} c_k (\varepsilon z)^k= \frac{1}{z} + \mathcal {O}(\varepsilon ), \\ \varepsilon c_{-1}^{-1} \hat{u}'_0(z)&= - \frac{1}{z^2 } +\mathcal {O}(\varepsilon ^2). \end{aligned} \end{aligned}$$

Moreover, defining

6.3 $$\begin{aligned} \phi ^{\star }(z) = \frac{\varepsilon }{c_{-1}} \xi ^{\star } (x_-+\varepsilon z ), \qquad \psi ^{\star }(z) = \frac{\varepsilon ^3}{c_{-1}} \eta ^{\star } (x_-+\varepsilon z ), \qquad \star =\textrm{u},\textrm{aux}\end{aligned}$$

with $(\xi ^{\textrm{u}}, \eta ^{\textrm{u}})$ and $(\xi ^\textrm{aux}, \eta ^\textrm{aux})$, given in Theorems 2.2 and 2.3 respectively, we have that

6.4 $$\begin{aligned} \big |\phi ^\star (z) \big | \lesssim \frac{1}{|z|^3}, \qquad \big |\partial _z \phi ^\star (z) \big | \lesssim \frac{1}{|z|^4}, \qquad \big | \psi ^\star (z) \big |\lesssim \frac{1}{|z|^5}. \end{aligned}$$

Now we rephrase Theorem 2.10 in the inner variables as follows.

### Theorem 6.1

Let $\theta >0, \kappa _0$ be fixed as in Theorems 2.2, 2.3 and 2.8. Take $0<\theta _2<\theta<\theta _1< \frac{\pi }{2}$ and $\nu \in (0,1)$. We introduce the functions

$$\begin{aligned} \delta \phi ^{\star }(z) = \frac{\varepsilon }{ c_{-1}} \delta \xi ^\star _-( x_-+\varepsilon z ), \quad \delta \psi ^{\star }(z) = \frac{\varepsilon ^3 }{ c_{-1}} \delta \eta ^\star _-( x_-+\varepsilon z), \quad \star =\textrm{u},\textrm{aux}, \end{aligned}$$

with $\delta \xi ^\star _-,\delta \eta ^\star _-$ defined in Theorem 2.10. Then there exist $\kappa _1\ge \kappa _0$ and a constant $M>0$ such that for all $\kappa \ge \kappa _1$ and $z \in \mathcal {D}_{\theta _1,\theta _2, \nu }^{\textrm{match}}$

$$\begin{aligned}&\big |\delta \phi ^\star (z)\big | \le M |\log \varepsilon |\frac{\varepsilon ^{1-\nu }}{|z|^2},&\big | \partial _ z \delta \phi ^\star (x)\big | \le M |\log \varepsilon |\frac{\varepsilon ^{1-\nu }}{|z|^3},\\&\big |\delta \eta ^\star (x)\big | \le M |\log \varepsilon |\frac{\varepsilon ^{1-\nu }}{|z|^4},&\big | \partial _z \delta \eta ^\star _- (x)\big | \le M |\log \varepsilon |\frac{\varepsilon ^{1-\nu }}{|z|^4}. \end{aligned}$$

### Remark 6.2

We emphasize that we already know the existence of $\delta \phi ^\star , \delta \psi ^\star$ in the matching domain and that, using (6.4) and Theorem 2.8

$$\begin{aligned} \big |\delta \phi ^\star (z) \big | \le \big | \phi ^\star (z)\big | + \big | \phi ^{0,\star }(z) \big | \lesssim \frac{1}{|z|^3}, \qquad \big | \delta \psi ^\star (z) \big | \le \big | \phi ^\star (z)\big | + \big | \phi ^{0,\star }(z) \big |\lesssim \frac{1}{|z|^5}, \end{aligned}$$

and also $\big | \partial _z \delta \phi ^\star \big | \lesssim |z|^{-4}$. However, these estimates do not imply that, when $\varepsilon =0$, $\delta \phi ^\star , \delta \psi ^\star =0$.

The remaining part of this section is devoted to prove Theorem 6.1. The prove for $\star =\textrm{u}, \textrm{aux}$ are identical and, therefore, we only present the first one.

### Reformulation of the problem

To prove Theorem 6.1 we look for differential equations which have ($\delta \xi ^\textrm{u}, \delta \eta ^\textrm{u})$, as a solutions. To this end, let ($\xi ^\textrm{u}, \eta ^\textrm{u})$ be the solution of equation (2.3) provided in Theorem 2.2 and consider the function $(\phi ^\textrm{u}, \psi ^\textrm{u})$ defined in (6.3). Applying the change of coordinates to equation (2.3) we have that

$$\begin{aligned} \left\{ \begin{array}{l} \mathcal {L}^{\textrm{in}}_1[\phi ^\textrm{u}] = \mathcal {J}^{\textrm{match}}_1[\phi ^\textrm{u},\psi ^\textrm{u};\varepsilon ]:=\mathcal {J}^{\textrm{in}}_1[\phi ^\textrm{u},\psi ^\textrm{u}] + \mathcal {A}_1[\phi ^\textrm{u}, \psi ^\textrm{u};\varepsilon ], \vspace{0.2cm} \\ \mathcal {L}^{\textrm{in}}_2[\psi ^\textrm{u}] = \mathcal {J}^{\textrm{match}}_2[\phi ^\textrm{u},\psi ^\textrm{u};\varepsilon ]:=\mathcal {J}^{\textrm{in}}_2[\phi ^\textrm{u},\psi ^\textrm{u}] + \mathcal {A}_2[\phi ^\textrm{u}, \psi ^\textrm{u};\varepsilon ], \end{array} \right. \end{aligned}$$

where ${\mathcal {L}}_j^\textrm{in}$ and $\mathcal {J}^{\textrm{in}}_j$, $j=1,2$ are introduced in (2.26) and (2.27).

We introduce the notation $\Phi =(\phi ,\psi )$, $\mathcal {A}[\Phi ;\varepsilon ]=(\mathcal {A}_1[\Phi ;\varepsilon ], \mathcal {A}_2[\Phi ;\varepsilon ])$,

$$\begin{aligned} \mathcal {L}^{\textrm{in}} [\Phi ] = (\mathcal {L}^{\textrm{in}}_1[\phi ], \mathcal {L}^{\textrm{in}}_2[\psi ]), \qquad \mathcal {J}^{\textrm{in}}[\Phi ] = (\mathcal {J}^{\textrm{in}}_1[\Phi ], \mathcal {J}^{\textrm{in}}_2[\Phi ]), \end{aligned}$$

and

$$\begin{aligned} \mathcal {J}^{\textrm{match}}[\Phi ;\varepsilon ] = (\mathcal {J}^{\textrm{match}}_1[\Phi ;\varepsilon ], \mathcal {J}^{\textrm{match}}_2[\Phi ;\varepsilon ]) = \mathcal {J}^{\textrm{in}}[\Phi ]+ \mathcal {A}[\Phi ;\varepsilon ]. \end{aligned}$$

Since, by Theorem 2.8, $\Phi ^{0,\textrm{u}}=(\phi ^{0,\textrm{u}},\psi ^{0,\textrm{u}})$ is a solution of $\mathcal {L}^{\textrm{in}} [\Phi ^{0,\textrm{u}}] = \mathcal {J}^{\textrm{in}}[\Phi ^{0,\textrm{u}}]$ and $\Phi ^{\textrm{u}}$ satisfies $\mathcal {L}^{\textrm{in}}[\Phi ^{\textrm{u}}] = \mathcal {J}^{\textrm{in}}[\Phi ^{\textrm{u}}] + \mathcal {A}[\Phi ^{\textrm{u}};\varepsilon ]$, using the mean value theorem, we have that $\delta \Phi ^\textrm{u}=\Phi ^{\textrm{u}} - \Phi ^{0,\textrm{u}}$ satisfies

$$\begin{aligned} \mathcal {L}^{\textrm{in}}[\delta \Phi ^\textrm{u}]=&\mathcal {L}^{\textrm{in}}[\Phi ^{\textrm{u}}](z) - \mathcal {L}^{\textrm{in}}[\Phi ^{0,\textrm{u}}](z)\\ =&\int _{0}^1 D_{\Phi }\mathcal {J}^{\textrm{in}}[\Phi ^{0,\textrm{u}} + \lambda (\Phi ^{\textrm{u}} - \Phi ^{0,\textrm{u}})] (z) \cdot (\Phi ^{\textrm{u}}(z) - \Phi ^{0,\textrm{u}}(z)) \, d\lambda + \mathcal {A}[\Phi ^{\textrm{u}};\varepsilon ](z) \\&+ \int _{0}^1 D_{\partial _z \phi } \mathcal {J}^{\textrm{in}}[\Phi ^{0,\textrm{u}} + \lambda (\Phi ^{\textrm{u}} - \Phi ^{0,\textrm{u}})](z)\cdot \left( \partial _z \phi ^{\textrm{u}} (z) - \partial _z \phi ^{0,\textrm{u}} (z) \right) \, d\lambda . \end{aligned}$$

We denote

6.5 $$\begin{aligned} \begin{aligned} \mathcal {B}_1^\textrm{u}(z)&= \int _{0}^1 D_{\Phi } \mathcal {J}^{\textrm{in}}[\Phi ^{0,\textrm{u}} + \lambda (\Phi ^{\textrm{u}} - \Phi ^{0,\textrm{u}})] (z) \, d\lambda - \left( \begin{array}{cc} 0 &  -1 \\ 0 &  0 \end{array}\right) , \\ \mathcal {B}_2^\textrm{u}(z)&= \int _{0}^1 D_{\partial _z \phi } \mathcal {J}^{\textrm{in}}[\Phi ^{0,\textrm{u}} + \lambda (\Phi ^{\textrm{u}} - \Phi ^{0,\textrm{u}})] (z) \, d\lambda , \\ \mathcal {B}_3(z)&= \left( \begin{array}{cc} 0 &  -1 \\ 0 &  0 \end{array}\right) , \end{aligned} \end{aligned}$$

and $A^\textrm{u}(z) = \mathcal {A}[\Phi ^\textrm{u};\varepsilon ](z)$. We emphasize that $\mathcal {B}_1^\textrm{u}, \mathcal {B}_2^\textrm{u}$ and $A^\textrm{u}$ are known functions that depend on the solutions $\Phi ^\textrm{u}=(\phi ^\textrm{u},\psi ^\textrm{u})$ and $\Phi ^{0,\textrm{u}}=(\phi ^{0,\textrm{u}}, \psi ^{0,\textrm{u}})$, which have already been constructed above. We then obtain that $\delta \Phi ^\textrm{u}= (\delta \phi ^\textrm{u},\delta \psi ^\textrm{u})$ satisfies the non-homogeneous linear equation

6.6 $$\begin{aligned} \mathcal {L}^{\textrm{in}} [\delta \Phi ^\textrm{u}] (z)= \mathcal {B}_1^\textrm{u}(z) \delta \Phi ^\textrm{u}(z) + \mathcal {B}_2^\textrm{u}(z) \partial _z \delta \phi ^\textrm{u}(z) + \mathcal {B}_3(z) \delta \Phi ^\textrm{u}(z) + A^\textrm{u}(z). \end{aligned}$$

The following lemma characterizes the solutions of $\mathcal {L}^\textrm{in}[\Phi ] = h$ with given initial conditions. Its proof is straightforward and is omitted.

#### Lemma 6.3

Let $\Phi$ be a solution of $\mathcal {L}^\textrm{in}[\Phi ]= h$ defined in $\mathcal {D}^{\textrm{match}}_{\theta _1,\theta _2,\nu }$. Then, $\Phi =(\phi ,\psi )$ is given by

$$ \Phi (z) = \left( \begin{array}{cc} z^3 a_\phi + \frac{1}{z^2} b_\phi \\ e^{i(z-z_1)} a_\psi + e^{-i(z-z_2)} b_\psi \end{array}\right) + {\mathcal {G}}^\textrm{match}[h], $$

where

6.7 $$\begin{aligned} \begin{aligned} a_\phi&= \frac{1}{ 5 z_1^3 } \big ( 2 \delta \phi (z_1)+ \partial _z \delta \phi (z_1) z_1\big ),&b_\phi&= \frac{z_1^2}{5} \big (3 \delta \phi (z_1)- \partial _z \delta \phi (z_1) z_1\big ), \\ a_\psi&= \frac{1}{2} \big (\delta \psi (z_1) - i \partial _z \delta \psi (z_1)\big ),&b_\psi&= \frac{1}{2} \big (\delta \psi (z_2) + i \partial _z \delta \psi (z_2)\big ), \end{aligned} \end{aligned}$$

and ${\mathcal {G}}^\textrm{match}[h]= ({\mathcal {G}}^\textrm{match}_1[h_1], {\mathcal {G}}^\textrm{match}_2[h_2] )$ is the linear operator (compare with (5.3)) defined by

6.8 $$\begin{aligned} \begin{aligned} {\mathcal {G}}^\textrm{match}_1 [h](z)&= \frac{z^3}{5} \int ^{z}_{z_1} \frac{h(s)}{s^2} ds- \frac{1}{5z^2} \int _{z_1}^z s^3 h(s) ds, \\ {\mathcal {G}}^\textrm{match}_2 [h](z)&=\frac{1}{2i} \int _{z_1}^z e^{-i(s-z)}h(s) ds - \frac{1}{2i}\int _{z_2}^z e^{i(s-z)} h(s) ds. \end{aligned} \end{aligned}$$

Since $\delta \Phi ^\textrm{u}$ is a solution of (6.6), Lemma 6.3 implies that $\delta \Phi ^\textrm{u}$ satisfies the following fixed point (affine) equation

6.9 $$\begin{aligned} \delta \Phi ^\textrm{u}(z) =&\left( \begin{array}{cc} z^3 a_{\phi ^\textrm{u}} + \frac{1}{z^2} b_{\phi ^\textrm{u}} \\ e^{i(z-z_1)} a_{\psi ^\textrm{u}} + e^{-i(z-z_2)} b_{\psi ^\textrm{u}} \end{array}\right) + {\mathcal {G}}^\textrm{match}[A^\textrm{u}](z) - \left( \begin{array}{c} {\mathcal {G}}^\textrm{match}_1[\delta \psi ^\textrm{u}](z) \\ 0\end{array}\right) \\  &+ {\mathcal {G}}^\textrm{match}[\mathcal {B}_1^\textrm{u}\cdot \delta \Phi ^\textrm{u}] (z) + {\mathcal {G}}^\textrm{match}[\mathcal {B}_2^\textrm{u}\cdot \partial _z \delta \Phi ^\textrm{u}](z), \nonumber \end{aligned}$$

where $a_{\phi ^\textrm{u}},b_{\phi ^\textrm{u}},a_{\psi ^\textrm{u}}, b_{\psi ^\textrm{u}}$ are defined by (6.7) and we have used definition (6.5) of $\mathcal {B}_3$. To shorten the notation we introduce

6.10 $$\begin{aligned} \begin{aligned} \delta \Phi _0^\textrm{u}(z)&= \begin{pmatrix}\delta \phi _0^\textrm{u}(z)\\ \delta \psi _0^\textrm{u}(z)\end{pmatrix} = \left( \begin{array}{cc} z^3 a_{\phi ^\textrm{u}} + \frac{1}{z^2} b_{\phi ^\textrm{u}} \\ e^{i(z-z_1)} a_{\psi ^\textrm{u}} + e^{-i(z-z_2)} b_{\psi ^\textrm{u}} \end{array}\right) + {\mathcal {G}}^\textrm{match}[A^\textrm{u}](z), \\ \mathcal {F}^{\textrm{match}}[\delta \Phi ]&= \begin{pmatrix}\mathcal {F}^{\textrm{match}}_1 [\delta \Phi ]\\ \mathcal {F}^{\textrm{match}}_2 [\delta \Phi ]\end{pmatrix} = {\mathcal {G}}^\textrm{match}[\mathcal {B}_1^\textrm{u}\cdot \delta \Phi ] (z) + {\mathcal {G}}^\textrm{match}[\mathcal {B}_2^\textrm{u}\cdot \partial _z \delta \Phi ](z), \end{aligned} \end{aligned}$$

after which we rewrite equation (6.9) as

6.11 $$\begin{aligned} \delta \Phi ^\textrm{u}= \delta \Phi ^\textrm{u}_0 - \left( \begin{array}{c} {\mathcal {G}}^\textrm{match}_1[\delta \psi ^\textrm{u}](z) \\ 0\end{array}\right) + \mathcal {F}^{\textrm{match}}[\delta \Phi ^\textrm{u}]. \end{aligned}$$

Using that $\delta \Phi ^\textrm{u}$ is a solution of (6.11), we observe that $\delta \Phi ^\textrm{u}$ must be also a solution of

6.12 $$\begin{aligned} \delta \Phi ^\textrm{u}= \widehat{\delta \Phi ^\textrm{u}_0} + \widehat{\mathcal {F}}^{\textrm{match}}[\delta \Phi ^\textrm{u}], \end{aligned}$$

with

6.13 $$\begin{aligned} \begin{aligned} \widehat{\delta \Phi ^\textrm{u}_0}&= \delta \Phi ^\textrm{u}_0 - \left( \begin{array}{c} {\mathcal {G}}^\textrm{match}_1[\delta \psi ^\textrm{u}_0](z) \\ 0\end{array}\right) ,\\ \widehat{\mathcal {F}}^{\textrm{match}}[\delta \Phi ]&=- \left( \begin{array}{c} {\mathcal {G}}^\textrm{match}_1 \big [\mathcal {F}^{\textrm{match}}_2[\delta \Phi ] \big ](z) \\ 0\end{array}\right) + \mathcal {F}^{\textrm{match}}[\delta \Phi ]. \end{aligned} \end{aligned}$$

### The matching error

For fixed $\ell \in {\mathbb {R}}$, we introduce the norm

$$ \Vert f\Vert _\ell = \sup _{z\in \overline{D_{\theta _1,\theta _2,\nu }^{\textrm{match}}}} \left| z^\ell f(z)\right| $$

and the Banach spaces

$$\begin{aligned} \begin{aligned} \mathcal {Y}_{\ell }&=\{f: \overline{\mathcal {D} _{\theta _1,\theta _2,\nu }^{ \textrm{match}}} \rightarrow \mathbb {C}; \, f\, \text {is continuous and analytic on }\mathcal {D} _{\theta _1,\theta _2,\nu }^{ \textrm{match}}\; \text {with }\Vert f\Vert _{\ell }<\infty \}, \\ \mathcal {D}\mathcal {Y}_{\ell }&=\{f: \overline{\mathcal {D}_{\theta _1,\theta _2,\nu }^{ \textrm{match}}} \rightarrow \mathbb {C}; \, f\in \mathcal {Y_\ell } \text { with }\Vert f\Vert _{\ell } + \Vert f'\Vert _{\ell +1} <\infty \}. \end{aligned} \end{aligned}$$

These Banach spaces satisfy the following properties.

#### Lemma 6.4

Let $$\ell _1,\ell _2 \in \mathbb {R}$$. Then

1. If $f\in \mathcal {Y}_{\ell _1}$, then $f\in \mathcal {Y}_{\ell _2}$, for all $\ell _2\in \mathbb {R}$. Moreover for $\ell _1 >\ell _2$
  $$ \Vert f\Vert _{\ell _2} \lesssim \kappa ^{\ell _2-\ell _1} \Vert f\Vert _{\ell _1} $$
  and for $$\ell _1<\ell _2$$,
  $$ \Vert f\Vert _{\ell _2}\lesssim \varepsilon ^{(\ell _1-\ell _2)(1-\nu )}. $$
2. If $f\in \mathcal {Y}_{\ell _1}$ and $g\in \mathcal {Y}_{\ell _2}$, then $\Vert fg\Vert _{\ell _1 + \ell _2} \le \Vert f\Vert _{\ell _1}\Vert g\Vert _{\ell _2}$.

We define the product Banach space $\mathcal {Y}_\times =\mathcal {D}\mathcal {Y}_2 \times \mathcal {Y}_4$ endowed with the product norm

6.14 $$\begin{aligned} \Vert (\phi ,\psi ) \Vert _\times = \textrm{max}\{\Vert \phi \Vert _2 + \Vert \partial _z \phi \Vert _3,\Vert \psi \Vert _4 \}. \end{aligned}$$

We note that, as claimed in Remark 6.2, $\delta \phi ^\textrm{u}\in \mathcal {D}\mathcal {Y}_3$, $\delta \psi ^\textrm{u}\in \mathcal {Y}_5$ with $\Vert \delta \phi ^\textrm{u}\Vert _3 + \Vert \partial _z \delta \phi ^\textrm{u}\Vert _4, \Vert \delta \psi ^\textrm{u}\Vert _5 \lesssim 1$ and therefore, by Lemma 6.4,

6.15 $$\begin{aligned} \Vert \delta \Phi ^\textrm{u}\Vert _\times = \textrm{max}\{|\delta \phi ^\textrm{u}\Vert _2 + \Vert \partial _z \delta \phi ^\textrm{u}\Vert _3, \Vert \delta \psi ^\textrm{u}\Vert _4 \}\lesssim \frac{1}{\kappa }. \end{aligned}$$

We now start estimating all the elements in the fixed point equation (6.11). The following lemma, whose proof is given in Sect. 6.3, deals with the operators ${\mathcal {G}}^\textrm{match}$ and $\mathcal {F}^{\textrm{match}}$ defined in (6.8) and (6.10) respectively.

#### Lemma 6.5

If $\kappa$ is big enough, the following statements are satisfied:

1. If $h\in \mathcal {Y}_\ell$ with $\ell >4$, then ${\mathcal {G}}^\textrm{match}_1 [h] \in \mathcal {Y}_{\ell -2}$ and
  $$ \Vert {\mathcal {G}}^\textrm{match}_1[h]\Vert _{\ell -2} \lesssim \Vert h \Vert _{\ell }, \qquad \Vert \partial _z {\mathcal {G}}^\textrm{match}_1[h]\Vert _{\ell -1} \lesssim \Vert h \Vert _{\ell }. $$
2. If $h \in \mathcal {Y}_{\ell }$ with $\ell >0$, then ${\mathcal {G}}^\textrm{match}_2[h] \in \mathcal {Y}_{\ell }$ and $\Vert {\mathcal {G}}^\textrm{match}_2[h]\Vert _{\ell } \lesssim \Vert h\Vert _{\ell }$.
3. If $h\in \mathcal {Y}_4$, then ${\mathcal {G}}^\textrm{match}_1 [h] \in \mathcal {Y}_{2}$ and $\Vert {\mathcal {G}}^\textrm{match}_1[h]\Vert _{2} \lesssim |\log \varepsilon | \Vert h \Vert _{2}$.
4. If $h\in \mathcal {Y}_\times =\mathcal {D}\mathcal {Y}_2 \times \mathcal {Y}_4$, then $\mathcal {F}^{\textrm{match}}[h] =\big (\mathcal {F}^{\textrm{match}}_1[h],\mathcal {F}^{\textrm{match}}_2 [h]\big )\in \mathcal {D}\mathcal {Y}_4 \times \mathcal {Y}_6$ with
  $$ \Vert \mathcal {F}^{\textrm{match}}_1 [h]\Vert _4+ \Vert \partial _z \big (\mathcal {F}^{\textrm{match}}_1[h]\big )\Vert _5+\Vert \mathcal {F}^{\textrm{match}}_2 [h]\Vert _6 \lesssim \Vert h\Vert _\times . $$
  As a consequence, by definition (6.14) of $\Vert \cdot \Vert _\times$, we have $\Vert \mathcal {G}^{\textrm{match}}[h]\Vert _\times \lesssim \frac{1}{\kappa ^2} \Vert h\Vert _\times .$

We claim now that the operator $\widehat{\mathcal {F}}^{\textrm{match}}: \mathcal {Y}_\times \rightarrow \mathcal {Y}_\times$ defined in (6.13) satisfies that, for $\kappa$ big enough,

6.16 $$\begin{aligned} \Vert \widehat{\mathcal {F}}^{\textrm{match}}[h] \Vert _\times \lesssim \frac{1}{\kappa ^2}\Vert h\Vert _\times . \end{aligned}$$

Indeed, by item (4) in Lemma 6.5, if $h\in \mathcal {Y}_\times$, then $\mathcal {F}^{\textrm{match}}_2[h] \in \mathcal {Y}_6$. Therefore, by item (1) in Lemma 6.5, ${\mathcal {G}}^\textrm{match}_1 [\mathcal {F}^{\textrm{match}}_2[h]] \in \mathcal {D}\mathcal {Y}_4$ and the estimates in item (1) apply. By Lemma 6.4, we have

$$ \Vert {\mathcal {G}}^\textrm{match}_1 [\mathcal {F}^{\textrm{match}}_2[h]] \Vert _2+ \Vert \partial _z {\mathcal {G}}^\textrm{match}_1 [\mathcal {F}^{\textrm{match}}_2[h]] \Vert _3 \lesssim \frac{1}{\kappa ^2}\Vert h\Vert _\times . $$

Then, the claim follows from item (4) of Lemma 6.5 and definition (6.10) of $\widehat{\mathcal {F}}^{\textrm{match}}$.

It follows from (6.12) that

$$ \big (\textrm{Id} - \widehat{\mathcal {F}}^{\textrm{match}}\big )\delta \Phi ^\textrm{u}= \widehat{\delta \Phi ^\textrm{u}_0}. $$

Therefore, using that $\delta \Phi ^* \in {\mathcal {Y}}_\times$ (see (6.15)) and that, by (6.16), $\textrm{Id} - \widehat{\mathcal {F}}^{\textrm{match}}:{\mathcal {Y}}_\times \rightarrow {\mathcal {Y}}_\times$ is invertible, we obtain that

$$ \delta \Phi ^\textrm{u}= \big (\textrm{Id}- \widehat{\mathcal {F}}^{\textrm{match}}\big )^{-1} [\widehat{\delta \Phi ^\textrm{u}_0}]\quad \text {and}\quad \Vert \delta \Phi ^\textrm{u}\Vert _\times \lesssim \Vert \widehat{\delta \Phi ^\textrm{u}_0}\Vert _\times . $$

Theorem 6.1 is then a consequence of the following lemma whose proof is given in Sect. 6.4.

#### Lemma 6.6

Let $\nu \in (0,1)$. If $\kappa$ is big enough, then $\Vert \widehat{\delta \Phi ^\textrm{u}_0} \Vert _\times \lesssim |\log \varepsilon |\varepsilon ^{1-\nu }$.

It remains to prove Lemmas 6.5 and 6.6.

### Proof of Lemma 6.5

The proof of the three first items of Lemma 6.5 can be found in the proof of Lemma 6.2 in [31] (see also [5]).

Now we prove item (4). We first note that, from definition (2.27) of $\mathcal {J}^{\textrm{in}}_1, \mathcal {J}^{\textrm{in}}_2$,

$$\begin{aligned} D_{\Phi } \mathcal {J}^{\textrm{in}}[\Phi ] (z)= \left( \begin{array}{cc} -\frac{12}{z} \phi - 6 \phi ^2 &  -1 \\ \mathfrak {g}[\Phi ]&  - 6 \left( \frac{1}{z} + \phi \right) ^2 \end{array}\right) , \\ D_{\partial _z \phi } \mathcal {J}^{\textrm{in}}[\Phi ] (z) = \left( 0, -24 \left( \frac{1}{z} + \phi \right) \left( -\frac{1}{z^2} + \partial _z \phi \right) \right) ^{\top }, \end{aligned}$$

where

$$ \mathfrak {g}[\Phi ](z) = -12 \left( \frac{1}{z} + \phi \right) \left( \psi + 2 \left( \frac{1}{z} + \phi \right) ^3 \right) - 36 \left( \frac{1}{z} + \phi \right) ^4 - 12 \left( -\frac{1}{z^2} + \partial _z \phi \right) ^2. $$

Let us denote

$$\begin{aligned} P(z)=D_\Phi \mathcal {J}^{\textrm{in}}[\Phi ](z) - \left( \begin{array}{cc} 0 & -1 \\ 0 &  0 \end{array}\right) , \qquad Q(z)= D_{\partial _z \phi } \mathcal {J}^{\textrm{in}}[\Phi ] (z). \end{aligned}$$

Then, $P=(P_{ij})_{i,j}$ is a $2\times 2$ matrix and, for $\Phi \in \mathcal {Y}_3\times \mathcal {Y}_3$, its coefficients satisfy

$$\begin{aligned} |P_{11}(z)|\lesssim \frac{1}{|z|^4},\quad P_{12}(z)=0,\quad |P_{21}(z)|\lesssim \frac{1}{|z|^4},\quad |P_{22}(z)|\lesssim \frac{1}{|z|^2},\quad \end{aligned}$$

whereas *Q* is a 2-dimensional vector which, for $\Phi \in \mathcal {Y}_3\times \mathcal {Y}_3$, satisfies

$$\begin{aligned} Q_1(z)=0,\qquad |Q_{2}(z)|\lesssim \frac{1}{|z|^3}. \end{aligned}$$

Finally, by definition (6.5) of $\mathcal {B}_1^\textrm{u}(z), \mathcal {B}_2^\textrm{u}(z)$, if $h\in \mathcal {D}\mathcal {Y}_2 \times \mathcal {Y}_4$, then we have

$$ \Vert \mathcal {B}_1^\textrm{u}\cdot h \Vert _6 , \, \Vert \mathcal {B}_2^\textrm{u}\cdot h \Vert _6 \lesssim \Vert h\Vert _\times , $$

and by item (1) and item  (2) of Lemma 6.5, $\mathcal {F}^{\textrm{match}}[h] \in \mathcal {D}\mathcal {Y}_4 \times \mathcal {Y}_6$ with bounded norm. This completes the proof of Lemma 6.5.

### Proof of Lemma 6.6

We introduce

$$ \widetilde{\delta \phi _0^\textrm{u}} = z^3 a_{\phi ^\textrm{u}} + \frac{1}{z^2 } b_{\phi ^\textrm{u}}, \qquad \widetilde{\delta \psi _0^\textrm{u}} = e^{i(z-z_1) }a_{\psi ^\textrm{u}} + e^{-i(z-z_2) }b_{\psi ^\textrm{u}}, $$

where $a_{\phi ^\textrm{u}}, b_{\phi ^\textrm{u}}, a_{\psi ^\textrm{u}}$ and $b_{\psi ^\textrm{u}}$ are defined by (6.7) with $\phi = \phi ^\textrm{u}$ and $\psi =\psi ^\textrm{u}$. From (6.13), we have that $\widehat{\delta \Phi ^\textrm{u}_0} = \big ( \widehat{\delta \phi ^\textrm{u}_0}, \widehat{\delta \psi ^\textrm{u}_0}\big )$ is defined by

$$\begin{aligned} \widehat{\delta \phi _0^\textrm{u}}(z)&= \delta \phi _0^\textrm{u}(z) - {\mathcal {G}}^\textrm{match}_1[\delta \psi _0^\textrm{u}] = \widetilde{\delta \phi _0^\textrm{u}}(z) + {\mathcal {G}}^\textrm{match}_1[A_1^\textrm{u}](z) - {\mathcal {G}}^\textrm{match}_1[\delta \psi _0^\textrm{u}],\\ \widehat{\delta \psi _0^\textrm{u}}(z)&= \delta \psi _0^\textrm{u}(z) = \widetilde{\delta \psi _0^\textrm{u}}(z) + {\mathcal {G}}^\textrm{match}_2[A_2^\textrm{u}](z), \end{aligned}$$

where $A^\textrm{u}=\big (A^\textrm{u}_1,A^\textrm{u}_2\big )$ is defined by

6.17 $$\begin{aligned} A^\textrm{u}(z) = \mathcal {A}[\Phi ^\textrm{u}](z) = \mathcal {J}^{\textrm{match}}_1[\Phi ^\textrm{u}](z) - \mathcal {J}^{\textrm{in}}_1 [\Phi ^\textrm{u}](z). \end{aligned}$$

We recall that $\phi ^\textrm{u}\in D\mathcal {Y}_3$ and $\psi ^\textrm{u}\in \mathcal {Y}_5$, see (6.4). The following lemma estimates $\widetilde{\delta \phi _0^\textrm{u}}(z)$.

#### Lemma 6.7

Fix $\nu \in (0,1)$. If $\varepsilon >0$ is small enough, then we have for all $z\in D_{\theta _1,\theta _2,\nu }^\textrm{match}$,

$$ \big |z^2 \widetilde{\delta \phi _0^\textrm{u}}(z) \big | + \big |z^3\partial _z \widetilde{\delta \phi _0^\textrm{u}}(z) \big |+ \big |z^4 \widetilde{\delta \psi _0^\textrm{u}}(z) \big | \lesssim \varepsilon ^{1-\nu }. $$

#### Proof

By definition (6.7), we have

$$ |a_\phi ^\textrm{u}|\lesssim \frac{1}{|z_1|^6} \lesssim \varepsilon ^{6(1-\nu )}, \qquad |b_\phi ^\textrm{u}| \lesssim \frac{1}{|z_1|} \lesssim \varepsilon ^{1-\nu }, \qquad |a_\psi ^\textrm{u}|, |b_\psi ^\textrm{u}| \lesssim \varepsilon ^{5(1-\nu )}. $$

Then, for $z\in D_{\theta _1,\theta _2}^{\nu , \textrm{match}}$, using that $|z| \lesssim \min \{ |z_1|, |z_2|\} \lesssim \varepsilon ^{-(1-\nu )}$, we obtain

$$\begin{aligned} \begin{aligned} \big |z^2 \delta \phi _0^\textrm{u}(z) \big |&= \left| z^5 a_{\phi } + b_{\phi } \right| \lesssim |z|^5 \varepsilon ^{6(1-\nu )} + \varepsilon ^{1-\nu } \lesssim \varepsilon ^{1-\nu }, \\ \big |z^4 \delta \psi _0^\textrm{u}(z) \big |&\lesssim \varepsilon ^{5(1-\nu )} |z|^4 \big (e^{-\Im (z-z_1)} + e^{\Im (z-z_2)} \big ) \lesssim \varepsilon ^{1-\nu }, \end{aligned} \end{aligned}$$

where in the last inequality we have used that $\Im z_2>\Im z >\Im z_1$. $\square$

Next we analyze ${\mathcal {G}}^\textrm{match}[A^\textrm{u}]$. To do so, we look for an explicit expression of $\mathcal {J}^{\textrm{match}}$.

#### Lemma 6.8

The fixed point equation (2.3) in the inner variables (6.1) can be written as

$$\begin{aligned} \left\{ \begin{array}{l} \mathcal {L}_1^{\textrm{in}} \phi = \mathcal {J}^{\textrm{match}}_1[\phi ,\psi ;\varepsilon ], \\ \mathcal {L}_2^{\textrm{in}} \psi = \mathcal {J}^{\textrm{match}}_2[\phi ,\psi ;\varepsilon ], \end{array} \right. \end{aligned}$$

with

$$\begin{aligned} \left\{ \begin{array}{l} \mathcal {J}^{\textrm{match}}_1 [\phi ,\psi ;\varepsilon ](z)= \mathcal {J}^{\textrm{in}}_1[\phi ,\psi ](z) + \mathcal {A}_1[\phi ,\psi ;\varepsilon ](z), \vspace{0.2cm}\\ \mathcal {J}^{\textrm{match}}_2[\phi ,\psi ;\varepsilon ](z)= \mathcal {J}^{\textrm{in}}_1[\phi ,\psi ](z) + \mathcal {A}_2[\phi ,\psi ;\varepsilon ](z), \end{array} \right. \end{aligned}$$

where, for $$z \in \mathcal {D}_{\theta _1,\theta _2,\nu }^{\textrm{match}}$$,

6.18 $$\begin{aligned} \big | \mathcal {A}_1[\phi ^\textrm{u},\psi ^\textrm{u};\varepsilon ](z) \big | \lesssim \frac{\varepsilon }{|z|^4}, \qquad \big | \mathcal {A}_2[\phi ^\textrm{u},\psi ^\textrm{u};\varepsilon ](z) \big | \lesssim \frac{\varepsilon }{|z|^4}. \end{aligned}$$

#### Proof

An straightforward computation shows that in the inner variables, the fixed point equation (2.3) can be expressed as

$$\begin{aligned} \left\{ \begin{array}{l} \mathcal {L}_1^{\textrm{in}} \phi = \mathcal {J}^{\textrm{match}}_1[\phi ,\psi ;\varepsilon ], \vspace{0.2cm}\\ \mathcal {L}_2^{\textrm{in}} \psi = \mathcal {J}^{\textrm{match}}_2[\phi ,\psi ;\varepsilon ], \end{array} \right. \end{aligned}$$

with

$$\begin{aligned} \left\{ \begin{array}{l} \mathcal {J}^{\textrm{match}}_1[\phi ,\psi ;\varepsilon ](z) = \varepsilon ^2 \phi (z) \left[ -1 + 2u_0(x_-+\varepsilon z )\right] + \phi (z) \left[ 6\gamma \varepsilon ^2 u_0^2(x_-+\varepsilon z ) + \frac{6}{z^2}\right] \vspace{0.2cm} \\ \hspace{5cm} + \varepsilon ^3 c_{- 1}^{-1}\mathcal {F}_1[\varepsilon ^{-1} c_{- 1} \phi , \varepsilon ^{-3} c_{- 1} \psi ](x_-+\varepsilon z), \vspace{0.2cm}\\ \mathcal {J}^{\textrm{match}}_2[\phi ,\psi ;\varepsilon ](z) = \varepsilon ^{5} c_{- 1}^{-1} \mathcal {F}_2[\varepsilon ^{-1} c_{- 1} \phi , \varepsilon ^{-3} c_{- 1} \psi ] (x_-+\varepsilon z). \end{array} \right. \end{aligned}$$

Using the expression (2.5) of $\mathcal {F}=({\mathcal {F}}_1,{\mathcal {F}}_2)$ we obtain

$$\begin{aligned} \mathcal {J}^{\textrm{match}}_1[\phi ,\psi ;\varepsilon ](z)=&-\psi -\frac{6}{z} \phi ^2 -2 \phi ^3 + \mathcal {A}_1[\phi ;\varepsilon ](z)\\ =&\mathcal {J}^{\textrm{in}}_1[\phi ,\psi ](z) + \mathcal {A}_1[\phi ,\psi ;\varepsilon ](z), \end{aligned}$$

with

$$\begin{aligned} \mathcal {A}_1[\phi ,\psi ;\varepsilon ](z)=&\varepsilon ^2 \phi (z) \left[ -1 + 2\hat{u}_0(z)\right] + \phi (z) \left[ 6\gamma \varepsilon ^2 \hat{u}_0^2(z) + \frac{6}{z^2}\right] \\&+ \left( {c_{- 1} }\varepsilon + 6 \varepsilon \gamma c_{- 1} \hat{u}_0(z) + \frac{6}{ z} \right) \phi ^2. \end{aligned}$$

Analogously, tedious but easy computations lead to

$$\begin{aligned} \mathcal {J}^{\textrm{match}}_2[\phi ,\psi ;\varepsilon ](z)=&-6 \left( \frac{1}{z} +\phi \right) ^2 \left( \psi + 2 \left( \frac{1}{z} + \phi \right) ^3 \right) -12 \left( \frac{1}{z} + \phi \right) \left( -\frac{1}{z^2} + \partial _z \phi \right) ^2 \\  &- 6 \left( \frac{1}{z} + \phi \right) ^2 C[\phi ,\psi ;\varepsilon ](z) + \left( \psi + 2 \left( \frac{1}{z} + \phi \right) ^3 \right) B[\phi ;\varepsilon ](z) \\  &+ B[\phi ;\varepsilon ](z) \cdot C[\phi ,\psi ;\varepsilon ](z) + D[\phi ,\psi ;\varepsilon ](z)\\ =&\mathcal {J}^{\textrm{in}}_2[\phi ,\psi ](z) + \mathcal {A}_2[\phi ,\psi ;\varepsilon ](z) \end{aligned}$$

with

$$\begin{aligned} B[\phi ;\varepsilon ](z)=&-6\left( \varepsilon c_{- 1}^{-1} \hat{u}_0 - \frac{1}{z} \right) \left( \frac{1}{z} + 2 \phi + \varepsilon c_{- 1}^{-1} \hat{u}_0 \right) + 2 \varepsilon c_{- 1} (\varepsilon c_{- 1}^{-1} \hat{u}_0 + \phi ),\\ C[\phi ,\psi ;\varepsilon ](z) =&\varepsilon ^2 ( \varepsilon c_{- 1}^{-1} \hat{u}_0 + \phi ) - \varepsilon c_{- 1} (\varepsilon c_{- 1}^{-1} \hat{u}_0 + \phi )^2 \\&+2 \left( \varepsilon c_{- 1}^{-1} \hat{u}_0 - \frac{1}{z}\right) \left[ \left( \varepsilon c_{- 1}^{-1}\hat{u}_0 + \phi \right) ^2 + \left( \varepsilon c_{- 1}^{-1} \hat{u}_0 + \phi \right) \left( \frac{1}{z} + \phi \right) \right] \\  &+ 2 \left( \varepsilon c_{- 1}^{-1} \hat{u}_0 - \frac{1}{z}\right) \left( \frac{1}{z} + \phi \right) ^2 ,\\ D[\phi ,\psi ;\varepsilon ](z) =&2 c_{- 1} \varepsilon (\varepsilon c_{- 1}^{-1} \hat{u}_0' + \partial _z \phi )^2 - 12 \left( \varepsilon c_{- 1}^{-1} \hat{u}_0 -\frac{1}{z}\right) \left( \varepsilon c_{- 1}^{-1} \hat{u}_0' +\partial _z \phi \right) ^2 \\&-12 \left( \frac{1}{z} +\phi \right) \left( \varepsilon c_{- 1}^{-1} \hat{u}_0'+ \frac{1}{z^2} \right) \left( 2\partial _z \phi + \varepsilon c_{- 1}^{-1}\hat{u}_0' - \frac{1}{z^2}\right) . \end{aligned}$$

To prove the bounds for $\mathcal {A}_1[\phi ^\textrm{u},\psi ^\textrm{u};\varepsilon ], \mathcal {A}_2[\phi ^\textrm{u}, \psi ^\textrm{u}; \varepsilon ]$, we recall that $c_{-1}^{-1}= \sqrt{|\gamma |}$ with $\gamma <0$ and take into account (6.2) and (6.4), to obtain

$$ \left| \frac{1}{z} + \phi ^\textrm{u}(z) \right| \lesssim \frac{1}{|z|}, \qquad \left| \varepsilon c_{-1}^{-1} \hat{u_0}- \frac{1}{z}\right| \lesssim \varepsilon , \qquad \left| \varepsilon c_{- 1}^{-1}\hat{u}_0' + \frac{1}{z^2} \right| \lesssim \varepsilon ^2. $$

The proof of (6.18) follows from these bounds and the explicit expressions of the functions involved. $\square$

Lemma 6.8, together with items (1) and (2) of Lemma 6.5, implies that, for all $z\in \mathcal {D}_{\theta _1,\theta _2,\nu }^\textrm{match}$, we have

$$ \big |z^2 {\mathcal {G}}^\textrm{match}_1[A_1^\textrm{u}] (z) \big |+ \big |z^3 \partial _z {\mathcal {G}}^\textrm{match}_1[A_1^\textrm{u}] (z) \big | + |z^4 {\mathcal {G}}^\textrm{match}_2[A_2^\textrm{u}](z) \big | \lesssim \varepsilon |\log \varepsilon |, $$

where we recall that $A^\textrm{u}(z)=\mathcal {A}[\phi ^\textrm{u},\psi ^\textrm{u}]$ (see (6.17)). This estimate and Lemma 6.7 imply that for all $z\in \mathcal {D}_{\theta _1,\theta _2,\nu }^\textrm{match}$, we have

$$\begin{aligned} \big |z^2 \delta \phi _0^\textrm{u}(z) \big | + \big |z^3\partial _z \delta \phi _0^\textrm{u}(z) \big |+ \big |z^4\delta \psi _0^\textrm{u}(z) \big | \lesssim \varepsilon ^{1-\nu }. \end{aligned}$$

To estimate $\widehat{\delta \phi _0^\textrm{u}}(z)$, it only remains to analyze ${\mathcal {G}}^\textrm{match}_1 [ \delta \psi _0^\textrm{u}]$. To this end, it is enough to recall that $\big |z^4\delta \psi _0^\textrm{u}(z) \big | \lesssim \varepsilon ^{1-\nu }$ and Lemma 6.5 imply

$$\begin{aligned} \big | z^2 {\mathcal {G}}^\textrm{match}_1 [ \delta \psi _0^\textrm{u}] (z) \big | \lesssim |\log \varepsilon | \varepsilon ^{1-\nu }. \end{aligned}$$

Therefore, recalling that $\widehat{\delta \psi _0^{\textrm{u}}} = \delta \psi _0^\textrm{u}$, we conclude that, for all $z\in \mathcal {D}_{\theta _1,\theta _2,\nu }^\textrm{match}$, we have

$$ \big |z^2 \widehat{\delta \phi _0^\textrm{u}} (z) \big |+ \big |z^3\partial _z \widehat{\delta \phi _0^\textrm{u}}(z)\big | + |z^4 \widehat{\psi _0^\textrm{u}}(z) \big | \lesssim \varepsilon ^{1-\nu } |\log \varepsilon |. $$

This completes the proof of Lemma 6.6.

## The Difference Between the Invariant Manifolds

Here we prove Proposition 2.7 for $\Delta \eta ^{\textrm{u}}$. The proof for $\Delta \eta ^{\textrm{s}}$ is analogous. We define first the following Banach spaces with norms with exponential weights

$$\begin{aligned} {\mathcal {E}}_\ell =\{h:\overline{E^{\textrm{out},\textrm{u}}_\kappa }\rightarrow {\mathbb {C}}; \, h\,\, \text {continuous and real-analytic on }E^{\textrm{out},\textrm{u}}_\kappa \; \text {with } \Vert h\Vert _{\ell ,\textrm{exp}}<\infty \}, \end{aligned}$$

where

7.1 $$\begin{aligned} \Vert h\Vert _{\ell ,\textrm{exp}}=\sup _{x\in \overline{E^{\textrm{out},\textrm{u}}_\kappa }}\left| (x-x_-)^\ell (x-x_+)^\ell (x-\bar{x}_-)^\ell (x-{\bar{x}}_+)^\ell e^{\frac{1}{\varepsilon }\left( \pi -|\Im x|\right) }h(x)\right| . \end{aligned}$$

We also consider the Banach space

$$\begin{aligned} {\mathcal {E}}_\times =\{h=(h_1,h_2):\overline{E^{\textrm{out},\textrm{u}}_\kappa }\rightarrow {\mathbb {C}}^2; \; h_1, \, h_2\in {\mathcal {E}}_{0,\textrm{exp}}\,\, \text { with }\ \Vert h\Vert _{\times }<\infty \}, \end{aligned}$$

where

7.2 $$\begin{aligned} \Vert h\Vert _{\times }= \textrm{max}\big \{\varepsilon ^{-1}\Vert h_1\Vert _{0,\textrm{exp}},\Vert h_2\Vert _{0,\textrm{exp}}+\varepsilon \Vert \partial _x h_2\Vert _{0,\textrm{exp}} \big \}. \end{aligned}$$

We look for an integral equation in these Banach spaces which has as a unique solution $(\Delta \zeta ^{\textrm{u}},\Delta \eta ^{\textrm{u}})$. The following lemma presents suitable inverses of the operators $\widehat{\mathcal {L}}_1$ and ${\mathcal {L}}_2$ defined by (2.15) and (2.4) respectively. Its proof follows the same lines as the proof of Lemma 7.1 in [31].

### Lemma 7.1

The operators

$$\begin{aligned} \widehat{{\mathcal {G}}}_1[h](x)= u_0''(x)\int _0^x\frac{1}{u_0''(s)}h(s)ds \end{aligned}$$

and

$$\begin{aligned} \begin{aligned} \widehat{{\mathcal {G}}}_2[h] (x)=&-\frac{i\varepsilon }{2} e^{i\varepsilon ^{-1}x}\int _{\rho _{-}}^x e^{-i\varepsilon ^{-1}s} h(s)ds+\frac{i\varepsilon }{2} e^{-i\varepsilon ^{-1}x}\int _{\overline{\rho _{-}}}^x e^{i\varepsilon ^{-1}s} h(s)ds\\&+\frac{i\varepsilon \sin \left( \frac{\rho _--x}{\varepsilon }\right) }{2\sin \left( \frac{\rho _--\overline{\rho _-}}{\varepsilon }\right) }e^{i\varepsilon ^{-1}\overline{\rho _-}}\int _{\rho _-}^{\overline{\rho _-}} e^{-i\varepsilon ^{-1}s} h(s)ds\\&-\frac{i\varepsilon \sin \left( \frac{\overline{\rho _-}-x}{\varepsilon }\right) }{2\sin \left( \frac{\overline{\rho _-}-\rho _-}{\varepsilon }\right) }e^{i\varepsilon ^{-1}\rho _-}\int ^{\rho _-}_{\overline{\rho _-}} e^{i\varepsilon ^{-1}s} h(s)ds, \end{aligned} \end{aligned}$$

with $\rho _{-}=x_--i\kappa \varepsilon$, have the following properties.

- Fix $\ell \in {\mathbb {R}}$. The operator $\widehat{{\mathcal {G}}}_1$ is well defined from ${\mathcal {E}}_\ell$ to ${\mathcal {E}}_\ell$ and satisfies
  $$\begin{aligned} \left\| \widehat{{\mathcal {G}}}_1[h]\right\| _{\ell ,\textrm{exp}}\le M\varepsilon \Vert h\Vert _{\ell ,\textrm{exp}}. \end{aligned}$$
  It is also well-defined from ${\mathcal {E}}_\ell$ to ${\mathcal {E}}_0$ and satisfies
  $$\begin{aligned} \left\| \widehat{{\mathcal {G}}}_1[h]\right\| _{0,\textrm{exp}}\le \frac{M\varepsilon }{(\kappa \varepsilon )^{\ell }}\Vert h\Vert _{\ell ,\textrm{exp}}. \end{aligned}$$
  Furthermore, $\widehat{\mathcal {L}}_1\circ \widehat{{\mathcal {G}}}_1=\textrm{Id}$ and, for $h\in {\mathcal {E}}_\ell$,
  $$\begin{aligned} \widehat{{\mathcal {G}}}_1(h)(0)=0. \end{aligned}$$
- Fix $\ell >1$. The operator $\widehat{{\mathcal {G}}}_2$ is well defined from ${\mathcal {E}}_\ell$ to ${\mathcal {E}}_0$ and satisfies
  $$\begin{aligned} \begin{aligned} \left\| \widehat{{\mathcal {G}}}_2[h]\right\| _{0,\textrm{exp}}&\le \frac{M\varepsilon }{(\kappa \varepsilon )^{\ell -1}}\Vert h\Vert _{\ell ,\textrm{exp}},\\ \left\| \partial _x\widehat{{\mathcal {G}}}_2[h]\right\| _{0,\textrm{exp}}&\le \frac{M}{(\kappa \varepsilon )^{\ell -1}}\Vert h\Vert _{\ell ,\textrm{exp}}. \end{aligned} \end{aligned}$$
  Furthermore, ${\mathcal {L}}_2\circ \widehat{{\mathcal {G}}}_2=\textrm{Id}$ and, for $h\in {\mathcal {E}}_\ell$
  $$\begin{aligned} \widehat{{\mathcal {G}}}_2[h](\rho _-)=0\quad \text {and}\quad \widehat{{\mathcal {G}}}_2[h](\overline{\rho _-})=0. \end{aligned}$$

The functions $(\Delta \zeta ^\textrm{u},\Delta \eta ^\textrm{u})$ introduced in Lemma 2.6 satisfy equation (2.14). Now, by the properties of the operators $\widehat{\mathcal {G}}_1$ and $\widehat{\mathcal {G}}_2$ introduced in Lemma 7.1, the functions $(\Delta \zeta ^\textrm{u},\Delta \eta ^\textrm{u})$ must be a fixed point of the operator

7.3 $$\begin{aligned} {\mathcal {P}}\big [\Delta \zeta ,\Delta \eta \big ](x)=\begin{pmatrix} \widehat{\mathcal {G}}_1\circ \widehat{\mathcal {N}}_1 \big [\Delta \zeta ,\Delta \eta , \Delta \eta ' \big ](x)\\ C_1^\textrm{u}e^{\frac{ix}{\varepsilon }}+C_2^\textrm{u}e^{-\frac{ix}{\varepsilon }}+\widehat{\mathcal {G}}_2\circ \widehat{\mathcal {N}}_2 \big [\Delta \zeta ,\Delta \eta ,\Delta \eta ' \big ](x) \end{pmatrix} \end{aligned}$$

for some constants $C_1^\textrm{u}$, $C_2^\textrm{u}$ satisfying (2.20).

Note that by Lemma 7.1, the function ${\mathcal {R}}^\textrm{u}$ introduced in Lemma 2.7 is given by

7.4 $$\begin{aligned} {\mathcal {R}}^\textrm{u}=\widehat{\mathcal {G}}_2\circ \widehat{\mathcal {N}}_2 \big [\Delta \zeta ^\textrm{u},\Delta \eta ^\textrm{u},\partial _x\Delta \eta ^\textrm{u}\big ]. \end{aligned}$$

and it satisfies the properties in (2.21). Therefore, it only remains to obtain the estimates in (2.22).

To this end, we use a fixed point argument relying on (7.3). However, the operator ${\mathcal {P}}$ is not contractive and, therefore, proceeding as in Sect. 3, we consider the operator

$$\begin{aligned} \widehat{\mathcal {P}}\big [\Delta \zeta ,\Delta \eta \big ]=\begin{pmatrix} {\mathcal {P}}_1 \big [\Delta \zeta ,{\mathcal {P}}_2\big [\Delta \zeta ,\Delta \eta \big ] \big ]\\ {\mathcal {P}}_2 \big [\Delta \zeta ,\Delta \eta \big ], \end{pmatrix} \end{aligned}$$

which has the same fixed points as ${\mathcal {P}}$ and is contractive. Note that both operators ${\mathcal {P}}$ and $\widehat{\mathcal {P}}$ are affine. The following lemma gives the Lipschitz constant of the operator ${\mathcal {P}}$. Its proof is a direct consequence of Lemmas 2.6 and 7.1.

### Lemma 7.2

There exists $M>0$ such that, for any $(\Delta \zeta _1,\Delta \eta _1), (\Delta \zeta _2,\Delta \eta _2) \in {\mathcal {E}}_\times$, the operator ${\mathcal {P}}$ satisfies

$$\begin{aligned} \begin{aligned} \left\| {\mathcal {P}}_1 \big [\Delta \zeta _1,\Delta \eta _1 \big ]-{\mathcal {P}}_1 \big [\Delta \zeta _2,\Delta \eta _2 \big ]\right\| _{0,\textrm{exp}}\le&M\varepsilon \left\| \Delta \eta _1-\Delta \eta _2\right\| _{0,\textrm{exp}} \\  &+\frac{M\varepsilon }{\kappa }\left\| (\Delta \zeta _1,\Delta \eta _1)-(\Delta \zeta _2,\Delta \eta _2)\right\| _\times , \\ \left\| {\mathcal {P}}_2 \big [\Delta \zeta _1,\Delta \eta _1 \big ]-{\mathcal {P}}_2 \big [\Delta \zeta _2,\Delta \eta _2 \big ]\right\| _{0,\textrm{exp}}\le&\frac{M}{\kappa }\left\| (\Delta \zeta _1,\Delta \eta _1)-(\Delta \zeta _2,\Delta \eta _2)\right\| _\times ,\\ \left\| \partial _x{\mathcal {P}}_2 \big [\Delta \zeta _1,\Delta \eta _1 \big ]-\partial _x{\mathcal {P}}_2 \big [\Delta \zeta _2,\Delta \eta _2 \big ]\right\| _{0,\textrm{exp}}\le&\frac{M}{\varepsilon \kappa }\left\| (\Delta \zeta _1,\Delta \eta _1)-(\Delta \zeta _2,\Delta \eta _2)\right\| _\times . \end{aligned} \end{aligned}$$

Lemma 7.2 implies that $\widehat{\mathcal {P}}$ satisfies

$$\begin{aligned} \left\| \widehat{\mathcal {P}}_1 \big [\Delta \zeta _1,\Delta \eta _1 \big ]-\widehat{\mathcal {P}}\big [\Delta \zeta _2,\Delta \eta _2 \big ]\right\| _\times \le \frac{M}{\kappa }\left\| (\Delta \zeta _1,\Delta \eta _1)-(\Delta \zeta _2,\Delta \eta _2)\right\| _\times . \end{aligned}$$

Therefore, taking $\kappa >0$ large enough, $\widehat{\mathcal {P}}$ is contractive and has the unique fixed point $(\Delta \zeta ^\textrm{u},\Delta \eta ^\textrm{u})$.

We use $\widehat{\mathcal {P}}$ to obtain estimates of the fixed point with respect to the norm introduced in (7.2). Indeed, since it is a fixed point, it can be written as

$$\begin{aligned} (\Delta \zeta ^\textrm{u},\Delta \eta ^\textrm{u})=\widehat{\mathcal {P}}[0,0]+\left[ \widehat{\mathcal {P}}\big [\Delta \zeta ^\textrm{u},\Delta \eta ^\textrm{u}\big ]-\widehat{\mathcal {P}}[0,0]\right] \end{aligned}$$

and, therefore,

$$\begin{aligned} \begin{aligned} \left\| (\Delta \zeta ^\textrm{u},\Delta \eta ^\textrm{u})\right\| _\times \le&\left\| \widehat{\mathcal {P}}(0,0) \right\| _\times +\left\| \widehat{\mathcal {P}}(\Delta \zeta ^\textrm{u},\Delta \eta ^\textrm{u})-\widehat{\mathcal {P}}(0,0)\right\| _\times \\ \le&\left\| \widehat{\mathcal {P}}(0,0)\right\| _\times +\frac{M}{\kappa }\left\| (\Delta \zeta ^\textrm{u},\Delta \eta ^\textrm{u})\right\| _\times . \end{aligned} \end{aligned}$$

Taking $\kappa$ large enough implies that

$$\begin{aligned} \left\| (\Delta \zeta ^\textrm{u},\Delta \eta ^\textrm{u})\right\| _\times \le 2\left\| \widehat{\mathcal {P}}(0,0)\right\| _\times . \end{aligned}$$

Therefore, it only remains to estimate

$$\begin{aligned} \widehat{\mathcal {P}}[0,0] (x) = \begin{pmatrix}\widehat{{\mathcal {P}}}_1[0,0](x) \\ {\mathcal {P}}_2[0,0](x)\end{pmatrix} = \begin{pmatrix}{\mathcal {P}}_1 [0, {{\mathcal {P}}}_2[0,0]](x)\\ C_1^\textrm{u}e^{\frac{ix}{\varepsilon }}+C_2^\textrm{u}e^{-\frac{ix}{\varepsilon }} \end{pmatrix}, \end{aligned}$$

where $C_1^\textrm{u}$, $C_2^\textrm{u}$ are constants satisfying (2.20).

By the definition of the norm (7.1), we have

$$\begin{aligned} \left\| {\mathcal {P}}_2[0,0] \right\| _{0,\textrm{exp}}\le \left( |C_1^\textrm{u}|+|C_2^\textrm{u}|\right) e^{\frac{\pi }{\varepsilon }}, \end{aligned}$$

which by Lemma 7.2, implies

$$\begin{aligned} \left\| {\mathcal {P}}_1[0,{\mathcal {P}}_2[0,0] ]\right\| _{0,\textrm{exp}}\lesssim \left( |C_1^\textrm{u}|+|C_2^\textrm{u}|\right) e^{\frac{\pi }{\varepsilon }}. \end{aligned}$$

Therefore,

$$\begin{aligned} \left\| (\Delta \zeta ^\textrm{u},\Delta \eta ^\textrm{u})\right\| _\times \le 2\left\| \widehat{\mathcal {P}}[0,0]\right\| _\times \lesssim \left( |C_1^\textrm{u}|+|C_2^\textrm{u}|\right) e^{\frac{\pi }{\varepsilon }}. \end{aligned}$$

Finally, by definition (7.4) of ${\mathcal {R}}^\textrm{u}$

$$\begin{aligned} {\mathcal {R}}^\textrm{u}=\widehat{\mathcal {P}}_2 \big [\Delta \zeta ^\textrm{u},\Delta \eta ^\textrm{u}\big ]-\widehat{\mathcal {P}}_2 [0,0 ], \end{aligned}$$

we obtain

$$\begin{aligned} \left\| {\mathcal {R}}^\textrm{u}\right\| _{0,\textrm{exp}}\le \frac{M}{\kappa }\left\| (\Delta \zeta ^\textrm{u},\Delta \eta ^\textrm{u})\right\| _\times \lesssim \frac{1}{\kappa }\left( |C_1^\textrm{u}|+|C_2^\textrm{u}|\right) e^{\frac{\pi }{\varepsilon }}, \end{aligned}$$

which concludes the proof of Proposition 2.7.

## Data Availability

Data sharing not applicable to this article as no datasets were generated or analyzed during the current study.

## Appendix A. Proof of Lemma 2.1

We take $\beta =\sqrt{1+9\gamma } \in (0,1)$. It is straightforward to check that $u_0''(x) =0$ if and only if

$$ \beta \cosh ^2 x - \cosh x - 2 \beta =0 $$

so that

$$ \cosh x = \frac{1}{2\beta } \left( 1 \pm \sqrt{1+ 8\beta ^2}\right) \in \mathbb {R}. $$

Writing $x= a+ib$, we have that

$$ \cosh a \cos b + i \sinh a \sin b= \frac{1}{2\beta } \left( 1 \pm \sqrt{1+ 8\beta ^2}\right) . $$

Therefore, $\sinh a \sin b=0$. If $a=0$, then

$$ \cos b = g_\pm (\beta ):=\frac{1}{2\beta } \left( 1 \pm \sqrt{1+ 8\beta ^2}\right) . $$

We impose

$$ | 1 \pm \sqrt{1+ 8 \beta ^2 } | \le 2 \beta $$

and obtain the condition

$$ \pm \sqrt{1+ 8 \beta ^2} \le -1 -2 \beta ^2 $$

that it is always true, taking the negative sign and $\beta \in (0,1)$. This implies that, for $\beta \in (0,1)$,

$$ -1< \frac{1}{2\beta } \left( 1 \pm \sqrt{1+ 8\beta ^2}\right) < 0 $$

and therefore $b=\textrm{acos}(g_-\beta )) \in \left( \frac{\pi }{2}, \pi \right)$. Then $u''_0(\pm i b)=0$.

On the other hand, if $b=0$, then

$$ \cosh a = g_{\pm }(\beta ) = \frac{1}{2\beta } \left( 1 \pm \sqrt{1+ 8\beta ^2}\right) . $$

Since $g_-(\beta )<-1$, we need to study the zeros of $\cosh a = g_+(\beta )$. We notice that, since $\beta \in (0,1)$,

$$ \cosh a = g_+ (\beta )> \frac{1}{\beta }>1 $$

and that implies that $a=\textrm{acosh} ( g_+(\beta )) > \alpha$ and $u''_0(\pm a)=0$.

Finally, when $b= \pm i \pi$, then

$$ \cosh a= - g_{\pm }(\beta ) = \frac{1}{2\beta } \left( \pm \sqrt{1+ 8\beta ^2}-1 \right) $$

so that

$$ \cosh a = -g_+(\beta )= \frac{1}{2\beta } \left( \sqrt{1+ 8\beta ^2}-1 \right) < \frac{1}{\beta }. $$

## Appendix B. Proof of Proposition 2.9

Here we prove that the constant $\Theta$ is not zero. To this end, it is convenient to work with just one function instead of two, as in the inner equation (2.25). Indeed, note that it is easy to check that if one defines

$$\begin{aligned} \Phi =\frac{1}{z}+\phi , \end{aligned}$$

it satisfies the fourth order equation

B.1 $$\begin{aligned} \partial _z^4\Phi +\partial _z^2\Phi =2\Phi ^3. \end{aligned}$$

We have the following lemma.

### Lemma B.1

The functions

$$\begin{aligned} \Phi ^\star (z)=\frac{1}{z}+\phi ^{0,\star }(z), \end{aligned}$$

where $\phi ^{0,\star }$ are the functions obtained in Theorem 2.8, are asymptotic to the same series at $z=\infty$ (within their domain of definition), which is of the form

$$\begin{aligned} {\hat{\Phi }}(z)=\sum _{n\ge 0}\frac{a_n}{z^{2n+1}}, \end{aligned}$$

with coefficients satisfying that $a_n\in {\mathbb {R}}$,

B.2 $$\begin{aligned} a_n(-1)^n>0 \end{aligned}$$

and

B.3 $$\begin{aligned} |a_n|\ge (2n)!. \end{aligned}$$

### Proof

To prove the lemma, we look for a recurrence to define the coefficients of $\Phi$. First note that by Theorem 2.8 it must be of the form

$$\begin{aligned} {\hat{\Phi }}(z)=\frac{1}{z}+{\mathcal {O}}\left( \frac{1}{z^3}\right) \end{aligned}$$

It is straightforward to see from (B.1) that the series has only odd powers. We obtain that

$$\begin{aligned} \begin{aligned} a_{n+1}=&\frac{1}{(2n+3)(2n+4)-6}\Bigg [-(2n+1)(2n+2)(2n+3)(2n+4)a_n\\&+6\sum _{\begin{array}{c} k_1,k_2\ge 1\\ k_1+k_2=n+1 \end{array}}a_{k_1}a_{k_2}+2\sum _{\begin{array}{c} k_1,k_2,k_3\ge 1\\ k_1+k_2+k_3=n+1 \end{array}}a_{k_1}a_{k_2}a_{k_3}\Bigg ], \end{aligned} \end{aligned}$$

which, by induction, implies $a_n\in {\mathbb {R}}$ and (B.2).

Moreover, for all $n\ge 0$,

$$\begin{aligned} \begin{aligned} |a_{n+1}|\ge&\frac{(2n+1)(2n+2)(2n+3)(2n+4)}{(2n+3)(2n+4)-6}|a_n|\\ \ge&(2n+1)(2n+2)|a_n|, \end{aligned} \end{aligned}$$

which implies (B.3). $$\square $$

The fact that $\Theta \ne 0$ is a direct consequence of Lemma B.1. By the third statement of Theorem 2.8, it is enough to prove that there exists $z_0\in {\mathcal {R}}_{\theta ,\kappa }^\textrm{in}$ such that $\Delta \phi ^0(z_0)\ne 0$, or equivalently

$$\begin{aligned} \Phi ^\textrm{u}(z_0)-\Phi ^\textrm{s}(z_0)\ne 0. \end{aligned}$$

We argue by contradiction. Assume that $\Phi ^\textrm{u}(z)=\Phi ^\textrm{s}(z)$ for all $z\in {\mathcal {R}}_{\theta ,\kappa }^\textrm{in}$. Since, by Theorem 2.8, $\Phi ^\textrm{u}$, $\Phi ^\textrm{s}$ are real-analytic, they must coincide also in

$$\begin{aligned} \overline{{\mathcal {R}}}_{\theta ,\kappa }^\textrm{in}: =\left\{ z:\overline{z}\in {\mathcal {R}}_{\theta ,\kappa }^\textrm{in}\right\} . \end{aligned}$$

Therefore, the functions $\Phi ^\textrm{u}$, $\Phi ^\textrm{s}$ can be analytically extended to the neighborhood of infinity $|z|\ge \kappa$ and, thus, are analytic at infinity. This contradicts the fact that the asymptotic series of these functions at infinity have coefficients growing faster than a factorial.

## Appendix C. The Right Inverses of ${\mathcal {L}}_1$

Here we prove Lemmas 3.4, 3.6, 4.3, and 4.4.

### C.1. Proof of Lemmas 3.4 and 4.3

We first prove Lemma 3.4 in Section C.1.1. Then, we prove Lemma 4.3 in Section C.1.2 as an straightforward consequence of Lemma 3.4.

#### C.1.1. Proof of Lemma 3.4

Let $\zeta _1 (x)= u_0'(x)$. In $D^{\textrm{out},\textrm{u}}_\kappa$, it only vanishes at $x=0$ (see 1.7). We rewrite (3.3) as

$$\begin{aligned} \left( \frac{\zeta _2}{\zeta _1} \right) ' = \frac{1}{\zeta _1^2}, \end{aligned}$$

which is equivalent at the domain $D^{\textrm{out},\textrm{u}}_\kappa \backslash \{0\}$. For $x\in B_r \subset \mathbb {C}$, the open ball centered at the origin of radius *r*,

$$\begin{aligned} \zeta _1(x) = \sum _{k=1}^\infty c_k x^{2k-1}, \qquad c_1 \ne 0. \end{aligned}$$

Therefore, writing $\widehat{\zeta }_2 = \zeta _2 \zeta _1^{-1}$ we have that

$$\begin{aligned} \widehat{\zeta }_2'(x) = \frac{1}{\zeta _1^2(x)}=\frac{1}{c_1 x^2} \sum _{k=0}^{\infty } d_k x^{2k} \end{aligned}$$

which implies that

C.1 $$\begin{aligned} \widehat{\zeta }_2 (x) = -\frac{1}{c_1 x} + c_0+ \sum _{k=1}^{\infty } \frac{d_k}{2k} x^{2k-1}, \qquad x\in B_r. \end{aligned}$$

As a consequence, taking $c_0=0$ yields

C.2 $$\begin{aligned} \zeta _2 (x) = \widehat{\zeta }_2(x) \zeta _1(x) = -1 + \sum _{k=1} {\hat{c}}_k x^{2k}, \qquad x \in B_r, \end{aligned}$$

which defines an even real analytic function in $B_r$. Notice that $\zeta _2(0)=-1\ne 0$. For $x\in D_{\kappa }^{\textrm{out},\textrm{u}} \backslash B_r$, we define $\zeta _2(x)$ as

C.3 $$\begin{aligned} \zeta _2(x) = {\left\{ \begin{array}{ll} \zeta _1(x) \left[ \widehat{\zeta }_2(r) + \int _{r}^x \frac{1}{\zeta _1^2(s)}\, ds \right] &  \text {if } \Re x\ge 0, \\ \zeta _1(x) \left[ \widehat{\zeta }_2(-r) + \int _{-r}^x \frac{1}{\zeta _1^2(s)}\, ds \right] &  \text {if } \Re x< 0, \end{array}\right. } \end{aligned}$$

with $\widehat{\zeta }_2$ defined in (C.1), which is the even analytic extension at $D_{\kappa }^{\textrm{out},\textrm{u}}$ of $\zeta _2$ defined in (C.2).

We notice that since $\zeta _1 =u_0'\in \mathcal {E}_{1,2}$, then for $x\in D^{\textrm{out},\textrm{u}}_\kappa \cap \{\Re x \le -10\}$,

$$\begin{aligned} |\zeta _2 (x)| \lesssim \frac{1}{|\cosh x|} \left[ 1 + \int _{\Re x}^{-r} \cosh ^2 s \, ds \right] \lesssim \cosh \Re x \lesssim |\cosh x|, \end{aligned}$$

where we have used $\cosh \Re s \lesssim |\cosh s|\lesssim \cosh \Re s$.

When $x\in D^{\textrm{out},\textrm{u}}_\kappa \cap \{\Re x \ge -10\}$, $|\zeta _2(x)| \lesssim |\zeta _1(x)|$ and we conclude that $\zeta _2 \in \mathcal {E}_{-1,2}$.

#### C.1.2. Proof of Lemma 4.3

On $D_\kappa ^\textrm{aux}$, see (2.10) and Fig. 3, $\zeta _1$ has simple zeroes at $0,i \pi , -i \pi$. Then, denoting $x_0=0,i\pi ,-i\pi$, one has $\zeta _1(x)=\zeta _1'(x_0) (x-x_0) + \mathcal {O}(x-x_0)^2$ with $\zeta _1'(x_0)\ne 0$, and, as a consequence, when *x* goes to $x_0$ in definition (C.3) of $\zeta _2$, we have

$$\begin{aligned} \lim _{x\rightarrow x_0} \zeta _2(x)=\lim _{x\rightarrow x_0} \zeta _1(x) \int _{\pm r}^x \frac{1}{\zeta _1^2(s)} \, ds = -\frac{1}{\zeta _1'(x_0)}. \end{aligned}$$

In addition, $x_0$ do not belong to the segment between $x\in D_\kappa ^\textrm{aux}$ and $\pm r$ and then we conclude that $\zeta _2$ defined in (C.3) is, in fact, well defined and real analytic also at $D_\kappa ^\textrm{aux}$. Finally, using that $\zeta _1=u_0' \in {\mathcal {D}}\mathcal {Y}_2^1$, where $D\mathcal {Y}_\ell ^1)$ is defined by  (4.2), we obtain the result.

### C.2. Fundamental solutions of $\mathcal {L}_1[\zeta ]=0$

Here we provide new sets of fundamental solutions of the linear second order differential equation $\mathcal {L}_1[\zeta ]=0$, where $\mathcal {L}_1$ is defined in (2.4). We mainly follow the strategy in [31], being the first result below an adaptation of Lemma A.1 in [31].

We fix the complex rectangle

C.4 $$\begin{aligned} R=\{x\in \mathbb {C}: \quad -10 \le \Re x \le 0,\, \;\; |\Im x| \le 2\pi \} \end{aligned}$$

and we emphasize that, by Lemma 2.1$\zeta _1 =u_0'$ is analytic in $R\backslash \{x_-,\overline{x_-}\}$.

#### Lemma C.1

Let

$$\begin{aligned} \zeta _{+} (x) = \zeta _1(x) \int _{x_{-}}^x \frac{1}{\zeta _1^2(s)} \, ds, \qquad \zeta _{-} (x) = \zeta _1(x) \int _{\overline{x_{-}}}^x \frac{1}{\zeta _1^2(s)} \, ds. \end{aligned}$$

Then,

- $\zeta _{\pm }$ are analytic solutions of ${\mathcal {L}}_1[\zeta ]=0$ in the domain $R\backslash \{x_-,\overline{x_-}\}$ satisfying
  $$\begin{aligned} W(\zeta _+,\zeta _-) = \zeta _+ \zeta _-' - \zeta _+'\zeta _-=\int _{x_-}^{\overline{x_-}} \frac{1}{\zeta _1^2 (s)} \, ds \ne 0. \end{aligned}$$
- They satisfy, for $x\in R$ with *R* defined in (C.4),
  C.5 $$\begin{aligned} \zeta _{+}(x)= \frac{ (x- x_- )^3}{(x-\overline{x_-})^2} \widehat{\zeta }_+(x), \qquad \zeta _{-}(x)= \frac{ (x- \overline{x_-} )^3}{(x-x_-)^2} \widehat{\zeta }_-(x) \end{aligned}$$
  where $\widehat{\zeta }_{\pm }$ are analytic functions in *R* and $|\widehat{\zeta }_{\pm }(x)|\le M$ for some constant *M* (independent on *x*).
- For some constant *c*, we have
  C.6 $$\begin{aligned} \zeta _1(x) = \frac{1}{W(\zeta _+,\zeta _-)} \big (\zeta _+(x) - \zeta _-(x) \big ),\qquad \zeta _2(x) = c \zeta _1(x) + \zeta _-(x). \end{aligned}$$

#### Proof

On the rectangle *R* in (C.4), the function $\zeta _1(x)=u_0'(x)$, see  (1.7), has simple zeroes only at $x=0,\pm i \pi , \pm i 2\pi$, that is, writing $x_0=0,i\pi ,-\pi$, $\zeta _1(x) = \zeta _1'(x_0) (x-x_0) + \mathcal {O}(x-x_0)^2$ when *x* is close to $x_0$. Moreover, for all $x\in R$, the segments $\overline{x, x_-}$ and $\overline{x,\overline{x_-}}$ do not cross $x_0$. Then, since $\zeta _1'(x_0)\ne 0$,

$$\begin{aligned} \lim _{x\rightarrow x_0} \zeta _\pm (x) = -\frac{1}{\zeta _1'(x_0)} \end{aligned}$$

that implies that $\zeta _\pm$ are well defined at the set *R*. In addition, the fact that $\zeta _1^{-2}$ has zeroes of order 4 at $x_-,\overline{x_-}$ and it is uniformly bounded at *R*, implies that the estimates in (C.5) follow immediately and hence the second item of Lemma C.1 is already proven.

From the definition of $\zeta _\pm$, one can easily compute $W(\zeta _+,\zeta _-)$. We check that it is not zero. Indeed, we define

$$\begin{aligned} \widetilde{u}_0(t) = u_0(-\alpha + i t) = \frac{3}{\cos t +1 - 3 \sqrt{|\gamma |} i\sin t } \end{aligned}$$

and, after some tedious computations, we have that

$$\begin{aligned} \frac{1}{(u_0'(-\alpha +it))^2} = -\frac{1}{(\widetilde{u}'_0(t))^2 }= \frac{(\cos t +1 - 3 \sqrt{|\gamma |} i \sin t)^4}{9 (\sin t+ 3\sqrt{|\gamma |}i \cos t)^2}. \end{aligned}$$

Then, again performing some tedious but straightforward computations, we obtain

$$\begin{aligned} \int _{x_-}^{\overline{x_-}} \frac{1}{\zeta _1^2(s)} \, ds = -i \int _{\pi }^{-\pi } \frac{1}{(\widetilde{u}_0(t))^2 }\, dt = 3\pi i \left( |\gamma | - \frac{5}{9} \right) . \end{aligned}$$

This ends the proof of the first item of Lemma C.1.

Finally, we prove the third item of Lemma C.1. By the first item, $\zeta _+,\zeta _-$ are independent solutions of $\mathcal {L}_1[\zeta ]=0$, so that $\zeta _1= c_1 \zeta _- + c_2 \zeta _+$. Evaluating at $x_-,\overline{x_-}$ we obtain the coefficients $c_1,c_2$ and the formula for $\zeta _1$. On the other hand, $\zeta _2$ is a linear combination of $\zeta _+,\zeta _-$, which yields (C.6) since $W(\zeta _1,\zeta _2) \ne 0$. $\square$

Now we study

C.7 $$\begin{aligned} J_\pm (x):=\left| \zeta _\pm (x) \int _{0}^{x} \zeta _\mp (s) h(s) \, ds \right| , \end{aligned}$$

which play a key role when bounding the norm of the linear operators ${\mathcal {G}}_1, \widetilde{{\mathcal {G}}}_1$ defined in (3.5) and (4.3) respectively. Since these operators are defined over analytic functions in different domains, we introduce a new class of domains that posses the minimal properties we need to be able to bound $J_\pm$.

#### Definition C.2

Let $D \subset R$, with *R* defined in (C.4), be a closed bounded domain satisfying that

- $$0\in \textrm{int}(D)$$, $$x_-, \overline{x_-} \notin D $$,
- if $x \in D$, then $\Re x \in D$ and the segments $\overline{0,x} \in D$, $\overline{x, \Re x }\subset D$,
- there exists a constant $\vartheta \in (0,1)$ such that if $x\in D$ either $|\Im x |< \pi$, or
  $$\begin{aligned} |\Re x + \alpha | \ge \vartheta \min \{ |x- x_-|, |x-\overline{x_-}|\}. \end{aligned}$$

#### Remark C.3

Notice that $D_{\kappa }^{\textrm{out},\textrm{u}}\cap \{-10<\Re < 0\}$ in  (2.7) and $D_\kappa ^{\textrm{aux}}$ in  (2.10) satisfy the conditions in Definition C.2.

#### Lemma C.4

Let *D* be a domain satisfying the conditions in Definition C.2 and fix $\ell \ge 5$. If $h:D\rightarrow \mathbb {C}$, then

$$\begin{aligned} \left| J_\pm (x) \right| \lesssim \frac{\lfloor h\rfloor _{\ell }}{|x-x_-|^{\ell -2 } |x- \overline{x_-}|^{\ell -2}}, \qquad x\in D, \end{aligned}$$

where $J_\pm$ has been introduced in (C.7) and

$$\begin{aligned} \lfloor h \rfloor _\ell = \sup _{x\in D} |h(x)| |x-x_-|^{\ell } |x- \overline{x_-}|^\ell . \end{aligned}$$

#### Proof

We recall that $x_-=-\alpha + i \pi$ with $\alpha >0$. We only provide the details for $J_+$ being the corresponding for $J_-$ analogous. When $x\in D \cap \{ x\in \mathbb {C}: \Re x\ge -\frac{\alpha }{2}\}$, then, using the second item in Lemma C.1,

$$\begin{aligned} \begin{aligned} \left| \zeta _+(x) \int _{0}^{x} \zeta _-(s) h(s) \, ds \right|&\lesssim \frac{|x-x_-|^3}{|x-\overline{x_-}|^2 } \int _{0}^x \frac{\lfloor h \rfloor _{ \ell }}{|s-x_-|^{\ell +2 } |s-\overline{x_-}|^{\ell -3}} \\&\lesssim \frac{\lfloor h \rfloor _{ \ell }}{|x-x_-|^{\ell -2 } |x- \overline{x_-}|^{\ell -2}}. \end{aligned} \end{aligned}$$

Now we deal with $x\in D\cap \{ x\in \mathbb {C}: \Re x<-\frac{\alpha }{2}\}$. Since, by Lemma C.1, $\zeta _\pm$ and *h* are analytic functions in $D\subset R$, we write

$$\begin{aligned} \zeta _+(x)\int _{0}^x \zeta _- (s) h(s) \, ds&= \zeta _+(x)\left[ \int _{\gamma _1} \zeta _-(s) h(s)\, ds + \int _{\gamma _2} \zeta _-(s) h(s)\, ds \right] \\&=: G_1(x) + G_2(x), \end{aligned}$$

with $\gamma _1(t)= -t$, for $t\in [0, -\Re x]$ and $\gamma _2(t) = \Re x +it$, for $t\in \overline{0, \Im x}$. Notice that, by Definition C.2 of *D*, the paths $\gamma _1,\gamma _2 \subset D$. Then, we obtain

$$\begin{aligned} |G_1(x)|=\left| \zeta _+(x) \int _{\gamma _1} \zeta _-(s) h(s) \, ds \right|&\lesssim \frac{|x-x_-|^3}{|x-\overline{x_-}|^2 }\left| \int _{0}^{|\Re x|} \frac{\lfloor h \rfloor _{ \ell }}{|t +x_-|^{\ell +2 } |t+\overline{x_-}|^{\ell -3}} \right| \\  &\lesssim \frac{\lfloor h \rfloor _{ \ell }}{|x-x_-|^{\ell -2 } |x- \overline{x_-}|^{\ell -2}}, \end{aligned}$$

where we have used that $|t + x_-|, |t + \overline{x_-}| \ge \pi$ and that $|x|\lesssim 1$.

With respect to $G_2$, we have that

$$\begin{aligned} |G_2(x)| \lesssim \lfloor h \rfloor _\ell \frac{|x-x_-|^3}{|x-\overline{x_-}|^2 } \left| \int _{0}^{\Im x} \frac{1}{ |\Re x +i t - x_-|^{\ell +2} |\Re x +i t-\overline{x_-}|^{\ell -3} }\, dt \right| . \end{aligned}$$

Then, if $\Im x\ge 0$, since $|\Re x + i t - \overline{x_-}|\ge \pi$, for $t\in [0,\Im x]$, we have that

$$\begin{aligned} |G_2(x)| \lesssim \lfloor h \rfloor _\ell \frac{|x-x_-|^3}{|x-\overline{x_-}|^2 } \int _{0}^{\Im x} \frac{1}{\big ( (\Re x + \alpha )^2 + (t-\pi )^2 \big )^{\frac{\ell +2}{2}} }\, dt. \end{aligned}$$

In the case $|\Re x + \alpha | \ge \vartheta |x- x_-|$,

$$\begin{aligned} |G_2(x) |&\lesssim \lfloor h \rfloor _\ell \frac{|x-x_-|^3}{|x-\overline{x_-}|^2 } \frac{1}{|\Re x + \alpha |^{\ell +1}} \int _{-\infty }^{+\infty } \frac{1}{ (1 + t^2)^{\frac{\ell +2}{2}}} \, dt \\&\lesssim \lfloor h \rfloor _\ell \frac{1}{|x-\overline{x_-}|^2 |x-x_-|^{\ell -2}}, \end{aligned}$$

and the result follows provided $\ell \ge 5$. If $|\Re x + \alpha |\le \vartheta |x-x_-|$, then $0\le \Im x <\pi$ and $\pi -\Im x \ge \sqrt{1-\vartheta ^2} |x-x_-|$. We obtain

$$\begin{aligned} |G_2(x)|\lesssim \lfloor h \rfloor _\ell \frac{|x-x_-|^3}{|x-\overline{x_-}|^2 } \int _{0}^{\Im x} \frac{1}{(\pi -t)^{\ell +2}} \, dt \lesssim \lfloor h \rfloor _\ell \frac{|x-x_-|^3}{|x-\overline{x_-}|^2 (\pi - \Im x)^{\ell +1}}, \end{aligned}$$

and the result follows trivially also in this case.

The details in the case $\Im x\le 0$ are left to the reader. $\square$

### C.3. Proof of Lemma 3.6

The result related to ${\mathcal {G}}_2$ defined in (3.6) is a straightforward consequence of Lemma 5.5 in [33].

We focus now on proving the results related to ${\mathcal {G}}_1$. To do so we follow the main ingredients in the proof of Proposition 4.3 in [31]. When $x\in D^{\textrm{out},\textrm{u}}_\kappa \cap \{\Re x \le -10\}$, by Lemma 3.4, $|\zeta _2 (x)| \lesssim |\cosh x|$. From here, using also that $|\zeta _1(x) | \lesssim |\cosh x|^{-1}$ and following exactly the same steps as the ones in [31], we prove that

$$\begin{aligned} | \cosh x|^{m} \big |{\mathcal {G}}_1[h](x) \big | \lesssim \Vert h\Vert _{m,\ell }, \qquad x\in D^{\textrm{out},\textrm{u}}_\kappa \cap \{\Re x \le -10\}. \end{aligned}$$

The case $x\in D^{\textrm{out},\textrm{u}}_\kappa \cap \{\Re x \ge -10\}$ is more involved. Indeed, the main obstacle to overcome is that $\zeta _1,\zeta _2$ have poles of order 2 at $x=x_-, \overline{x_-}$. Following [31] we rewrite ${\mathcal {G}}_1$ in (3.5) in terms of $\zeta _+,\zeta _-$ in Lemma C.1. Using the third item of this result, we obtain that

$$\begin{aligned} {\mathcal {G}}_1[h] (x) =&\frac{1}{W(\zeta _+,\zeta _-)} \left[ \zeta _+(x) \int _{0}^x \zeta _-(s) h(s) \, ds - \zeta _-(x) \int _{0}^x \zeta _+(s) h(s)\, ds\right] \\  &- \zeta _2(x) \int _{-\infty }^0 \zeta _1(s) h(s)\, ds. \end{aligned}$$

By Remark C.3, we can use the results in Lemma C.4 to bound the two first integrals defining $\mathcal {G}_1[h]$. To bound the third integral, we claim that is a convergent real integral and that $\Vert \zeta _2\Vert _{-1,2}\lesssim 1$. Then,

$$\begin{aligned} \left| \zeta _2 (x) \int _{-\infty }^0 \zeta _1(s) h(s)\, ds \right| \lesssim \left| \zeta _2 (x)\right| \Vert h\Vert _{m,\ell } \lesssim \frac{\Vert h\Vert _{m,\ell }}{|x-x_-|^2|x-\overline{x_-}|^2}. \end{aligned}$$

Again, using that $\ell \ge 5$, the first bound in Lemma 3.6 is proven. To prove $\Vert \partial _x \mathcal {G}_1[h]\Vert _{1,\ell -1}$ we proceed analogously. Indeed, we have that

$$\begin{aligned} \partial _x {\mathcal {G}}_1[h] (x) =&\frac{1}{W(\zeta _+,\zeta _-)} \left[ \zeta '_+(x) \int _{0}^x \zeta _-(s) h(s) \, ds - \zeta _-'(x) \int _{0}^x \zeta _+(s) h(s)\, ds\right] \\&- \zeta _2'(x) \int _{-\infty }^0 \zeta _1(s) h(s)\, ds, \end{aligned}$$

where

$$\begin{aligned} \zeta _+'(x)=\frac{(x-x_-)^2}{(x-\overline{x_-})^3} \widetilde{\zeta }_+(x), \qquad \zeta _-'(x)=\frac{(x-\overline{x_-})^2}{(x-x_-)^3} \widetilde{\zeta }_-(x) \end{aligned}$$

for $\widetilde{\zeta }_{\pm }$ are analytic functions uniformly bounded at *R*.

To complete the proof of Lemma 3.6, we just recall that, by Lemma 3.4, $\zeta _2$ is an even function.

### C.4. Proof of Lemma 4.4

We first notice that using relations (C.6) between $\zeta _1,\zeta _2$ and $\zeta _{+},\zeta _-$ we have that

C.8 $$\begin{aligned} \widetilde{{\mathcal {G}}}_1[h](x) = \frac{1}{W(\zeta _+,\zeta _-)} \left( \zeta _+(x)\int _{0}^x \zeta _-(s) h(s)\, ds - \zeta _-(x) \int _{0}^x \zeta _+(x) h(s) \, ds\right) \end{aligned}$$

and that by Remark C.3, we can apply the results in Lemma C.1 for $x\in D^{\textrm{aux}}_{\kappa } \cap \{ x \in \mathbb {C}: \Re x \le 0\}$. Then, we have

$$\begin{aligned} |\widetilde{{\mathcal {G}}}_1[h](x) | \lesssim \frac{\Vert h\Vert _{ \ell }}{|x-x_-|^{\ell -2 } |x- \overline{x_-}|^{\ell -2}}, \end{aligned}$$

so that, since $1 \lesssim |x-x_+|, |x-\overline{x_+}|$, for $x\in D^{\textrm{aux}}_{\kappa } \cap \{ x \in \mathbb {C}: \Re x \le 0\}$, we obtain

C.9 $$\begin{aligned} |\widetilde{{\mathcal {G}}}_1[h](x) | |x-x_-|^{\ell -2 } |x- \overline{x_-}|^{\ell -2} |x_-x_+|^{\ell -2 } |x- \overline{x_+}|^{\ell -2} \lesssim \Vert h \Vert _\ell . \end{aligned}$$

When $x\in D^{\textrm{aux}}_{\kappa } \cap \{ x \in \mathbb {C}: \Re x \ge 0\}$, we only need to define the new set of fundamental solutions of $\mathcal {L}_1[h]$ given by

$$\begin{aligned} \widetilde{\zeta }_+(x) = \zeta _1(x) \int _{x_+}^x \frac{1}{\zeta _1^2(s)}\, ds, \qquad \widetilde{\zeta }_-(x)= \zeta _1(x)\int _{\overline{x_+}}^x \frac{1}{\zeta _1^2(s)} \, ds \end{aligned}$$

and proceeding in an analogous way as for $x\in D^{\textrm{aux}}_{\kappa } \cap \{ x \in \mathbb {C}: \Re x \le 0\}$ to obtain the bound (C.9) for $x\in D_\kappa ^\textrm{aux}$. By definition (4.1) of the norm, $\Vert \widetilde{{\mathcal {G}}}_1[h]\Vert _{\ell -2 } \lesssim \Vert h\Vert _{\ell }$.

Differentiating (C.8) with respect to *x* and performing similar bounds as the previous one, we prove the result for $\partial _x {\mathcal {G}}_1[h]$.

For the operator $\widetilde{\mathcal {G}}_2$ in (4.3), we take $x\in D_\kappa ^\textrm{aux}$ be such that $\Re x\le 0$ since the case $\Re x\ge 0$ is analogous. In this case $1\lesssim |x-x_+|, |x-\overline{x_+}|$ and hence we have to prove

$$\begin{aligned} |{\mathcal {G}}_2(x)| \lesssim \varepsilon ^2 \frac{\Vert h\Vert _\ell }{|x-x_-|^\ell |x-\overline{x_-}|^\ell }. \end{aligned}$$

By definition (4.3) of ${\mathcal {G}}_2$ it is enough to prove that for $\Re x\le 0$,

C.10 $$\begin{aligned} \left| e^{ \pm i\varepsilon ^{-1} x}\int _{\mp i\rho }^x e^{\mp i\varepsilon ^{-1} s }h(s) \, ds \right| \lesssim \varepsilon \frac{\Vert h\Vert _\ell }{|x-x_-|^\ell |x-\overline{x_-}|^\ell }. \end{aligned}$$

We deal with the bound for the integral from $-i\rho$. To prove the second one is analogous. We write

$$\begin{aligned} e^{i\varepsilon ^{-1} x}\int _{-i\rho }^x e^{-i\varepsilon ^{-1} s }h(s) \, ds&=e^{i\varepsilon ^{-1} x} \int _{\gamma _1} e^{-i\varepsilon ^{-1} s }h(s) \, ds + e^{i\varepsilon ^{-1} x}\int _{\gamma _2} e^{-i\varepsilon ^{-1} s }h(s) \, ds\\&=: G_1(x) + G_2(x), \end{aligned}$$

where the paths $\gamma _1,\gamma _2$ are defined by

$$\begin{aligned} \gamma _1(t)=x+te^{ -i\vartheta }, \quad t\in \overline{0,-\textrm{sec} \vartheta \, \Re x }, \qquad \gamma _2(t) = it,\quad t \in \overline{\tan \vartheta \, \Re x,-\rho } \end{aligned}$$

with $\vartheta >0$ such that $\gamma _1(t) \in D_\kappa ^\textrm{aux}$. We recall that $\Re x\le 0$ and hence $1\lesssim |x-x_+|, |x-\overline{x_+}|$. Therefore,

$$\begin{aligned} |G_1(x)| \lesssim \Vert h \Vert _\ell \int _{0}^{\textrm{sec} \vartheta |\Re x|} \frac{e^{-\varepsilon ^{-1} t \sin \vartheta }}{|x - x_-+ t e^{-i\vartheta }|^{\ell }|x - \overline{x_-}+ t e^{-i\vartheta }|^{\ell }} \, dt. \end{aligned}$$

The geometry of the set $D_\kappa ^\textrm{aux}$ implies that

$$\begin{aligned} |x - x_- + t e^{-\i \vartheta }| \gtrsim |x-x_-|, \qquad |x - \overline{x_-} + t e^{-\i \vartheta }| \gtrsim |x-\overline{x_-}|, \end{aligned}$$

hence

$$\begin{aligned} |G_1(x)| \lesssim \frac{\Vert h \Vert _\ell }{|x-x_-|^\ell |x-\overline{x_-}|^\ell } \int _{0}^\infty e^{-\varepsilon ^{-1} t \sin \vartheta } \, dt \lesssim \varepsilon \frac{\Vert h \Vert _\ell }{|x-x_-|^\ell |x-\overline{x_-}|^\ell }. \end{aligned}$$

The bound for $G_2$ follows using the same arguments. It is clear that $|x-x_-+it|\gtrsim |x- x_-|$ and $|x-\overline{x_-}| \gtrsim |x-\overline{x_-}|$. Hence,

$$\begin{aligned} |G_2(x)|&\lesssim \Vert h \Vert _\ell \int _{-\rho }^{ -\tan \vartheta |\Re x|} \frac{e^{\varepsilon ^{-1} t}}{|x-x_-+ it|^{\ell }|x - \overline{x_-}+ it|^{\ell }} \, dt \\&\lesssim \frac{\Vert h \Vert _\ell }{|x-x_-|^\ell |x-\overline{x_-}|^\ell } \int _{-\infty }^0 e^{\varepsilon ^{-1} t}\, dt \lesssim \varepsilon \frac{\Vert h \Vert _\ell }{|x-x_-|^\ell |x-\overline{x_-}|^\ell }. \end{aligned}$$

As a consequence, (C.10) is proven.
